# Transcriptomic and proteomic signatures of stemness and differentiation in the colon crypt

**DOI:** 10.1038/s42003-020-01181-z

**Published:** 2020-08-19

**Authors:** Amber N. Habowski, Jessica L. Flesher, Jennifer M. Bates, Chia-Feng Tsai, Kendall Martin, Rui Zhao, Anand K. Ganesan, Robert A. Edwards, Tujin Shi, H. Steven Wiley, Yongsheng Shi, Klemens J. Hertel, Marian L. Waterman

**Affiliations:** 1 Department of Microbiology and Molecular Genetics, University of California Irvine, Irvine, CA 92697 USA; 2 Department of Biological Chemistry, University of California Irvine, Irvine, CA 92697 USA; 3 Institute for Immunology, University of California Irvine, Irvine, CA 92697 USA; 4 Biological Sciences Division, Pacific Northwest National Laboratory, Richland, WA 99354 USA; 5 Environmental Molecular Sciences Laboratory, Pacific Northwest National Laboratory, Richland, WA 99354 USA; 6 Department of Dermatology, University of California Irvine, Irvine, CA 92697 USA; 7 Department of Pathology and Laboratory Medicine, University of California Irvine, Irvine, CA 92697 USA

**Keywords:** Intestinal stem cells, Colon, Proteomics, Transcriptomics, Flow cytometry

## Abstract

Intestinal stem cells are non-quiescent, dividing epithelial cells that rapidly differentiate into progenitor cells of the absorptive and secretory cell lineages. The kinetics of this process is rapid such that the epithelium is replaced weekly. To determine how the transcriptome and proteome keep pace with rapid differentiation, we developed a new cell sorting method to purify mouse colon epithelial cells. Here we show that alternative mRNA splicing and polyadenylation dominate changes in the transcriptome as stem cells differentiate into progenitors. In contrast, as progenitors differentiate into mature cell types, changes in mRNA levels dominate the transcriptome. RNA processing targets regulators of cell cycle, RNA, cell adhesion, SUMOylation, and Wnt and Notch signaling. Additionally, global proteome profiling detected >2,800 proteins and revealed RNA:protein patterns of abundance and correlation. Paired together, these data highlight new potentials for autocrine and feedback regulation and provide new insights into cell state transitions in the crypt.

## Abstract (web summary)

Habowski et al. develop a sorting protocol for purification and comparative analysis of mouse colon stem cells and their progeny. By proteomic and transcriptomic analysis, they determine that lineage commitment is accompanied by a greater change in mRNA splicing and polyadenylation than in gene expression and they characterise signalling pathways involved in differentiation.

## Introduction

The intestinal crypt is a good model for studying how stem cells support a rapidly renewing tissue. Crypts are invaginating structures of single-layer epithelium in which stem cells reside in a supportive niche at the base where they produce daughter cells (progenitors). Progenitors move up the crypt to differentiate and replace mature cells that are dying at the mucosal surface—a process with an average lifespan of only 4–5 days^1^. Constant replacement maintains homeostasis and proper absorption of water and nutrients, but the fast timescale of birth-to-death places great demand on both stem and daughter cells. Stem cells are by necessity non-quiescent and rapidly dividing, and progenitor cells exhibit rapid loss of stemness and commitment to differentiation. Multiple studies have shown how absorptive and secretory cell types can respond to wounding by de-differentiation and repopulation of the stem cell compartment^2–5^. Although this de-differentiation process occurs promptly, it is unknown if these reverse changes in cell state and gain of stemness occur on a similar rapid timescale as loss of stemness.

Quantitative imaging and lineage-tracing tools have shown that newly produced progenitor cells lose stemness as they move from the stem cell niche into a compartment called the transit amplifying zone (TAZ)^6^. The progenitor’s first round of cell division and commitment to either an absorptive (AbsPro) or secretory (SecPro) lineage happens nearly simultaneously with entrance into this zone. These changes occur within minutes-to-hours of each other, suggesting that loss of stemness and choice of cell lineage are connected and directed by processes that occur on this timescale.

Several signal transduction systems are important for the early changes in cell state. A decrease in Wnt signaling and activation of the unfolded protein response (UPR) correlates with loss of stemness^7^. In addition, Notch signaling balances commitment to either the absorptive or secretory lineage through lateral inhibition signaling^4^. There has been a longstanding expectation that stem cells are defined by a unique transcriptome and that loss of stemness and lineage commitment are similarly defined by unique signatures. However, although signaling systems are capable of altering transcription, it is not known how much of the rapid changes in cell state are due to the turning ON/OFF of signal-targeted gene programs versus more immediate processes of co- and post-transcriptional processing, such as alternative mRNA splicing and alternative polyadenylation (APA)^8–10^. Each of these processes can quickly modify the nascent transcriptome and its attendant proteome by altering the coding sequences of mRNAs, the localization or interactions of mRNA and proteins, or by changing protein abundance through alterations in mRNA stability and/or protein translation rates^11–14^.

To study how transcription and post-transcription processes contribute to stemness and differentiation, it is necessary to separate stem cells, daughter cells, and their differentiated progeny. Multiple cell sorting protocols have been optimized to isolate stem cells, but each lack resolution of these three cell types^15,16^. For example, the transgenic stem cell lineage marker Lgr5-EGFP enables purification of GFP-bright stem cells, but a mosaic expression pattern of the transgene in the intestine has made it difficult to confidently separate daughter cells from GFP-negative stem cells and differentiated cells^17,18^. Single-cell RNA-sequencing captures the diversity when analyzing mixed cell populations and has been useful for defining intestinal lineage trajectories and diversity of mature cells (for example, enterocytes and enteroendocrine cells; EEC)^19–23^. However, its low sequencing depth misses moderate-to-lowly expressed transcripts and mRNA splicing and polyadenylation analyses are not yet reliable. Therefore, the transcriptome and proteome basis for loss of stemness and early commitment is unknown.

Here, we developed a new cell sorting protocol for purification and comparative analysis of colon stem cells, their immediate daughters (SecPro, AbsPro), and their differentiated cell types, including tuft cells, EEC, and enterocytes (Ent). The protocol can be used with non-transgenic mice of any strain and importantly, when coupled to bulk RNA sequencing and mass spectrometry-based global proteome profiling, can provide a deeper analysis of cellular transcriptomes and proteomes. Using this protocol, we found that while the transcriptome and proteome of each cell type are generally correlated, deeper analyses of the bulk RNA-seq data reveal that loss of stemness and lineage commitment are accompanied by a greater change in mRNA splicing and polyadenylation than in gene expression, a pattern that largely resolves as progenitor cells mature. Sequencing analysis also enabled higher resolution of signal transduction systems (Wnt and Myc signaling), environmental sensing pathways, and patterns of lineage distinction, including prostaglandins and Fgf signaling pathways. These patterns were seen at both the RNA and protein level and are likely key to understanding the processes of homeostasis, namely: (i) loss of stemness, (ii) lineage commitment, and (iii) signaling connections between mature cell types. We relate how these findings are relevant to the earliest events that happen during loss of stemness and we highlight ways in which mature cells might de-differentiate to re-acquire the state of stemness.

## Results

### Flow sorting purification of colon crypt cell populations

**Fig. 1:**
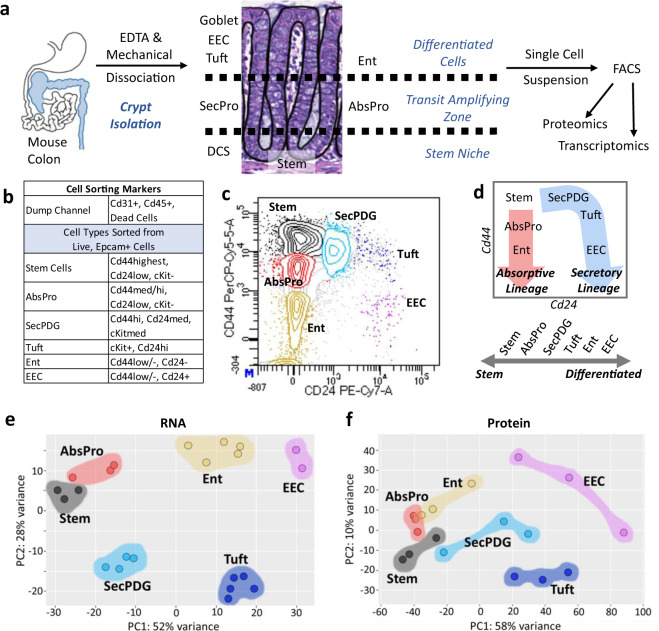
A novel flow sorting protocol that purifies six murine colon crypt cell populations. **a** Schema representing methods used for single cell isolation and, **b** sorting markers used for flow activated cell sorting (FACS). **c** FACS plot for membrane biomarkers Cd44 and Cd24 show six distinct populations including stem, absorptive progenitor (AbsPro), secretory progenitor/deep crypt secretory cells/goblet (SecPDG), tuft cells, enterocytes (Ent), and enteroendocrine (EEC). **d** Crypt cell populations diagramed in the FACS plot by lineage (secretory and absorptive) and on a scale from stem to differentiated. **e**, **f** Principle component analysis of **e**, bulk RNA-seq data with top 100 genes and **f**, proteomics data from the six crypt cell populations. For protein biological replicates for each cell type *n* = 3 samples, for RNA biological replicates, sample numbers are as follows: stem = 3, AbsPro = 3, SecPDG = 4, tuft = 5, Ent = 5, and EEC = 2.

To create a high-resolution profile of colon crypt stem cells and their daughter cells, we developed a new flow sorting protocol using freshly dissected, wild-type C57BL/6 N mouse colons and antibodies to validated intestinal cell surface markers including Cd44 (Fig. 1a, b, Supplementary Fig. 1). Upon discovery that Cd44 is highly sensitive to TrypLE, and other commonly used proteases^16^ (Supplementary Fig. 2a), we developed a dissociation protocol that uses only ethylenediaminetetraacetic acid (EDTA) and mechanical force. This change resulted in a 10-fold increase in detectable Cd44 antigen surface expression and therefore higher resolution for cell sorting (Fig. 1c, Supplementary Fig. 2b). Using additional commonly used cell surface markers, six cryptal populations could be isolated. A previously validated intestinal stem cell signature of Cd44-high, Cd24-low, and cKit-negative was used to identify and isolate an abundant fraction of stem cells (Fig. 1c). This cell population directly overlapped with Lgr5-EGFP+ cells from Lgr5-EGFP-IRES-creERT2 mice, confirming their stem cell identity^24^ (Supplementary Fig. 3a).

In addition to the stem cell population, five additional Epcam-positive populations were collected (Fig. 1c) and replicate biological samples of the six populations were processed for bulk RNA-seq (Supplementary Data #1). These populations represent cellular subtypes from two distinct cryptal lineages (secretory and absorptive), each revealing a trajectory of differentiation from stem to mature populations (Fig. 1d). Principal Component analysis (PCA) of mRNA and protein from the sorted cells showed that these cryptal populations were distinct and replicates tightly clustered (Fig. 1e, f). Known markers for colon crypt cells were used to identify cell types (Supplementary Fig. 4), which clearly demonstrated the presence of stem cells, two distinct populations of progenitor cells (absorptive and secretory), and three mature, differentiated populations (enterocytes, tuft cells and EECs). Thus, our new protocol for crypt isolation and the greater range of Cd44 surface expression it preserves, enables a meaningful improvement in the resolution and sorting of stem cells from daughter cells and differentiated progeny (Fig. 1c, d). Specifically, it is now possible to distinguish stem cells from AbsPro (Cd44^Med^) and from mature enterocytes (Ent; Cd44^Low/−^). Secretory progenitors were identified as SecPDG as this population contains mostly secretory progenitors and deep crypt secretory cells, with a possible minor contribution of goblet cells, a cell type that is largely missing from our isolated cells (SecPDG, Cd44^Med^, Fig. 1c, d, Supplementary Fig. 5). SecPDG markers were confirmed via immunohistochemical staining of human colon and small intestine (Supplementary Fig. 5). Finally, our protocol’s preservation of Cd44 expression, along with cKit expression, enabled resolution of two rare Epcam+/Cd24^high^ populations identified as tuft cells and EECs, which are mature cell types from the secretory lineage (Fig. 1c, d). EECs were predominantly enterochromaffin cells (Supplementary Fig. 6). Tuft cells were comprised of both Tuft-1 and Tuft-2 subtypes (Supplementary Fig. 7;^19^). For each of the isolated cell types we identified strongly associated biomarkers, including novel highly expressed proteins confirmed via proteomic analysis and immunohistochemistry (Supplementary Figs. 7–9, Supplementary Data #2). In the case of tuft cells, we detected taste-directed signaling pathways that are distinct from tuft cells in the small intestine (Supplementary Fig. 10)^25,26^.

Pairing the fluorescence-activated cell sorting (FACS) protocol with new methods for global proteome analysis of small numbers of cells (<200 cells) enabled us to compare the transcriptome and proteome for all six cell populations^27,28^ (Supplementary Data #2). Despite the use of small cell numbers, particularly, for the rare EEC and tuft cell populations, we were able to quantify the expression of over 2,800 proteins and investigate RNA:protein correlation patterns (Supplementary Figs. 11a, 12). General crypt markers, such as Epcam and Vil1 (Supplementary Fig. 11b), were detected along with markers of mature cell types (Supplementary Figs. 7g, 8g, 9g), and progenitor cell types (Supplementary Fig. 11c, d). We also confirmed that mRNA expression levels of the surface protein markers used in the FACS protocol could accurately cluster cell types (Supplementary Fig. 13), confirming that at least for the sorting markers, the mRNA and protein expression patterns are congruent. To determine whether our protocol is broadly useful we sorted colon epithelia from four additional commonly used mouse strains (Agouti, FVB, BALB/c, and NSG) and from female mice (Supplementary Fig. 14). The sorting results were nearly the same, demonstrating that the procedure reliably distinguishes colon crypt cell types regardless of mouse strain or gender.

### Redefining markers of crypt stem cells

**Fig. 2:**
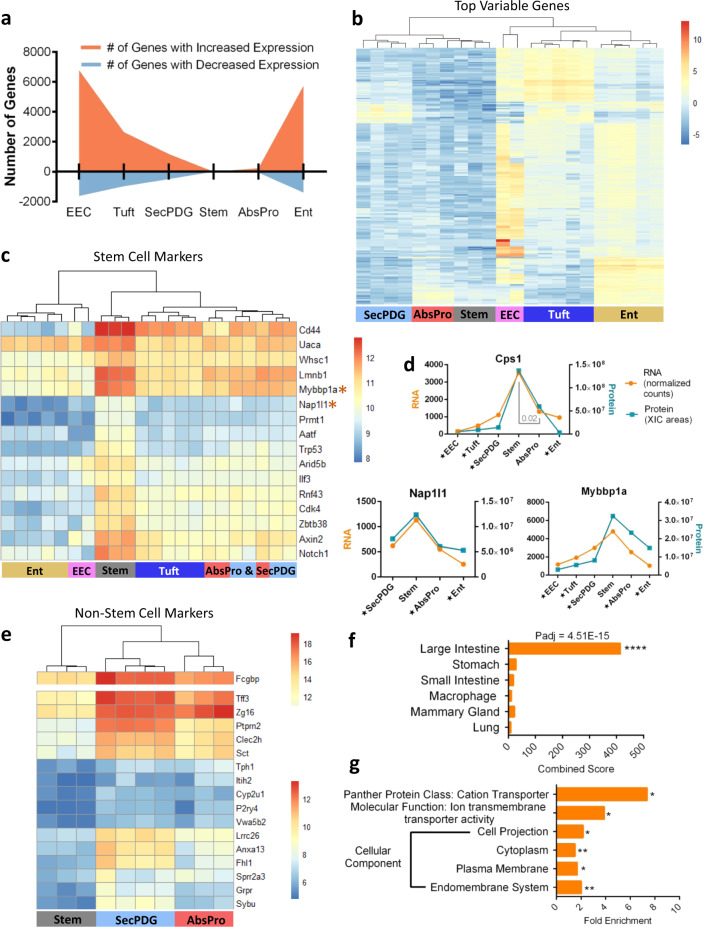
Characterization of intestinal stemness based on differential gene expression. **a** The number of genes that significantly change gene expression (mRNA level) between non-stem cells and stem cells; orange indicates the number of genes that increase expression and blue are the number of genes that decrease expression compared with stem (padj < 0.01 + minimum mean 50 counts). **b** Auto-scaled heatmap showing gene expression and unsupervised clustering of the top 200 most variably expressed genes. **c** Gene expression heatmap and unsupervised clustering of *n* = 16 genes that are significantly enriched in stem cells compared to all non-stem cells (padj  < 0.01+ minimum mean 50 counts). **d** Examples of stem enriched markers showing both mRNA expression paired with protein expression. Star annotation by cell type symbolizes significant differential mRNA expression compared with stem (padj  < 0.01). **e** Unsupervised clustering of genes that significantly increase in expression from stem to both SecPDG and AbsPro (8-fold change cutoff, padj  < 0.01 + minimum mean count 50 counts). Upper panel: the highly expressed *Fcgbp* gene is reported on a separate color scale. **f**, **g** Enrichr (Mouse Gene Atlas) and Panther (cellular component analysis, molecular function, and panther protein class) gene ontology analysis of *n* = 107 genes that are significantly higher in expression in all non-stem cell types compared with stem cells. FDR significance is defined by: *<0.05, **<0.01, ***<0.005, ****<0.001, and analysis was performed with the following biological replicate numbers: stem = 3, AbsPro = 3, SecPDG = 4, tuft = 5, Ent = 5, and EEC = 2.

The clear separation of stem cells from progenitors and mature cell types enabled us to define global patterns of gene expression from the early stages of loss of stemness and lineage commitment (comparing stem cells with AbsPro and SecPDG) to the final steps of differentiation (Ent, tuft, EEC; Fig. 2a). We observed several notable trends in gene expression. First, differentiation is generally accompanied by an increase rather than a decrease in gene expression (Fig. 2a). This is especially striking during loss of stemness and commitment to the absorptive and secretory lineages where there is a significant increase in the expression of 232 and 1177 genes in the absorptive and secretory progenitors, respectively, in contrast to a decrease in 69 and 492 genes in those populations (Fig. 2a; padj < 0.01 + minimum mean 50 counts). Fully committed, differentiated enterocytes, tuft cells, and EEC populations show 4.1, 2.7, and 4.2-fold differences in gene activation:suppression compared with stem cells, suggesting that differentiation in the colon crypt is defined more by gene activation rather than suppression of a distinct stem cell program. In addition, the transcriptomic stem cell signature is not shut off abruptly, but instead declines gradually (Fig. 2b). Thus, stem cells are defined more by the absence of differentiated cell markers. This applies to well-known intestinal stem cell markers such as *Lgr5, Smoc2, Cd44, Cdca7, Notch1*, and *Rnf43*, which show elevated expression in stem cells, but are well expressed in the other cell populations (Supplementary Fig. 15a). *Lgr5* is a notable example as its levels decrease by fourfold in AbsPro and SecPDG, but only twofold in the fully differentiated tuft cells demonstrating that *Lgr5* expression is not unique to the stem compartment (Supplementary Fig. 3b). Indeed, we could demonstrate *Lgr5* expression in tuft cells at the protein level using flow cytometry of colon crypt epithelia from Lgr5-EGFP-IRES-creERT2 (Supplementary Fig. 3c).

Although these data suggest that colon crypt stem cells have few specific markers, our analysis identified a set of 16 highly enriched mRNAs that distinguish stem cells from all other cell populations (Fig. 2c; padj  < 0.01 + minimum mean 50 counts). Some of these mRNAs are known stem cell markers (*Cd44, Rnf43, Notch1*) and Wnt signaling targets (*Axin2, Rnf43*), but newly identified markers are connected to epigenetics processes (*Lmnb1, Whsc1, Mybbp1a, Nap1l1, Prmt1, Aatf*, and *Arid5b*), regulation of the cell cycle (*Aatf, Cdk4, Trp53*—Supplementary Fig. 16), and transcription regulators (*Mybbp1a, Arid5b, Zbtb38*—Supplementary Fig. 17). Several markers were detected in the proteomics analysis as consistently elevated in stem cells (Fig. 2d). We also identified several additional protein markers that gradually decrease in protein and mRNA expression as cells transition to the progenitor stage (and thus do not pass our stringent significance cutoff of differentially expressed between stem and progenitor) (Supplementary Fig. 15b). RNA markers of proliferation (*Mki67, Pcna*, and *Mcms*) are highest in stem cells, but interestingly, their protein products are readily detectable in differentiated cells, thus highlighting inconsistencies between mRNA and protein biomarkers of proliferation (Supplementary Fig. 18). When we limit the differential gene expression analysis to a comparison of stem and daughter cells, SecPDG and AbsPro, there are an additional 11 mRNAs that are stem cell-enriched (Supplementary Fig. 15c), bringing the total number of genes that are most highly expressed in stem cells to 27. In contrast, the number of genes/proteins that increase as cells transition to the progenitor stage is larger. The top genes activated at this early step (e.g., *Fcgbp, Tff3, Ptprn2, Zg16*, etc.), are shown in Fig. 2e. If the comparison is extended to all cell types, there are 107 genes that significantly increase in expression in all cell stages and all cell types compared with stem cells (Supplementary Fig. 15d, example in Supplementary Fig. 15e, Supplementary Data #3). Gene ontology analysis (Enrichr and Panther) indicates these 107 “non-stem” genes demarcate the large intestine and are cytoplasmic and plasma membrane components (as opposed to factors in the nucleus), such as ion transporters that are involved in the function of mature epithelial cells in the mucosa (Fig. 2f, g).

### RNA processing remodels the intestinal crypt transcriptome

**Fig. 3:**
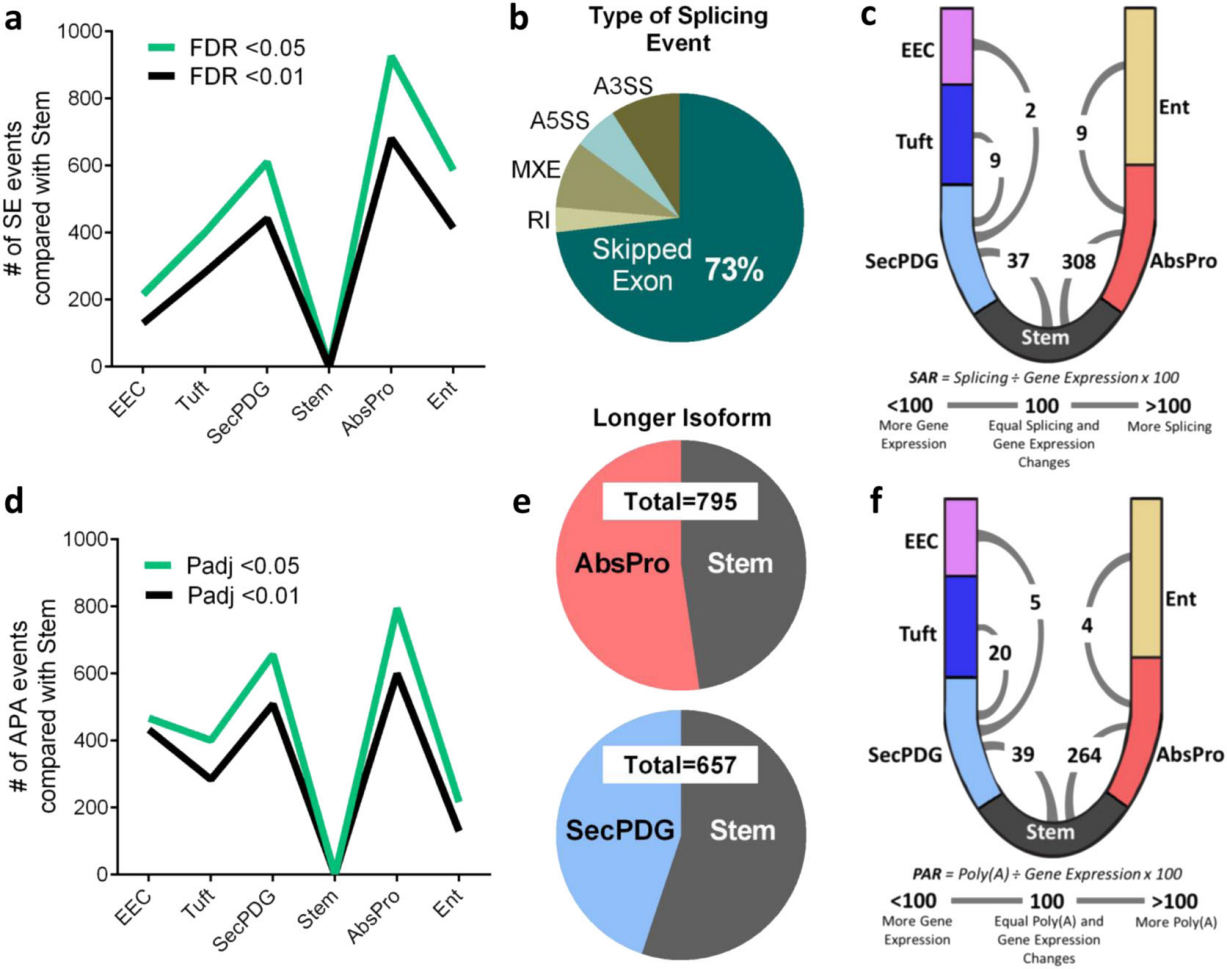
A burst of alternative mRNA processing activity during loss of stemness. **a** Alternative splicing analysis with rMATS determined the abundance of skipped exon events in non-stem cell types compared to stem cells. **b** Breakdown of average percentages of rMATS splicing changes (events) detected between stem and all non-stem cell types by type of event (SE = skipped exon, RI = retained intron, MXE = mutually exclusive exon, A5SS = Alt 5 splice site, A3SS = Alt 3 splice site) showing predominance of SE (73%) (FDR < 0.05). **c** Crypt diagram illustrating cell types in the secretory lineage (SecPDG, tuft, EEC) versus absorptive lineage (AbsPro, Ent). A numeric SAR (splicing abundance ratio = number of significant alternative splicing changes ÷ number of significant gene expression changes x 100) arc indicates the number of splicing changes relative to gene expression between stem, progenitors, and differentiated cells. **d** Alternative polyadenylation (APA) analysis with DaPars quantitated the number of APA changes (events) in non-stem cells compared with stem. **e** APA events characterized by which cell type has the longer 3’UTR isoform for each polyadenylated mRNA in stem versus AbsPro (top) and stem versus SecPDG (bottom) (padj  < 0.05). **f** Crypt diagram illustrating PAR (polyadenylation abundance ratio = number of significant alternative polyadenylation changes ÷ number of significant gene expression changes × 100) comparing polyadenylation changes to gene expression between stem, progenitors, and differentiated cells. Splicing and polyadenylation analysis was performed with the following biological replicate number of mRNA-seq samples: stem = 3, AbsPro = 3, SecPDG = 4, tuft = 5, Ent = 5, and EEC = 2.

Given that the majority of gene expression changes as measured by mRNA levels are gradual and do not sharply distinguish stem cells from progenitor cell states, we investigated whether other transcriptomic signatures better delineate the rapid transitions of loss of stemness and early commitment. As alternative pre-mRNA processing has been shown to be important in the differentiation of embryonic stem cells, we asked whether there are differences in alternative splicing and polyadenylation^29–38^. We used two computational pipelines, rMATS Turbo and MAJIQ, to analyze the RNA-seq data to identify significant changes in mRNA-splicing patterns among the six cell populations (Fig. 3a–c, Supplementary Fig. 19a; list of alternatively spliced genes in Supplementary Data #4)^39,40^. With rMATS, we identified 3,659 changes in mRNA splicing among all possible comparisons, with the vast majority of these changes detected as skipped exon (SE) events (Fig. 3b, Supplementary Fig. 19b, Supplementary Data #4). The largest number of alternative mRNA splicing events were during the transition from stem to AbsPro (926 SE events; rMATS, FDR  < 0.05), even though there are threefold fewer changes in gene expression (301 significant changes in mRNA levels, Fig. 2a). The relative number of changes in splicing compared with the number of changes in gene expression (mRNA level) can be represented by a splicing abundance ratio (SAR) (Fig. 3c; (number of significant alternative splicing changes ÷ number of significant gene expression changes × 100)). This metric reveals the extent to which splicing changes dominate the changes in the transcriptome during the transition from stem to AbsPro and stem to SecPDG. During the latter stages of differentiation, however, the number of alternative splicing events is much less than the number of gene expression changes (Fig. 3c; Supplementary Fig. 19c).

We next used an APA analysis platform, DaPars, to identify changes in APA and to determine the length of 3′ UTR regions in mRNAs (list of alternatively polyadenylated genes in Supplementary Data #4)^41^. Similar to the patterns of alternative splicing, the largest number of APA events were detected in the transition from stem to AbsPro, followed by stem to SecPDG (Fig. 3d). However, unlike APA changes observed during embryonic stem cell differentiation^34,37^, there is not a dominant, global trend toward lengthening or shortening of 3′ UTRs (Fig. 3e). Similar to SAR, a polyadenylation abundance ratio (PAR) was used to quantify the number of changes in polyadenylation relative to the number of changes in gene expression (Fig. 3f, Supplementary Fig. 19d). This analysis revealed a pattern similar to mRNA splicing in that there are a greater number of APA events compared with gene expression changes as stem cells transition through loss of stemness and lineage choice and fewer changes during the final stages of differentiation into mature cell types. These data suggest that pre-mRNA processing, rather than gene expression changes, remodels the transcriptome, and proteome during loss of stemness and/or lineage commitment.

### RNA processing in the loss of intestinal stemness

**Fig. 4:**
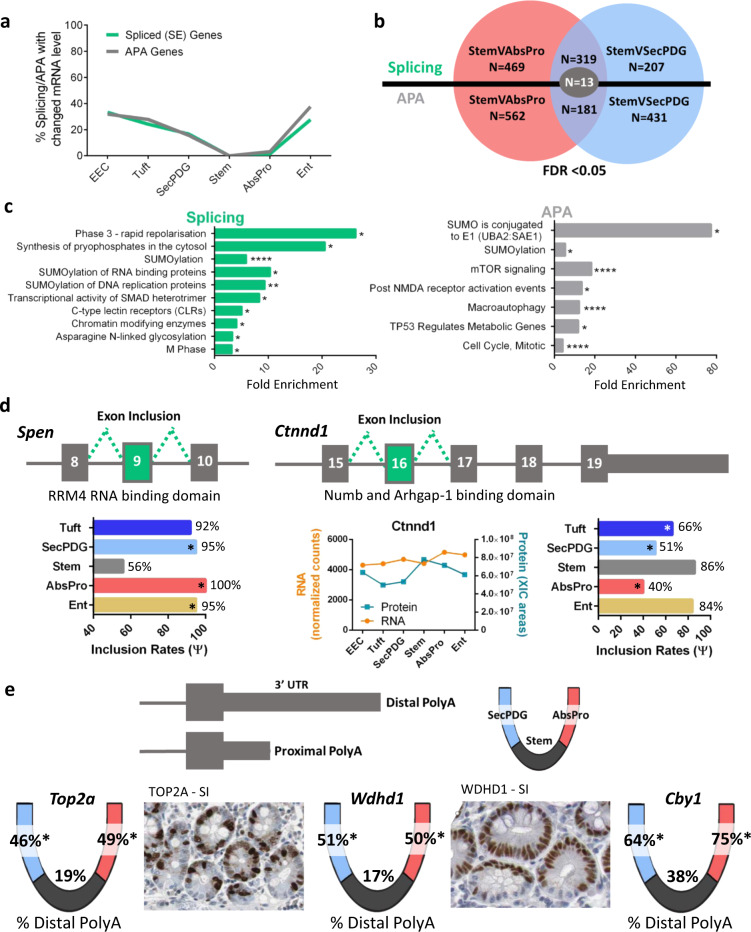
Alternative splicing and polyadenylation changes that occur during intestinal crypt loss of stemness. **a** Percentage of alternatively spliced (FDR < 0.05) or polyadenylated genes (padj < 0.05) that also change gene expression (padj  < 0.01) compared with stem. **b** Venn diagram overlap of APA and alternatively spliced genes between stem versus AbsPro and stem versus SecPDG (FDR  < 0.05). *n* = 13 genes were both APA and alternatively spliced differently in stem cells versus SecPDG and stem cells versus AbsPro (Supplementary Fig. 21a). **c** Gene ontology (reactome pathway) analysis of the commonly spliced genes when comparing stem versus AbsPro and stem versus SecPDG (rMATS; *n* = 332 (319 + 13) genes) and common APA genes (DaPars, *n* = 194 (181 + 13) genes); FDR  < 0.05. Sumoylation and cell cycle ontologies of alternatively processed genes are common to both splicing and APA changes (Supplementary Fig. 21b, c). **d** Two examples of alternatively spliced genes, *Spen* and *Ctnnd1*, which are differentially processed in stem cells versus progenitor cells. The exon inclusion rate for each event is shown in the bar graph. Ctnnd1 protein was detected by MS and the abundance compared with mRNA is displayed. **e** Three examples of genes with significant changes in alternative polyadenylation choice: *Top2a*, *Wdhd1*, and *Cyb1*. Overlaid on the crypt-base diagram are the percentage of distal polyA usage for each of the genes in the three cryptal cell compartments: stem, AbsPro, and SecPDG. Human protein atlas images show strong small intestine (SI) staining patterns of TOP2A and WDHD1 in the transit amplifying zone but a lack of staining in the stem cell niche despite the fact that *Top2a* mRNA levels are elevated in stem cells compared to progenitor cells and *Wdhd1* mRNA levels are the same among the cell types (Supplementary Fig. 22). Additional immunohistochemistry images of human intestine are provided in Supplementary Fig. 23. FDR significance is defined by: *<0.05, **<0.01, ***<0.005, ****<0.001.

RNA-processing activities can be influenced by regulators and transcription rates and therefore linked to changes in mRNA levels. Alternatively, RNA processing can be a separate regulatory network that modifies the sequences of the existing transcriptome without altering mRNA abundance. We observe that the latter is the case for early stages of differentiation. Fewer than 5% and 20%, respectively, of the AbsPro and SecPDG alternatively processed mRNAs showed significant changes in the level of mRNA (Fig. 4a). This suggests that during loss of stemness, alternative mRNA processing and activation of gene transcription are distinct regulatory programs. Because changes in RNA processing are more common than alterations in gene transcription, the functional role of the processed mRNAs could reveal important details about crypt stem cell biology and loss of stemness. Thus, we identified mRNAs that displayed differential processing in both AbsPro and SecPDG. These changes included 332 genes with SE events in common, and 194 genes with common APA events (Fig. 4b; the majority of these genes contain the same event). As these changes occur in both progenitor populations, they could potentially be some of the earliest changes in the stem cell transcriptome before lineage transition to an absorptive or secretory progenitor state. Gene ontology analysis of these commonly processed mRNAs shows the most dominant function is protein SUMOylation, such as SUMO enzymes and ‘SUMO conjugation to E1’ (Fig. 4c, Supplementary Figs. 20, 21b, c). Other enrichments include programs of mitosis, signaling (SMADs, mTOR, TP53, NMDA, ion channels), and glycosylation, with a number of these genes connected to Notch and Wnt signaling, which are pathways that direct stemness and differentiation in the intestine.

For example, split ends protein (Spen) has four RRM RNA-binding domains and functions in splicing and transcription regulation, including suppression of Notch and activation of Wnt signaling^42–46^. Approximately 50% of *Spen* mRNA in stem cells is missing the 4th RRM domain, whereas in the secretory and absorptive progenitor populations, this domain is present in ~100% of the *Spen* mRNA (Fig. 4d, Supplementary Fig. 22a). Delta-catenin (*Ctnnd1*) has known functions in adhesion as well as Wnt and Notch signaling (Fig. 4d, Supplementary Fig. 22a). A C-terminal domain of Ctnnd-1 that binds the Notch1 regulator Numb and the GTPase activator Arhgap-1 is more often encoded in *Ctnnd1* mRNA in stem cells than in progenitor populations. Three examples of APA differences between stem cells and daughter cells (*Top2a* (DNA replication), *Wdhd1* (DNA replication), and *Cby1* (Wnt signaling regulator)) show significant increases in distal polyA choice and lengthening of the 3′-UTR (Fig. 4e, Supplementary Fig. 22b). Interestingly, strong protein expression of Top2a and Wdhd1 is detected in the TAZ of crypts rather than at the base of the stem cell niche. (Supplementary Fig. 23).

Previous work using variant-specific antibodies demonstrated that two isoforms of integrin α6 (Itga6) are present in the crypt with Itga6 isoform A (inclusion of exon 25) being more abundant in the base of the crypt, and isoform B (skipping of exon 25) being more abundant near the top of the crypt (Supplementary Fig. 24a)^47^. Consistent with this, our analysis revealed that exon 25 has the highest inclusion in stem cells, and the lowest in SecPDG and Ent (Supplementary Fig. 24b). Our global proteomics assays did not detect these isoforms, but it does reveal uniformly high Itga6 protein expression in all cell types along with expression of other adhesion proteins (Supplementary Fig. 24c, d). Splicing of exon 25 alters the cytoplasmic domain of Itga6 (PDZ-binding domain) and has been linked to stem cell fate determination in several different model systems^48^.

### RNA processing in intestinal lineage commitment

**Fig. 5:**
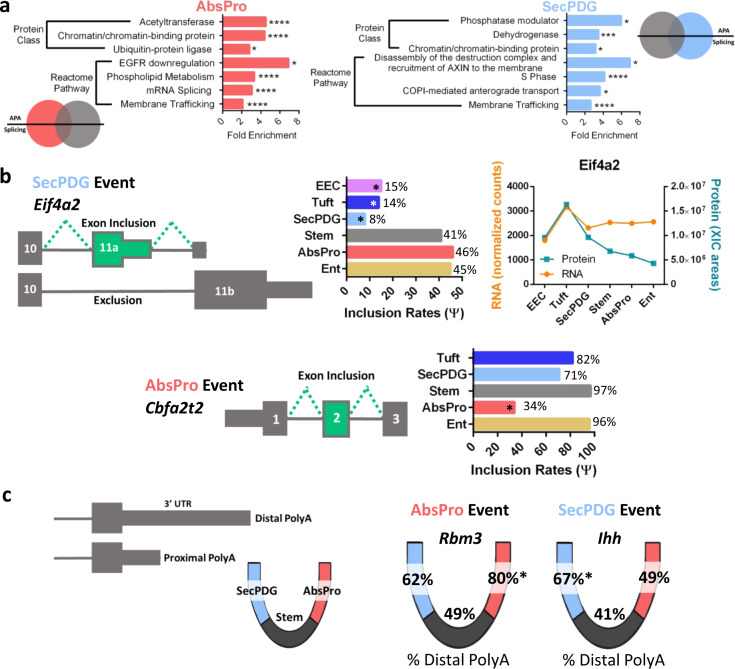
mRNA processing contributes to lineage commitment. **a** Gene ontology analysis (Panther) was performed on alternatively spliced (FDR < 0.05) and polyadenylated genes (padj < 0.05) with processing events specific to either AbsPro (red) or SecPDG (blue). Events are unique and not part of the overlap shown in Fig. 4b. **b** Two examples of alternatively spliced genes: *Eif4a2*, specific to SecPDG, and *Cbfa2t2*, specific to AbsPro. The exon inclusion rate for each event is shown in the bar graph. Eif4a2 was detected by MS and the abundance compared to mRNA is displayed (Cbfa2t2 protein was not detected). **c** Two examples of alternatively polyadenylated genes: *Rbm3*, AbsPro specific, and *Ihh*, specific to SecPDG. The percentage of distal polyA usage for each of the events is overlaid on the crypt-base diagram. FDR significance is defined by: *<0.05, **<0.01, ***<0.005, ****<0.001.

Commitment of progenitor cells to an absorptive or secretory lineage is a nearly simultaneous event with loss of stemness^6^—an event influenced by signals (e.g., Notch, Wnt, UPR, etc) that activate expression of lineage-specific genes. Significantly, in addition to common splicing and APA changes in both lineages, our analysis detected 469 and 207 lineage-specific changes in alternative mRNA splicing (AbsPro and SecPDG, respectively; see Fig. 4b). Similarly, 562 distinct changes in polyadenylation were detected in the AbsPro lineage and 431 changes in the SecPDG lineage (see Fig. 4b). These lineage-specific patterns suggest an important role for splicing and APA in specifying cell fate and lineage choice, and again, the number of processing changes exceeded the number of changes in gene expression (SAR, PAR ≥100; Fig. 3c, f). Functional analysis of the alternatively processed genes revealed that the predominant associated processes were chromatin binding and membrane trafficking (Fig. 5a). In addition, there were enriched functions connected to signaling (Egfr, Wnt), as well as splicing and cell cycle events (Fig. 5a–c).

The mRNA encoding the translation regulator *Eif4a2*, a DEAD box RNA helicase involved in translation repression^49^, is alternatively spliced in a lineage-specific manner. *Eif4a2* mRNA encodes a full-length protein isoform in the secretory populations (SecPDG, Tuft, EEC), whereas nearly half of the *Eif4a2* mRNA in the stem, AbsPro, and Ent populations encodes a truncated protein isoform (inclusion of exon 11a, Fig. 5b, Supplementary Fig. 22a). Total Eif4a2 protein levels in these populations are between two- to threefold less abundant suggesting that this processing, which truncates the open reading frame of *Eif4a2*, could influence protein abundance.

Exon 2 of *Cbfa2t2* (also known as *Mtgr1*) is largely missing in AbsPro mRNA (34% inclusion) but mostly present in SecPDG mRNA (71% inclusion) (Supplementary Fig. 22a). This protein is a transcription regulatory co-factor that interacts with co-repressors (e.g., Prdm14, Ncor, Hdacs, and Zbtb33 (Kaiso)), as well as transcription regulatory factors in the Notch (Rbpj) and Wnt (Lef/Tcfs) signaling pathways^50,51^. Although the functional consequence of this splicing event is not known, *Cbfa2t2* is known to be important in the secretory lineage, as knockout of *Cbfa2t2* leads to a loss of secretory cell types as well as a surge in cell proliferation of remaining cell populations^52^.

Although the functional consequences of many distal polyA choices are not known, two striking examples of changes in polyA choice in absorptive versus secretory lineage are shown in Fig. 5c (Supplementary Fig. 22b). *Rbm3* mRNA encodes an RNA binding protein that enhances Wnt signaling^53^, stemness and mRNA stability, and *Ihh* mRNA encodes a Hedgehog signaling ligand that opposes Wnt signaling in intestinal crypts^54^. Polyadenylation of *Rbm3* shifts to a more distal site in AbsPro (Wnt suppressed), whereas polyadenylation of *Ihh* mRNA is shifted to a more distal site in SecPDG (Wnt enhanced). Alternate processing of these genes could potentially contribute to the skewing of Wnt and Notch signaling activities in cells^55^.

### Gene expression changes in intestinal lineage commitment

**Fig. 6:**
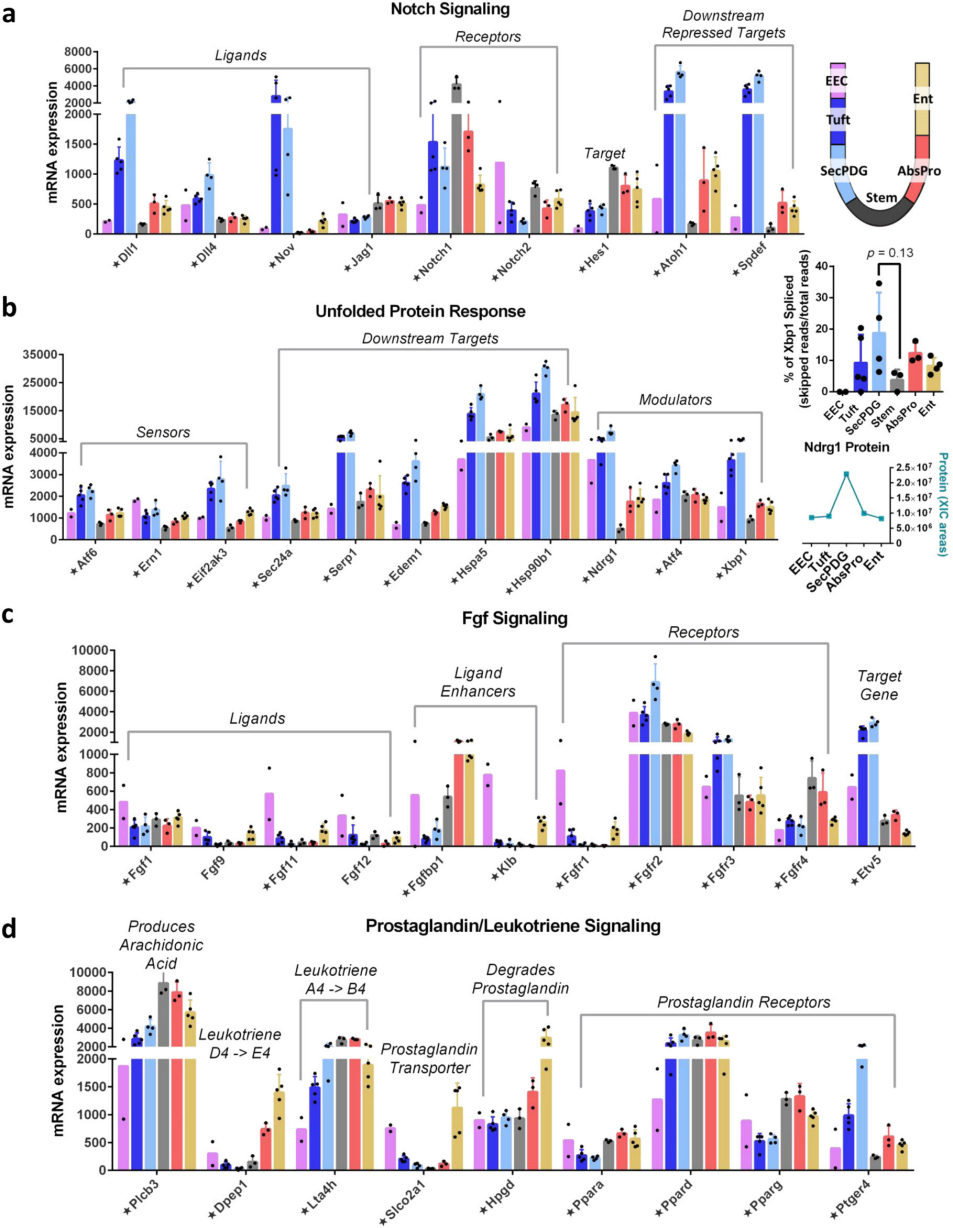
Lineage commitment to secretory and absorptive lineages are influenced by signaling pathways. **a** mRNA expression of Notch ligands (elevated in SecPDG/tuft), receptors (elevated in stem), and downstream targets. **b** mRNA expression of unfolded protein response (UPR) components including sensors, modulators, and downstream targets are elevated in SecPDG. Xbp1 activation, determined by a cytoplasmic splicing event, is elevated in SecPDG (inset—unpaired two-sided *t* test). Modulator Ndrg1 was detected via MS and shows protein is elevated in SecPDG (inset graph) consistent with mRNA expression (Ndrg1 protein was not detected in Stem). **c** mRNA expression of Fgf signaling components including ligands (showing some EEC expression), ligand enhancer *Fgfbp1* expressed in AbsPro and Ent, receptors (well expressed in all cells, highest in SecPDG and tuft) and target gene (highly expressed in SecPDG and tuft). **d** Prostaglandin and leukotriene precursors and final products are produced by tuft cells (Supplementary Fig. 25), but absorptive lineage cells AbsPro and Ent, might contribute to the production (*Plcb3*, *Dpep1*, and *Lta4h*) and degradation (*Slco2a1* and *Hpgd*) of prostaglandin signals. Prostaglandin receptor *Ptger4* is enriched in SecPDG, whereas alternate receptors *Pparg* and *Ppara* are enriched in the absorptive lineage. Star annotation by gene name symbolizes significant differential mRNA expression in at least one cell type compared with stem (padj < 0.01). mRNA expression values are normalized counts and error bars are standard deviation. mRNA differential expression analysis was performed with the following biological replicate numbers: stem = 3, AbsPro = 3, SecPDG = 4, tuft = 5, Ent = 5, and EEC = 2.

Although there are minimal gene expression changes during the initial loss of stemness and transition to progenitor states, changes in mRNA levels become increasingly apparent as progenitor cells differentiate. Our analyses not only identified well-established transcriptional signatures of loss of stemness (e.g., UPR) and lineage commitment steps (e.g., Notch), but also identified expression patterns suggesting additional autocrine/paracrine signaling that could impact lineage choice. For example, Notch signaling is known to direct lineage choice via lateral inhibition signaling in small intestinal crypts. Our RNA-seq data indicate that secretory lineage (SecPDG, tuft) cells express high mRNA levels for Notch ligands *Dll1* and *Dll4*, as well as a third ligand *Nov* (Fig. 6a). Stem and AbsPro populations express the *Notch1* receptor as well as the Notch target gene *Hes1*, showing that Notch signaling is activated to the greatest extent in stem cells and AbsPro^4^. Also consistent with lineage commitment in the small intestine, the Hes1-repressed target gene *Atoh1*, and its downstream target *Spdef* are expressed at the highest levels in the secretory lineage^4,56^. These expression patterns show that the populations we have characterized in the colon align with the Notch-directed lateral inhibition feedback loop identified in the small intestine wherein Notch signaling by secretory cells to absorptive cells balances the proportions of the two mature cell types.

UPR directs cellular responses to ER stress such as growth arrest, apoptosis and/or survival, and can trigger loss of stemness as intestinal stem cells exit their niche^7^. Although our analysis indicates activation of UPR in colon crypt progenitors, we observe that UPR signaling is lineage-skewed and most active in secretory populations (Fig. 6b). Active UPR, as evidenced by target gene expression, protein expression of modulator Ndgr1, and increased splicing of *Xbp1*, was observed mostly in SecPDG and tuft cells (Fig. 6b)^57^. Furthermore, the UPR signal appears to direct survival rather than growth arrest. Specifically, although genes for three UPR sensors (*Atf6, Ern1, Eif2ak3*) were detected in the secretory lineage, the downstream target genes for two of them—Atf6, Ern1 (*Hspa5* and *Hsp90b1*) displayed the highest expression in this lineage. These targets promote ER expansion and survival from stress. Taken together, the increased expression of sensors and downstream targets in the secretory lineage suggests a sensitization to UPR stress that might play a role in lineage choice and/or stabilization. Interestingly, ER stress can slow migration, consistent with recent observations that secretory progenitors migrate up the crypt at a slower rate than absorptive cells^58^.

Our analysis also discovered potentials for Fgf autocrine/paracrine signaling that could explain reported knockout phenotypes. Fgf has an important role in crypt homeostasis, although many Fgf ligands in adult mice come from the surrounding stroma^59,60^. Our transcriptional profiling indicated that only a few Fgf ligands are expressed by the epithelia (*Fgf1*, 9, 11, and 12), and predominantly by EECs. Fgf receptors, in contrast, are broadly expressed across the different cell types with *Fgfr3* detected at the highest level in the secretory lineages of SecPDG and tuft (Fig. 6c). Fgf target gene *Etv5* is most highly expressed in secretory cell types, indicating that the pathway is most active in this lineage (10-fold enriched in SecPDG and tuft; Fig. 6c).

Gene expression analysis also uncovered potential for lineage-specific autocrine/paracrine activities in prostaglandin signaling (Fig. 6d, Supplementary Fig. 25). Consistent with previous reports, we observed that tuft cells express key enzymes for prostaglandin and leukotriene synthesis, including *Ptgs1* (Cox-1), which converts arachidonic acid into prostaglandin H2 (Supplementary Fig. 25)^61^. Enzymes that convert prostaglandin H2 to the more stable E2 form (*Ptges2* and *Ptges3*) and the prostaglandin transporter *Abcc4* are expressed in all cell types (Supplementary Fig. 25d). *Ptger4*, a receptor for PGE2 is highly expressed in SecPDG (Fig. 6d, Supplementary Fig. 25d). Enterocytes express both an importer (*Slco2a*) for prostaglandins and an enzyme that degrades these molecules (*Hpgd*), suggesting that enterocytes might act as sinks for prostaglandin-mediated signals.

### Transcription regulators and signaling in mature crypt cells

**Fig. 7:**
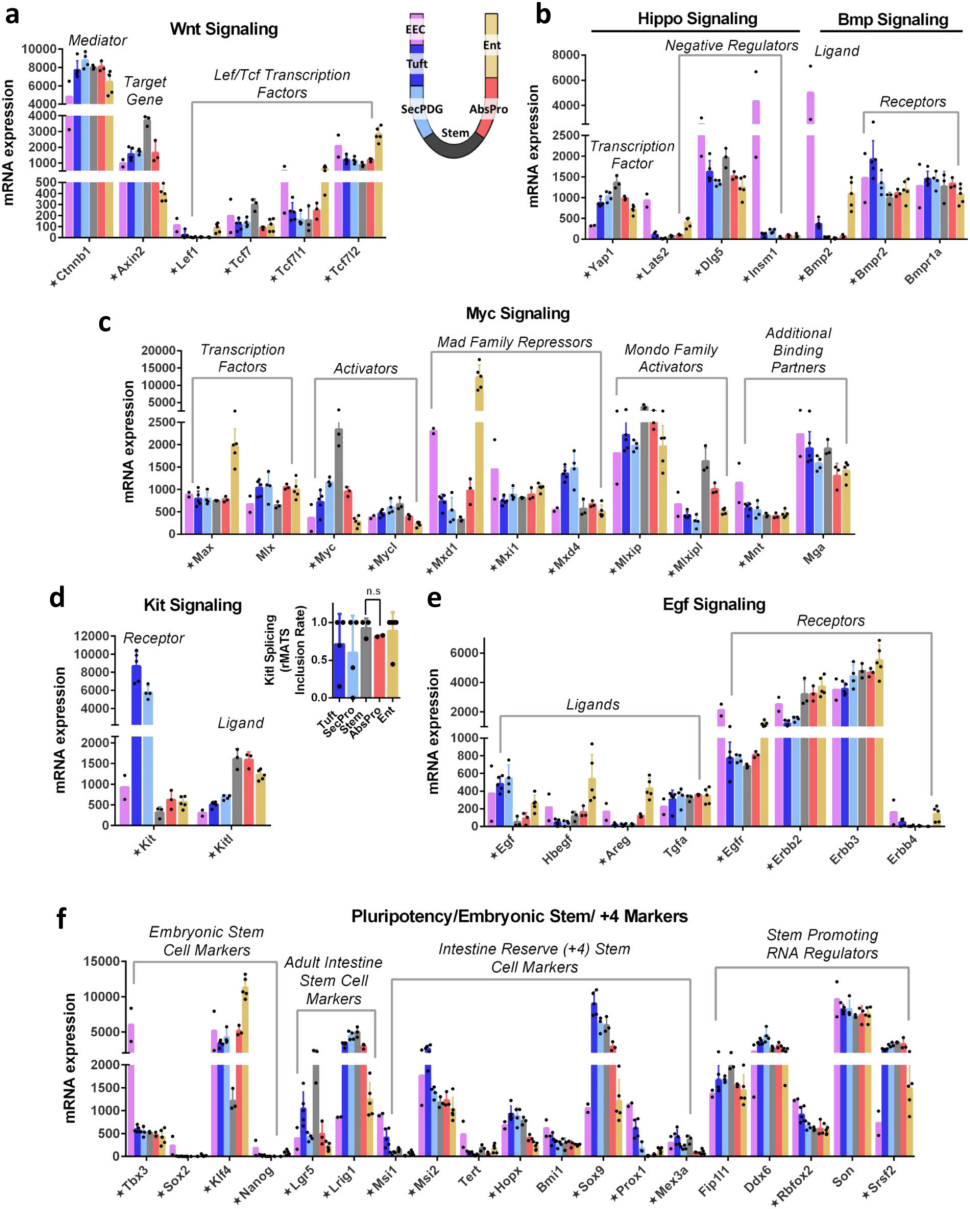
Fate commitment and signaling characteristics of mature crypt cells. **a** mRNA expression of key Wnt signaling factors including Lef/Tcf transcription factors, β-catenin mediator (Supplementary Fig. 27a), and downstream target gene *Axin2*. **b** mRNA expression of Hippo and Bmp signaling components. **c** mRNA expression of Myc signaling components including strong expression of repressive transcription factors. **d** mRNA expression of Kit signaling components including ligand (high in stem and absorptive), receptor (high in SecPDG and tuft). Splicing rates of *Kitl* in crypt cell types showed predominance of exon 6 inclusion, which encodes a protease cleavage site for release and secretion of Kit ligand (inset). **e** mRNA expression of Egf signaling components including select epithelial ligand expression and receptors expressed in all cells. *Egfr* is elevated in EEC (Supplementary Fig. 28), whereas the lowest levels of *Erbb2* is in SecPDG and tuft. **f** mRNA expression of stem promoting markers including classic adult intestine stem cell markers, embryonic stem cell markers, intestinal reserve (+4) stem cell markers, and RNA regulators showing enriched expression in some differentiated cell types. Star annotation by gene name symbolizes significant differential mRNA expression in at least one cell type compared to stem (padj < 0.01). mRNA expression values are normalized counts and error bars are standard deviation. mRNA differential expression analysis was performed with the following biological replicate numbers: stem = 3, AbsPro = 3, SecPDG = 4, tuft = 5, Ent = 5, and EEC = 2.

Wnt signaling and its broader network of cross-talking signaling systems (e.g., Myc, Hippo, Egf, Kit) have a well-established role in maintaining the intestinal stem cell niche and allowing for differentiation of progenitor cells upon exit from that niche. Wnt transcription factors *Tcf7* and *Tcf7l2* are the predominant family members in stem cells, but *Tcf7l1* and *Tcf7l2* are even more highly expressed in progenitor and mature populations along with negative regulators, such as the Tle repressors (Fig. 7a, Supplementary Figs. 26a–d, 27a). Hippo mediator Yap1 is expressed ubiquitously but is highest in stem cells. Its binding partners (notably *Tead1* and *Tead3*) are broadly expressed, whereas the direct negative regulator *Insm1* is elevated in EECs (Fig. 7b, Supplementary Fig. 26e)^62^. Interestingly, EECs express very high levels of bone morphogenic ligand (*Bmp2*, Fig. 7b, Supplementary Fig. 26f). Bmp2 and Yap1 function in a well-characterized signaling circuit in multiple systems^63,64^, suggesting that EECs are likely to utilize the autocrine Bmp2–Yap1 signaling pathway. The transcription factor Myc is most highly expressed in stem cells, but its binding partner Max, which can heterodimerize with multiple different E-box factors, is broadly expressed with strong elevation in Ent (Fig. 7c). Binding partners of Max are strongly expressed in the various populations including direct repressors (Mondo Family, Mnt, Mga, Mad repressors; Fig. 7c). Expression of these transcriptional regulators and an array of negative regulators in most cryptal cell types implies that there is inherent capacity for gene regulation by their networks, suggesting that the absence of signal-activating ligands and the expression of direct inhibitors keeps these networks in a silent or quiescent state.

Kit and Egf signaling pathways are known to be critical for stem cell homeostasis^65,66^, yet their expression patterns suggest that there is potential for additional cross-talk signaling with the rare tuft and EEC cell types. Previous work has suggested that Kit (cKit; Cd117), the receptor for kit ligand (*Kitl;* Stem cell factor) that directs cell survival pathways in stem cell niches, is specific for Paneth cells in the small intestine and DCS/goblet cells in the colon^65^. Although we observed highly expressed *Kit* mRNA in SecPDG and tuft populations (Fig. 7d), our FACS protocol using Kit as a tuft cell sorting marker shows that at the protein level it is only detectable in tuft cells (Supplementary Fig. 1; at least a 5-fold increase in the cKit geometric mean and median in tuft compared with SecPDG). We also found that the ligand *Kitl* is most highly expressed in stem and AbsPro populations and to a lesser extent in the Ent population. Complementary expression patterns between the absorptive (Kit ligand) and secretory (Kit receptor) cell populations suggest that Kit could be an intra-cryptal signal from the absorptive lineage to tuft cells. This is mainly a soluble signal since the dominant spliced isoform of *Kitl* (inclusion of exon 6) is the  secretable isoform (Fig. 7d; inset). We observed a related pattern of Egf ligand expression in the colon, with the highest expression detected in SecPDG and tuft (Fig. 7e). Other Egf-related ligands are most highly expressed in enterocytes. Egf receptor mRNA (*Egfr*), and its negative regulators (*Lrig1*, *Cbl*, and *Ptpn6*; Fig. 7e, f, Supplementary Fig. 27b) are expressed broadly but receptor mRNA levels are highest in EEC and Ent. Interestingly, immunohistochemistry shows that in each crypt, Egfr is only evident in a few cells with morphologies indicative of EEC and tuft cells (Supplementary Fig. 28;^67^). Other Egf receptor family members, *Erbb2*, and *Erbb3*, are highly expressed in all cell types including stem cells (Fig. 7e).

Finally, the intestinal crypt is known for its impressive plasticity to rapidly regenerate stem cells at the base of wounded crypts. Multiple studies have shown that the epithelial cell populations, including Ent, tuft, EEC, and progenitor cells have the capacity to de-differentiate into stem cells and restore the niche^2–4^. Although the process of re-acquisition of stemness is not fully understood, our data indicate that colon epithelial populations continue to express mRNAs encoding stem cell regulators (Fig. 7f), including Lgr5 in tuft cells and embryonic stem cell markers in EECs (Fig. 7f, Supplementary Fig. 3b, c). Importantly, EECs and tuft cells also express intestinal reserve stem cell markers, most notably *Msi1*, *Msi2*, and *Prox1*. Furthermore, at least five known RNA regulators that promote pluripotency in embryonic stem cells (*Ddx6, Rbfox2, Son, Srsf2*) are robustly expressed in all colon crypt populations. These expression patterns show that subsets of known stemness regulators are a broadly shared feature of all intestinal crypt cell types.

## Discussion

**Fig. 8:**
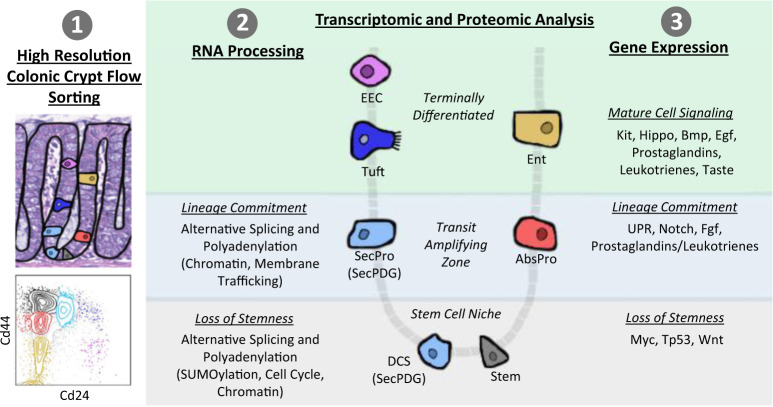
Summary of transcriptome and proteome changes in colon crypt homeostasis. Our findings encompass three main themes: (1) methodology—high-resolution colonic crypt flow sorting to isolate stem cells, SecPDG (secretory progenitors/deep crypt secretory cells), AbsPro (absorptive progenitors), tuft cells, enterocytes (Ent), and enteroendocrine cells (EEC), (2) RNA processing (splicing and polyadenylation) influences the transcriptome most during loss of stemness and lineage commitment, and (3) gene expression changes influencing lineage commitment and mature cell signaling.

This study presents a high-resolution cell sorting protocol for mouse colon crypt epithelia, an advance that permitted deep RNA-seq and proteomics analyses of multiple cell types including progenitor cells for absorptive and secretory lineages (Fig. 8). A key feature of our protocol was the elimination of protease treatments, which maximized biomarker sensitivity and cellular resolution and allowed us to clearly separate daughter–progenitor cells from parental stem cells. This advance enabled transcriptomics and proteomics profiling of the early changes occurring during loss-of-stemness and lineage commitment. Our analysis showed that before there are major changes in gene expression, changes in RNA processing (i) “re-configures” the stem cell transcriptome as stem cells lose stemness—altering splicing and polyadenylation patterns, (ii) likely influences cell fate choice or stabilization of lineage transitions, and (iii) that it does so through global changes in the regulatory networks that shape signal transduction and the proteome, including protein SUMOylation and epigenetic regulation. This suggests that the early stages of cellular differentiation involve a fundamental change in the activity, and/or stability of mRNA and their protein products rather than changes in mRNA levels. In addition to identifying altered RNA processing patterns, our analysis also identified new potentials for autocrine/paracrine signaling between different cell populations in the colon crypt.

Global analysis of gene expression in all six sorted cell populations enabled a more precise identification of stem cell markers (Fig. 2c), revealing that some commonly used stem cell markers are not exclusive to stem cells (e.g., Lgr5, Smoc2, Cd44; Supplementary Fig. 15a). We also identified protein markers such as Aquaporin 1, a transporter protein enriched in stem cells even though its mRNA is expressed in other cell types (Aqp-1; Supplementary Fig. 29). As stem cells differentiate, biomarkers of tissue identity emerge in progenitors and remain expressed in mature cells despite their disparate phenotypes and functions. In other words, we found that stem cells are not so much defined by what they express, but by what they do not express. Most notably, they are distinguished by unique patterns of alternative mRNA splicing and polyadenylation, patterns that dominate transcriptome changes as stem cells begin to differentiate.

The precise point that stem cells lose stemness is not clearly defined but is thought to happen soon after stem cell division as the newly produced progenitor (daughter) cell leaves the stem cell niche and enters the TAZ^6^. The shared changes in RNA processing in the AbsPro and SecPDG populations might therefore represent events that occur during these earliest steps of transition. Indeed, changes in splicing and polyadenylation were detected in regulators of Wnt, Notch, and other known regulators of intestinal stem cells. For example, alternative RNA splicing of delta-catenin mRNA removes an exon that encodes a Numb binding domain in progenitor populations, and it removes an exon for RNA binding domain in Split ends (Spen) mRNA in stem cells (Fig. 4d). Numb is a regulator of asymmetric cell divisions and a repressor of Notch signaling; Spen regulates early commitment choices of intestinal stem cells with activities that suppress Notch and activate Wnt^68^.

SUMOylation is the most significant ontology category associated with commonly processed mRNA targets (Fig. 4c, Supplementary Fig. 20). SUMO proteins are ubiquitin-like proteins that are covalently attached to proteins but unlike ubiquitination, SUMOylation influences the activity and/or localization of proteins rather than triggering degradation. Alternative RNA processing of mRNA encoding SUMOylation regulators suggests that this network may function differently in stem cells versus progenitor cells, and perhaps contributes to the earliest changes in transition between states (Supplementary Fig. 21b, c).

A comparison of gene expression patterns in the secretory and absorptive cell populations revealed new potential intra-cryptal signaling networks, most of which appear to target the secretory lineage. For example, we found that prostaglandin signaling is a potential feedback signal from tuft cells to secretory progenitors. Tuft cells can direct production of prostaglandin E2, whereas SecPDG progenitors express the PGE2 receptor *Ptger4* (Fig. 6d), an expression pattern that could explain why knockout of *Ptger4* in the intestine leads to the loss of secretory cells^69^. Another class of prostaglandin receptor, peroxisome proliferator-activated receptors alpha and gamma (*Ppara* and *Pparg*) are most highly expressed in the absorptive lineage, implying that prostaglandins have different roles in the two lineages (Supplement 25d)^70–72^. The overall expression pattern of prostaglandin genes suggests that tuft cells could provide prostaglandin precursors to all cryptal cell types for conversion and whole-cryptal production of PGE2 (*Ptges2, 3*), a potential form of “crowd-sourcing” of a signal known to be important for responding to wounding (Fig. 6d, Supplementary Fig. 25).

The activity of the Egf, Fgf, and Kit signaling systems are also enriched in the secretory lineage. Fgf receptors 2 and 3 (Fig. 6c) are most highly expressed in secretory cells and the Fgf target gene *Etv5* is most highly expressed in SecPDG and tuft (Fig. 6c). These patterns are consistent with knockout phenotypes in the intestine. For example, *Fgfr3*-knockout mice display enhanced proliferation in the TAZ^73^, and knockout of *Fgfr2c* in zebrafish leads to a loss of goblet and EEC^74^. UPR is most active in the secretory lineage (Fig. 6b), suggesting that like Egf, Fgf, and prostaglandins, this stress signal has a prominent role in commitment and differentiation along the secretory lineage and that the absorptive lineage can exert influences on these four signals. Enterocytes express prostaglandin uptake and degradation enzymes and therefore have potential to function as signal silencers, limiting the concentration and/or duration of signaling to the SecPDG population (Fig. 6d).

Our study contributes to understanding how multiple cryptal cell types can respond to damage via rapid de-differentiation for crypt repair and stem cell replacement^2–5^. We found that the gene expression patterns of known pluripotency and multi-potency regulators are expressed broadly in the crypt. Thus, these gene loci are not silenced and inactive, but open and expressed, and in some mature cell types expressed at high levels (Fig. 7c, f)^75^. Likewise, the loss of Wnt signaling during differentiation is not owing to a loss of expression of signaling pathway components. Although decreased Wnt signaling during differentiation is partly owing to decreased Wnt ligand availability outside the stem cell niche, our data also show that Wnt inhibitors are expressed in mature populations (Supplementary Fig. 26c)^76^. As Wnt signaling components continue to be expressed (Supplementary Fig. 27a), the pathway could be re-activated if ligands become available and/or inhibitor action is overcome. In addition, proposed biomarkers of reserve/quiescent stem cells are expressed broadly in all cell types and strongly expressed in EEC and the secretory lineage. Perhaps most striking is the broad expression pattern of RNA regulators that play key roles in pluripotency by specifying patterns of APA and splicing in embryonic stem cells. Given that RNA processing patterns change markedly in the transition between stem cells and progenitor cells, and then also resolve as mature cells differentiate, the repair of the stem cell niche in wounded crypts might depend on reactivation of these processing changes in wounding and de-differentiation.

In summary, the early emergence of splicing and polyadenylation changes during stem cell differentiation is a novel and unexplored process in the intestinal crypt. This process might not be limited to the intestine but may be a general feature of somatic stem cell differentiation. There are known mRNA processing changes during loss of pluripotency in mouse embryonic stem cells^29–38^, and changes in RNA processing have been identified in various disease states. However, very little is known regarding global changes in RNA processing during normal adult tissue homeostasis and especially during the earliest changes that occur during loss-of-stemness. The data sets and analyses presented here lay the groundwork for establishing an important role of mRNA processing as it relates to the rapid crypt dynamics and the de-differentiation potentials of mature intestinal cells.

## Methods

### Mouse colons

All mouse work was performed in accordance with NIH guidelines and was approved by the Institutional Animal Care and Use Committee (IACUC) of the University of California, Irvine, approval numbers AUP- 17-053. Male C57BL/6 N(NJ), obtained from the KOMP repository, mice aged 5–7 weeks were used unless otherwise noted (see Supplementary Fig. 14). Other mice used include Lgr5-EGFP-IRES-creERT2 mice (B6.129P2-*Lgr5*^*tm1(cre/ERT2)Cle*^/J, Stock Number 008875)^24^, agouti mice (129S1/SvImJ, Stock Number 002448), and NSG mice (NOD.Cg-*Prkdc*^*scid*^ *Il2rg*^*tm1Wj1/SzJ*^, Stock Number 005557), which were purchased from The Jackson Laboratory. FVB/NCrl mice (Strain Code 207) and BALB/cAnNCrl mice (Strain Code 028) were purchased from Charles River. A detailed step-by-step procedure is available through Nature Protocol Exchange^77^. In brief, mouse colons (cecum to rectum) were removed, flushed, and linearized. Tissue was dissociated at a slow rotation at 4 °C for 1 hr in a solution of 2 mM EDTA and 10 µM Rock inhibitor. Aggressive shaking of the tissue solution, filtering (using 100 µm followed by 40 µm filters), and centrifugation (500–1000 × *g* for 5–10 min at 4 °C depending on the step) were performed to isolate single cells. Data in the Supplementary Fig. 2 show crypt analysis, which included TrypLE (5 mL for 8 min; Life Technologies #12605010) dissociation step after the 100 µm filter step to ensure single cell suspension. A key feature of the sorting protocol is eliminating the use of any protease treatment (notably TrypLE) to preserve maximum cell surface levels of Cd44. The absence of protease action decreased cellular yield, but it also increased biomarker sensitivity and cellular resolution, effectively isolating daughter cells (secretory and absorptive progenitor populations) away from the parental stem cells.

### Flow cytometry antibody prep

Colon crypt single cell suspensions were DNAse treated for 5 min (Sigma-Aldrich #4716728001). Following a wash step, cells were incubated for 30 min in FACS buffer (phosphate buffered saline with 3% fetal bovine serum + 10 µM Rock inhibitor (Y-27632 AdipoGen Life Sciences from Fisher #501146540)) with the following pre-conjugated validated flow antibodies: CD45-BV510 (1:200, Clone 30-F11; BD Biosciences #563891), CD31-BV510 (1:200, Clone MEC 13.3; BD Biosciences #563089), CD326-eFluor450 (1:100, Clone G8.8; eBioscience #48-5791-82), CD44-PerCP-Cy5.5 (1:100, Clone IM7; Thermo Fisher #A26013), CD24-PECy7 (1:200, Clone M1/69; eBioscience #25-0242-82), and CD117-APC-Cy7 (1:100, Clone 2B8; Thermo Fisher #A15423). Following wash steps, cells were resuspended in FACS buffer and Live/Dead Aqua (Thermo Fisher # L34957). An alternative CD45-APC (1:200, Clone 30-F11; BD Biosciences #561018) antibody was used in the Supplementary Fig. 7f where specified.

### Flow sorting

Cells were bulk sorted on a BD FACS Aria Fusion using a 100 µm nozzle (20 PSI) at a flow rate of 2.0 with a maximum threshold of 5,000 events/sec. The sample chamber and collection tubes were kept at 4 °C. Following exclusion of debris and singlet/doublet discrimination, cells were gated as demonstrated in Supplementary Fig. 1. For RNA-seq, populations were sorted into TRIzol (Invitrogen #15-596-018) for downstream RNA isolation. For global proteome profiling, populations were sorted into PCR tubes containing 50 µL of 100 mM ammonium bicarbonate. At least 100 cells were sorted for each sample and tubes were promptly spun down and frozen until further processing. FACS plots and analysis was done using BD FACSDiva software.

### RNA preparation and RNA-seq

RNA was extracted from TRIzol samples using a Direct-zol RNA Micro-Prep kit (Zymo #11-330 M) and associated guidelines. Sorted samples of each cell type were pooled as needed at the start of RNA preparations to ensure a minimum of 2,500 cells per sample. RNA sample quality and concentration was evaluated using an Agilent Bioanalyzer on an RNA high sensitivity pico chip. RNA samples were then pooled as needed to allow 1 ng library preps with Clontech Low Input Pico Kit (Takara #634940). Following confirmation of library quality by Agilent Bioanalyzer DNA high sensitivity chip, a total of 22 samples were sequenced (biological replicate numbers stem = 3, AbsPro = 3, SecPDG = 4, tuft = 5, Ent = 5, EEC = 2). Samples were multiplexed and sequencing was performed with 100 bp paired-end run on Illumina HiSeq 4000.

### RNA-seq data analysis and visualization

Paired-end sequencing reads were trimmed of adapter sequences and analyzed for quality using Fastqc (version 0.11.7). Data were aligned to the mouse genome (UCSC mm10 from Illumina iGenome) using STAR (version 2.5.2a), converted to bam files and merged (samtools 1.3) and read counts were generated using HTSeq (version 0.6.1p1, with enthought_python version 7.3.2; option -s no). Differential gene expression analysis was done in RStudio (version 1.0.153) with R (version 3.6.1) using default setting of the DESeq2 pipeline for statistical analysis (version 1.16.1; with cooksCutoff = FALSE option)^78^. Gene expression significance was determined by DESeq2 Wald *P* value test with a padj < 0.01 with a minimum mean of 50 normalized counts. Heatmaps and PCA plot were generated in RStudio (version 1.0.153) with R (version 3.6.1) using pheatmap (with default scale settings) and plotPCA, respectively, of r-log-transformed (regularized log) DESeq2 data. r-log-transformation is a robust way to transform the count data, used in differential gene expression analysis, to a log2 scale in a way which minimizes differences between samples and normalizes with respect to library size, it is also a standard function for downstream analysis such as clustering or linear discriminant analysis. Bar graphs of gene expression data were generated in GraphPad Prism (version 6.01) with normalized read counts (output of DESeq2) and error bars defining standard deviation. Supplementary Data #1 contains processed global mRNA gene expression data. Raw fastq files along with processed data (counts files) are available for download on GEO (GSE143915).

### Splicing and polyadenylation analysis

Merged bam files were sorted and indexed (samtools 1.3) for downstream analysis. Alternative splicing was investigated using rMATS Turbo (rMATS.4.0.1) with STAR 2.5.2a, Samtool 1.3, and enthought_python 7.3.2 comparing two cell types at a time using UCSC mm10 gtf. MAJIQ (v1.1) was also run for alternative splicing with anaconda 3–2.0.1 and recommended mm10 ensembl gff3 reference with type = strand-specific followed by VIOLA for visualization. DaPars (v0.9.1) was used for alternative polyadenylation analysis with recommended mm10 UCSC reference files and python 2.7.15, bedtools 2.25.0, R 3.4.1, and the following settings (Num_least_in_group1 = 1, Num_least_in_group2 = 1, Coverage_cutoff = 30, FDR_cutoff = 0.05, PDUI_cutoff = 0.15, Fold_change_cutoff = 0.32). rMATS significance was defined in three different levels of significance: FDR < 0.05, FDR < 0.01, FDR < 0.01 with ±25% dpsi. Similarly, DaPars significance was defined in three different levels of significance: FDR < 0.05, FDR < 0.01, FDR < 0.01 with ±25% PDUI. Alternative processing gene lists are provided in Supplementary Data #3 and rMATS and DaPars output files for cell types compared to stem are available for download on GEO (GSE143915).

### Gene ontology and enrichment analysis

Gene ontology and gene enrichment analysis of mRNA-seq data were performed on specified gene lists using Panther^79^ (http://pantherdb.org/) and Enrichr^80,81^ (https://amp.pharm.mssm.edu/Enrichr/).

### Cell lysis and trypsin digestion for proteomic analysis

Prior to sample processing PCR tubes were centrifugated at 1000 × *g* for 10 min at 4 °C to keep the cells at the bottom of the tube to avoid potential cell loss. In all, 2 µL of 0.1% n-Dodecyl β-D-maltoside in 25 mM ammonium bicarbonate was added to each PCR tube with gentle shaking. Intact cells were lysed using sonication five times at 1-min intervals over ice and then centrifuged for 3 min at 3000 × *g*. Samples were then incubated on a thermocycler for denaturation at 75 °C for 1 h. In all, 1 µL and 2 µL of 10 ng/µL trypsin (Promega) in 25 mM ammonium bicarbonate was added to the PCR tubes at a total amount of 10 ng for <1000 cells and 20 ng for >1000 cells. Samples were digested for overnight (~16 h) at 37 °C with gentle sharking at ~500 × *g*. After digestion, 2 µL of 5% formic acid was added to the tube to stop enzyme reaction. The final sample volume was reduced down to ~20 μL using SpeedVac and the sample PCR tube was inserted into the Liquid chromatography vial for direct liquid chromatography–mass spectrometry (LC-MS) analysis. The processed samples were either analyzed directly or stored at −20 °C for later LC-MS analysis.

### LC-MS/MS analysis

The cell subpopulation digests were analyzed using a commonly available Q Exactive Plus Orbitrap MS (Thermo Scientific, San Jose, CA). The standard LC system consisted of a PAL autosampler (CTC ANALYTICS AG, Zwingen, Switzerland), two Cheminert six-port injection valves (Valco Instruments, Houston, USA), a binary nanoUPLC pump (Dionex UltiMate NCP-3200RS, Thermo Scientific), and an HPLC sample loading pump (1200 Series, Agilent, Santa Clara, USA). Both SPE precolumn (150 µm i.d., 4 cm length) and LC column (50 µm i.d., 70 cm Self-Pack PicoFrit column, New Objective, Woburn, USA) were slurry-packed with 3 µm C18 packing material (300-Å pore size) (Phenomenex, Terrence, USA). Sample was fully injected into a 20 µL loop and loaded onto the SPE column using buffer A (0.1% formic acid in water) at a flow rate of 5 µL/min for 20 min. The concentrated sample was then separated at a flow rate of 150 nL/min and a 75 min gradient of 8–35% buffer B (0.1% formic acid in acetonitrile). The LC column was washed using 80% buffer B for 10 min and equilibrated using 2% buffer B for 20 min. Q Exactive Plus Orbitrap MS (Thermo Scientific) was used to analyze the separated peptides. A 2.2 kV high voltage was applied at the ionization source to generate electrospray and ionize peptides. The ion transfer capillary was heated to 250 °C to desolvate droplets. The data-dependent acquisition mode was employed to automatically trigger the precursor scan and the MS/MS scans. Precursors were scanned at a resolution of 35,000, an AGC target of 3 × 10^6^, a maximum ion trap time of 100 ms. Top-10 precursors were isolated with an isolation window of 2, an AGC target of 2 × 10^5^, a maximum ion injection time of 250 ms (for >300 cells, the AGC target of 2 × 10^5^ and 100 ms ion injection time was used), and then fragmented by high energy collision with an energy level of 32%. A dynamic exclusion of 30 s was used to minimize repeated sequencing. MS/MS spectra were scanned at a resolution of 17,500.

### Proteomics data analysis

The freely available open-source MaxQuant software was used for protein identification and quantification. The MS raw files were processed with MaxQuant (Version 1.5.1.11)^82,83^ and MS/MS spectra were searched by Andromeda search engine against the against mouse UniProt database (fasta file dated 12 April 2017) (with the following parameters: tryptic peptides with 0–2 missed cleavage sites; 10 ppm of parent ion tolerance; 0.6 Da of fragment ion mass tolerance; variable modifications (methionine oxidation). Search results were processed with MaxQuant and filtered with a false discovery rate ≤1% at both protein and peptide levels. For label-free quantification, the match between runs (MBR) function was activated with a matching window of 0.4 min and the alignment window of 20 min. The quantitation results were extracted from MaxQuant outputs based on at least two valid values in one sample type by using Peruses (Version 1.5.8.3)^84^. Supplementary Data #2 contains processed global protein expression data.

### Protein staining

All protein staining images are from the Human Protein Atlas and readily available at http://www.proteinatlas.org. Tissue in these images are from the intestine and labeled with the specific location including duodenum, small intestine, colon, or rectum^85,86^.

### Statistics and reproducibility

More than 200 mice were used to optimize and validate the flow sorting procedure and perform mRNA sequencing and proteomics. For proteomics three biological replicate were collected for each cell type, each biological replicate is treated as one sample during data analysis. These biological replicates are from independent mice and independent flow sorts. For mRNA-sequencing additional mice had to be used and pooled in order to isolate enough cells for sequencing, particularly for rarer cell types. In total we sequenced the following number of biological replicates (aka samples) per cell type stem = 3, AbsPro = 3, SecPDG = 4, tuft = 5, Ent = 5, EEC = 2. These biological replicates are from independent mice (sometimes sets of pooled mice) and independent flow sorts. Pooling of independent sorts was done as needed to ensure >2,500 cells for RNA preparation as described in the “RNA Preparation and RNA-seq” method section. The number of independent mice for each of the biological replicates per cell type is as follows: stem = 1,1,1; AbsPro = 1,1,1; SecPDG = 2,2,4,4; tuft = 4,4,4,4,4; Ent = 5,5,6,5,5; EEC = 8,10.

No data exclusion were performed and no randomization or blinding methods were used in data analysis. Gene expression and mRNA processing bioinformatic packages (DESeq2, rMATS, and DaPars) were used for statistical analysis as specified in the appropriate methods section. GraphPad Prism (version 6.01) was used for additional analysis including: Fig. 6b—Xbp1 splicing (unpaired two-sided *t* test); standard deviation quantitation for mRNA expression graphs (Figs. 6, 7 and throughout the Supplemental material); and Supplemental Fig. 12—linear best fit lines and *R*^2^ values.

### Reporting summary

Further information on research design is available in the Nature Research Reporting Summary linked to this article.

## Supplementary information

Supplementary Information

Supplementary Data 1

Supplementary Data 2

Supplementary Data 3

Supplementary Data 4

Description of Additional Supplementary Files

Reporting Summary

Peer Review File

## Data Availability

Raw sequencing data (fastq) and processed data (counts files, rMATS, DaPars, and MAJIQ data) are available for download on GEO (GSE143915). The proteomics raw data sets and identified proteins groups lists generated from Maxqaunt have been deposited in Japan ProteOme STandard Repository^87^ (jPOST; https://repository.jpostdb.org/). The accession number is JPST000853 for jPOST and PXD019351 for ProteomeXchange. Supplementary Files accompanying this manuscript include: Supplementary Data #1—Global mRNA Gene Expression; Supplementary Data #2—Global Protein Expression; Supplementary Data #3—Marker Gene Lists (mRNA); and Supplementary Data #4—List of Alternatively APA + Spliced genes.
