# Publisher Correction: Transcriptomic and proteomic signatures of stemness and differentiation in the colon crypt

**DOI:** 10.1038/s42003-020-01237-0

**Published:** 2020-09-03

**Authors:** Amber N. Habowski, Jessica L. Flesher, Jennifer M. Bates, Chia-Feng Tsai, Kendall Martin, Rui Zhao, Anand K. Ganesan, Robert A. Edwards, Tujin Shi, H. Steven Wiley, Yongsheng Shi, Klemens J. Hertel, Marian L. Waterman

**Affiliations:** 1 Department of Microbiology and Molecular Genetics, University of California Irvine, Irvine, CA 92697 USA; 2 Department of Biological Chemistry, University of California Irvine, Irvine, CA 92697 USA; 3 Institute for Immunology, University of California Irvine, Irvine, CA 92697 USA; 4 Biological Sciences Division, Pacific Northwest National Laboratory, Richland, WA 99354 USA; 5 Environmental Molecular Sciences Laboratory, Pacific Northwest National Laboratory, Richland, WA 99354 USA; 6 Department of Dermatology, University of California Irvine, Irvine, CA 92697 USA; 7 Department of Pathology and Laboratory Medicine, University of California Irvine, Irvine, CA 92697 USA

**Keywords:** Intestinal stem cells, Colon, Proteomics, Transcriptomics, Flow cytometry

Correction to: *Communications Biology* 10.1038/s42003-020-01181-z, published online 19 August 2020.

In the original version of the published Article, the URL given in reference 77 for the associated protocol on the Protocol Exchange was incorrect. The error has been corrected in the HTML and PDF versions of the Article.

