# Supplementary material for: Transcriptomic and proteomic signatures of stemness and differentiation in the colon crypt
Source: Commun Biol. 2020 Aug 19;3:453. doi: 10.1038/s42003-020-01181-z (PMC7438495; doi:10.1038/s42003-020-01181-z)
Supplement: Supplementary file 1 — Supplementary Information [file 42003_2020_1181_MOESM1_ESM.pdf]

SUPPLEMENTARY FIGURE 1

1. Select single, live cells based on forward/side scatter.

2. Dump Cd45+/Cd31+/Dead cells

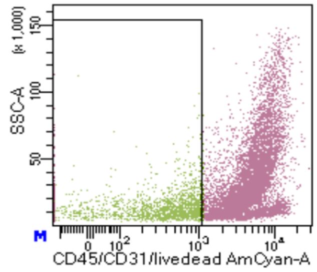

3. Select Epcam+

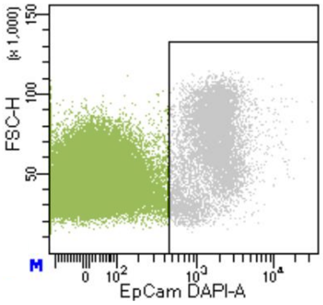

4. Gate two populations in Epcam+: (1) cKit-/Cd44low/- and (2) cKit-/Cd44+

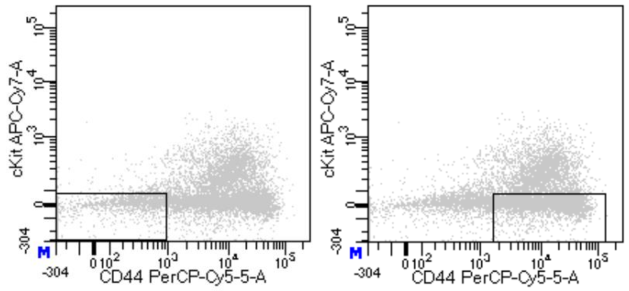

5. cKit-/Cd44low/- is gated on Cd24 = Cd24- (**ENT**) and Cd24+ (**EEC**)

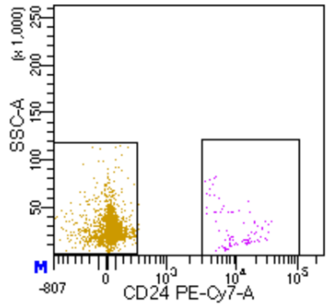

5. cKit-/Cd44+ is gated on Cd44/Cd24 = Cd44highest(**STEM**) and Cd44med/hi (**ABSPRO**)

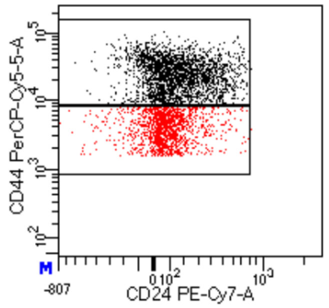

6. Epcam+ gated on cKit+/Cd24+ (**TUFT**)

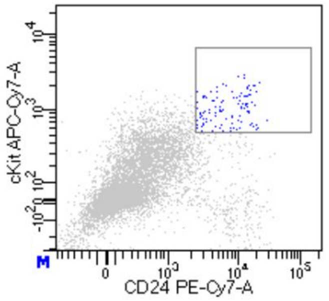

7. Epcam+ gated on Cd44hi/Cd24med (**SECPDG**)

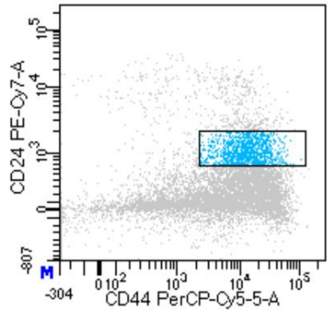

8. Resulting Populations

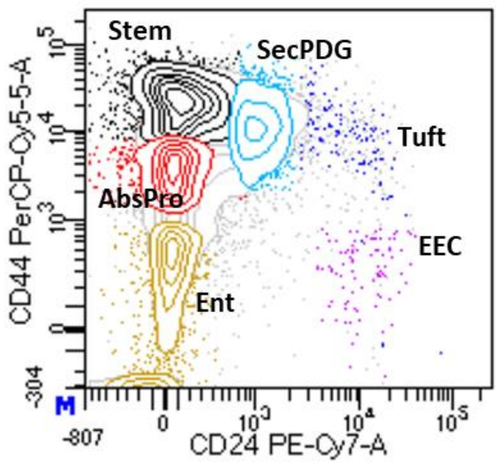

Relative Abundance of Cell Populations

	**Average % of Epcam+**	**Standard Deviation**
Stem	22.1%	3.9%
AbsPro	15.6%	2.5%
SecPDG	9.0%	4.8%
Ent	19.7%	5.2%
Tuft	0.8%	0.5%
EEC	0.7%	0.4%

n = 10 independent sorts

Supplementary Figure 1: FACS gating strategies that define six colon crypt cell populations. As a first step, standard gating is performed to select single, live cells based on forward and side scatter (Step 1). A dump channel then removes dead cells along with immune cells (Cd45+) and endothelial cells (Cd31+) (Step 2). Epcam+ cells (Step 3) are then gated using Cd44, Cd24, and cKit to isolate six distinct populations (Steps 4-7). The resulting populations are enterocytes (Ent), enteroendocrine (EEC; predominantly enterochromaffin cells), stem cells, absorptive progenitors (AbsPro), tuft, and SecPDG (a mixed population of secretory progenitors, deep crypt secretory cells, with a minor contribution from goblet cells) (Step 8). Relative average abundance and standard deviation of these populations in n=11 independent sorts are shown as a percentage of Epcam+ cells. Note: Some Epcam+ cells are not clearly gated into one of these cell populations and thus the sum of the six populations does not add to 100%.

SUPPLEMENTARY FIGURE 2

a Dissociation with TrypLE

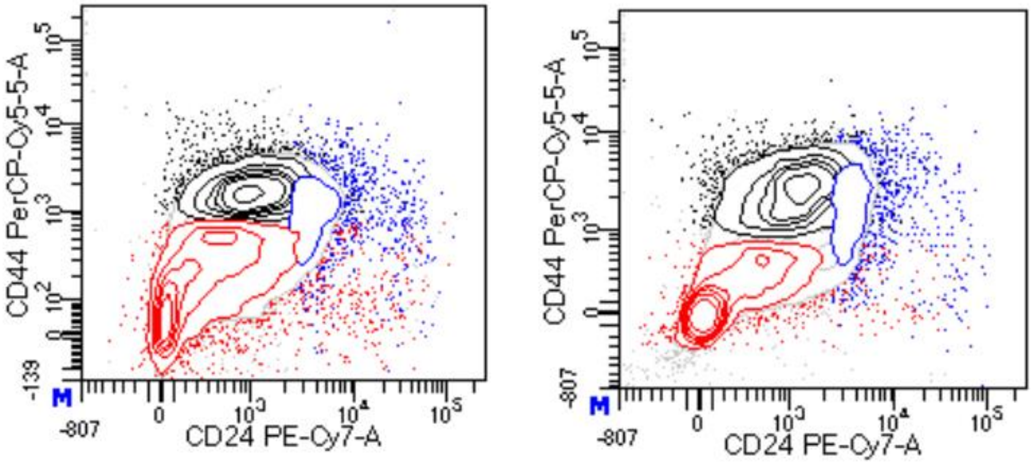

b Dissociation with **NO** TrypLE (EDTA only)

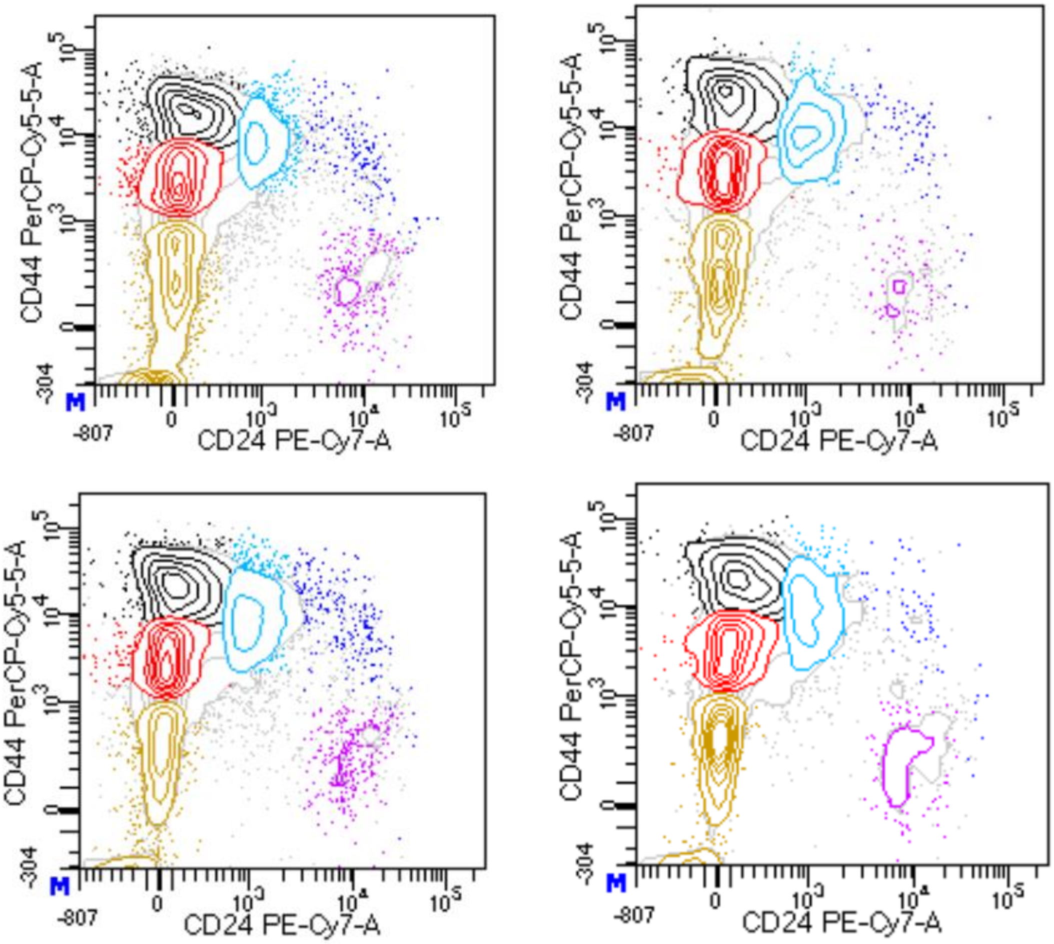

Supplementary Figure 2: FACS plots of colon crypts dissociated with or without TrypLE protease treatment. **a** When the TrypLE cocktail is used during intestine dissociation, FACS detects decreased Cd44 surface expression and the plots show a compressed population resolution compared to **b** no TrypLE. Each plot is from one mouse and is a representative image.

SUPPLEMENTARY FIGURE 3

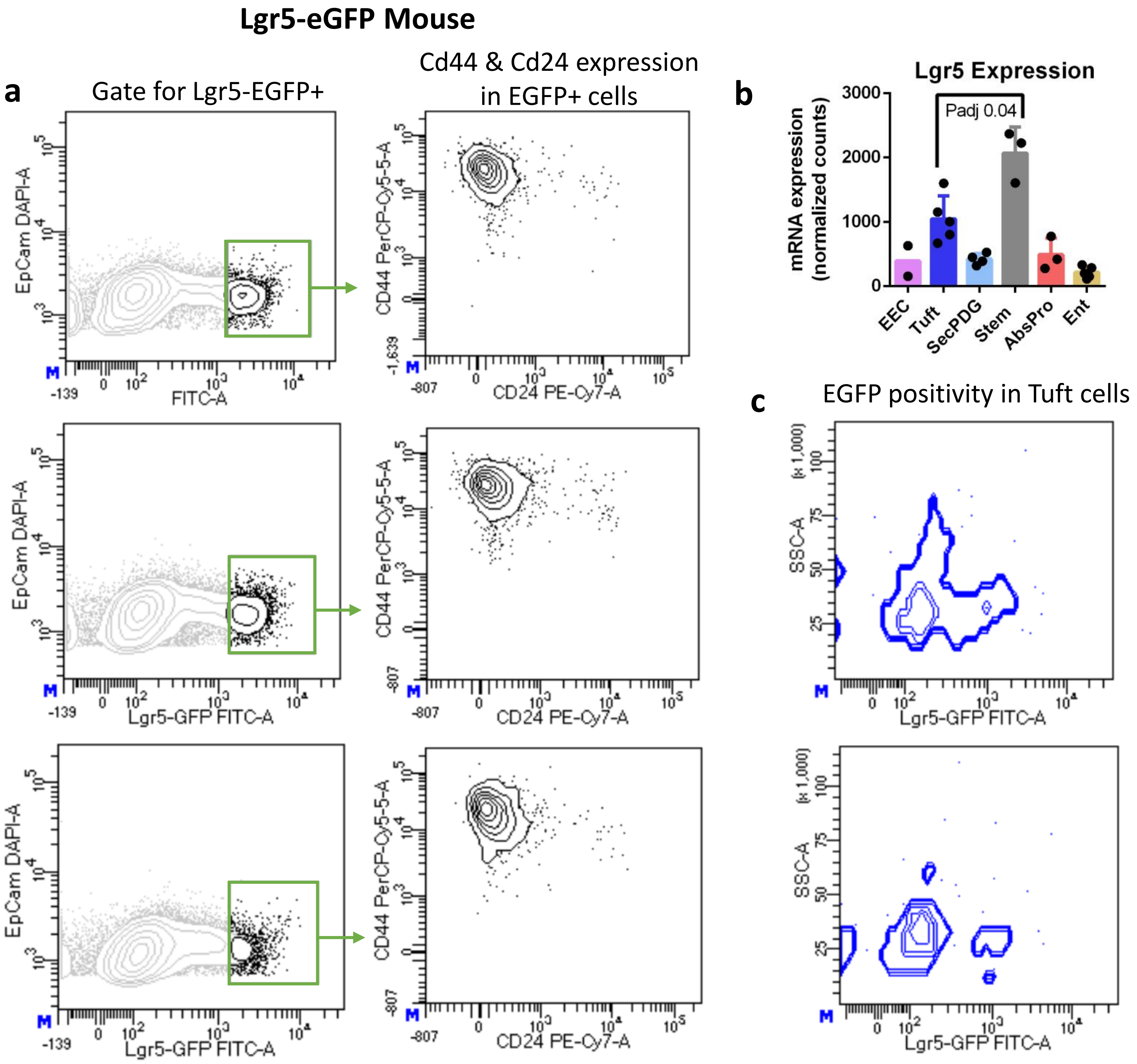

Supplementary Figure 3: Validation of sorted stem cell population with Lgr5-EGFP-IRES-creERT2 mice. **a** EGFP expression, used for labeling intestinal crypt stem cells via Lgr5-locus-directed transcription, is detected by FITC fluorescence and gated for +/high population. These cells are then displayed on a FACS plot with Cd44 and Cd24. The complete correlation between the GFP^{hi} stem cells and the Cd44^{hi}/Cd24^{lo} signature demonstrates a direct overlap of the two populations and confirms that Cd44^{hi}/Cd24^{lo} cells are colon crypt stem cells. n=3 independent mice/sorts. **b** mRNA expression of *Lgr5* shows the highest expression in the stem cell population, followed by only 2-fold lower in tuft cells and then 4-fold lower expression in AbsPro and SecPDG populations. mRNA differential expression analysis was performed with the following biological replicate numbers: stem=3, AbsPro=3, SecPDG=4, tuft=5, Ent=5, and EEC=2. **c** Lgr5-EGFP (FITC) expression in tuft cells reveals positivity confirming that tuft cells express Lgr5 at detectable levels.

SUPPLEMENTARY FIGURE 4

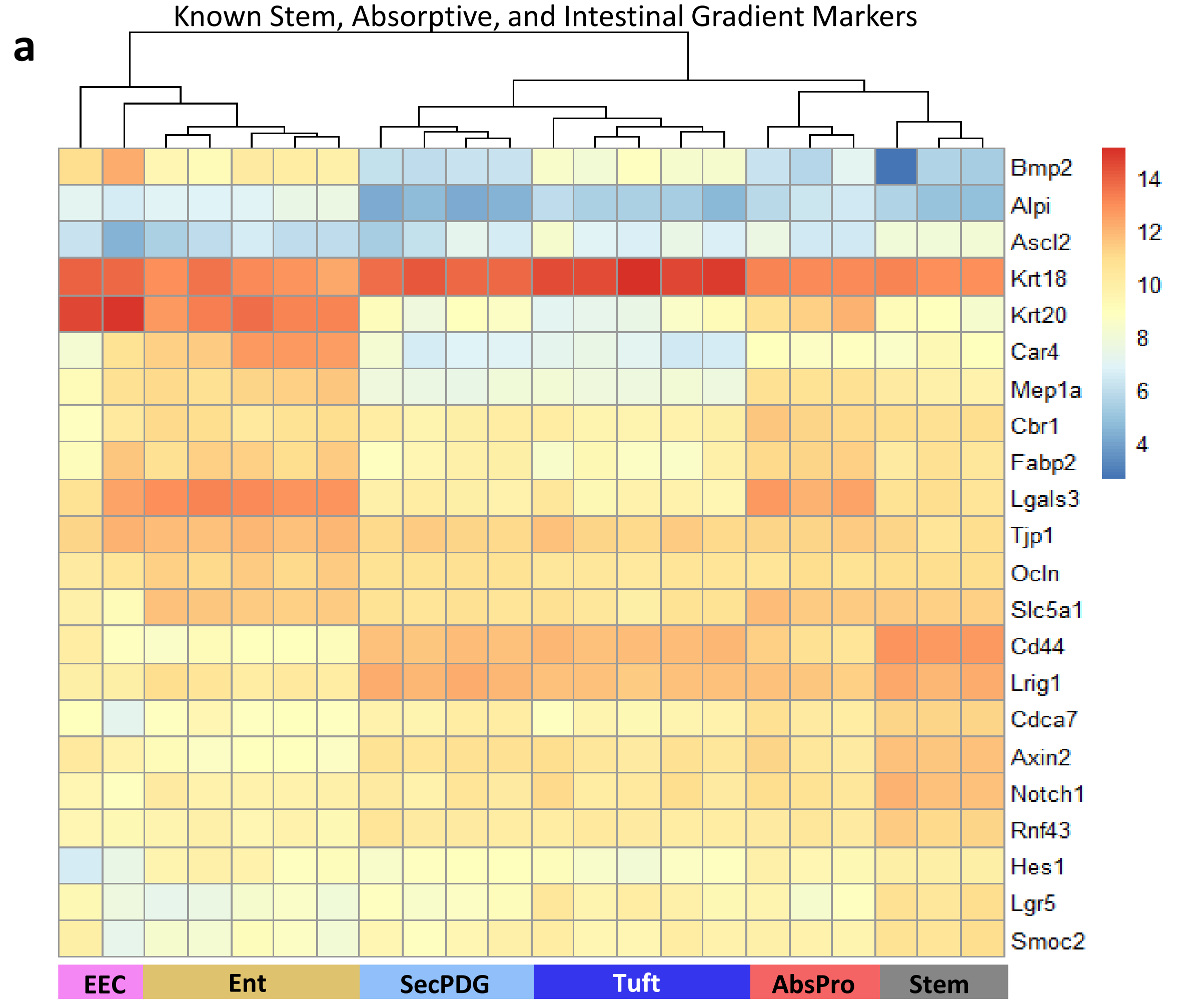

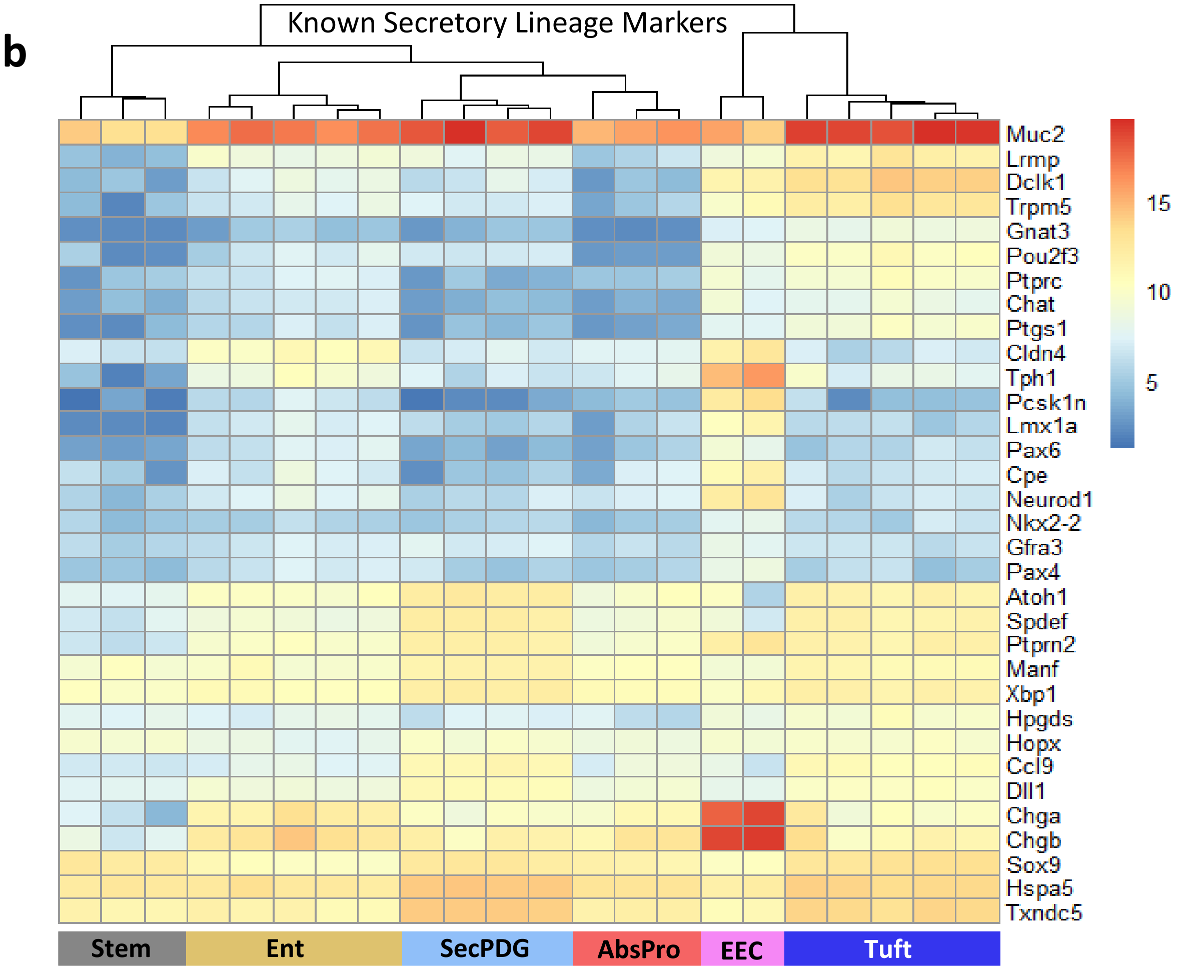

Supplementary Figure 4: Expression of known intestinal crypt markers. **a** Unsupervised clustering of known stem, absorptive lineage, and differentiation markers distinguishes cell types and confirms population identities. **b** Unsupervised clustering of known secretory lineage markers distinguishes secretory cell types, highlighting tuft markers (such as *Dclk1*) and high expression of *Chga/b* (EECs) and *Muc2*.

SUPPLEMENTARY FIGURE 5

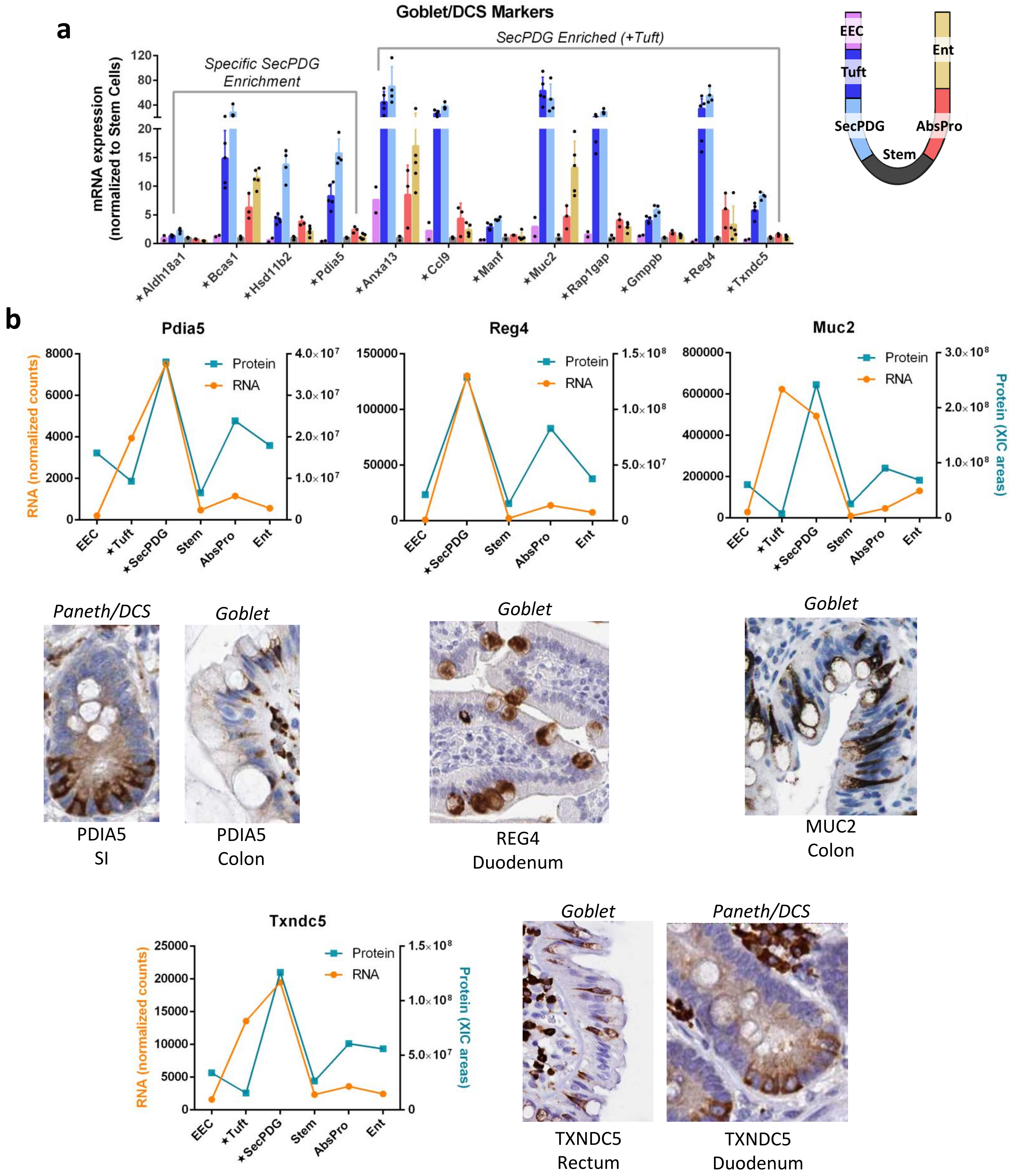

SUPPLEMENTARY FIGURE 5 continued

c SecPDG Markers enriched in Goblet Cells

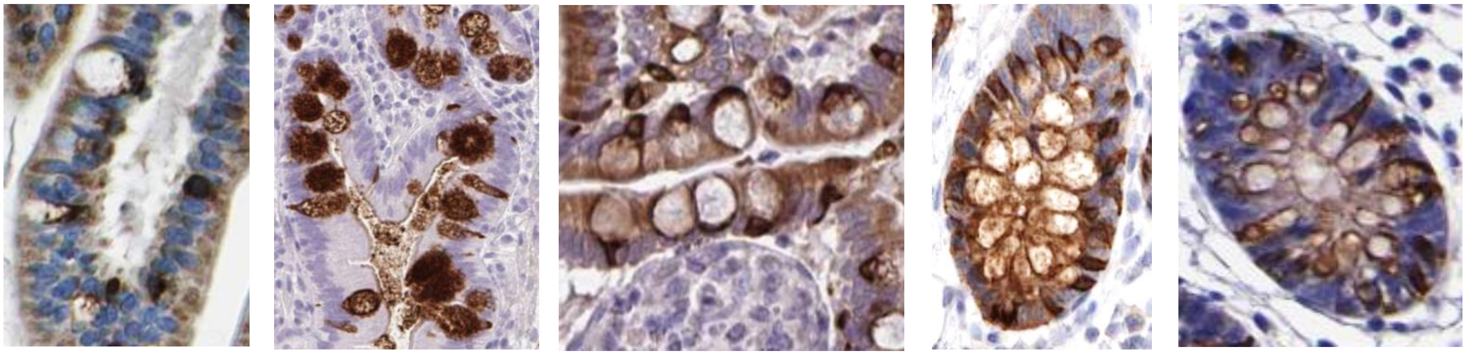

ALDH18A1 Colon | BCAS1 SI | HSD11B2 SI | ANXA13 Rectum | RAP1GAP Colon

d SecPDG Markers enriched Paneth Cells = Deep Crypt Secretory Cells

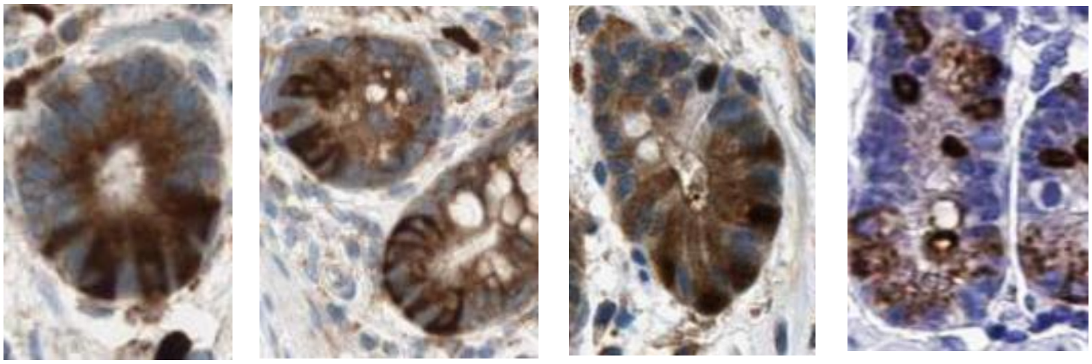

MANF Colon | MANF SI | GMPPB Colon | RAP1GAP Duodenum

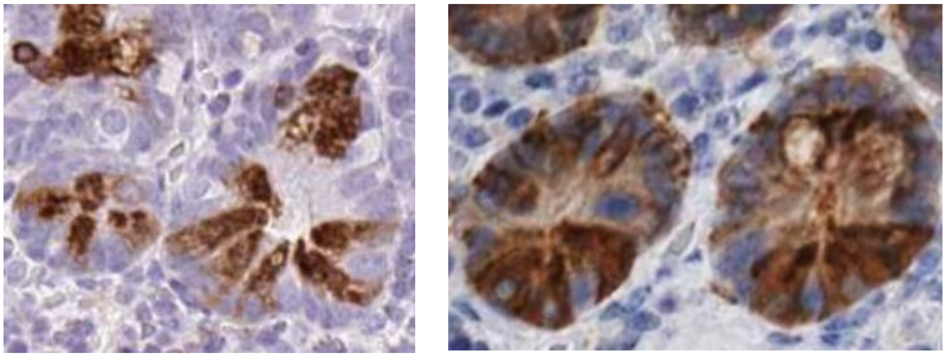

BCAS1 SI | ANXA13 Duodenum

Supplementary Figure 5: SecPDG marker genes label both goblet and deep crypt secretory cells (DCS). **a** mRNA expression of marker genes enriched selectively in SecPDG or in both SecPDG and tuft populations. Star annotation by gene name symbolizes significant differential mRNA expression in at least one cell type compared to stem (padj < 0.01). mRNA differential expression analysis was performed with the following biological replicate numbers: stem=3, AbsPro=3, SecPDG=4, tuft=5, Ent=5, and EEC=2. **b** Quantitative protein expression of marker genes Pdia5, Reg4, Muc2, and Txndc5 was detected with MS. Line graphs show the abundance of each protein compared to its mRNA expression across different cell types. Protein staining of these marker genes is evident in Paneth/DCS and Goblet cells. Star annotation by cell type symbolizes significant differential mRNA expression compared to stem (padj < 0.01). Staining of additional SecPDG markers in human tissues highlights **c** goblet cells, and **d** DCS/paneth cells (images from Human Protein Atlas). Some markers are expressed in both secretory progenitors, goblet, and DCS/paneth cell populations, whereas other markers are only expressed in specific subset populations. For example, REG4 and ALDH18A1 appear to be highly expressed in goblet cells along the crypt but not in DCS cells at the base of the crypt. MANF and GMPPB protein levels are high in DCS/paneth, but not goblet cells. PDIA5 antibody detects strong expression in paneth cells in the small intestine, but the same antibody does not clearly distinguish DCS in colon crypt (however it did mark colonic mature goblet cells).

SUPPLEMENTARY FIGURE 6

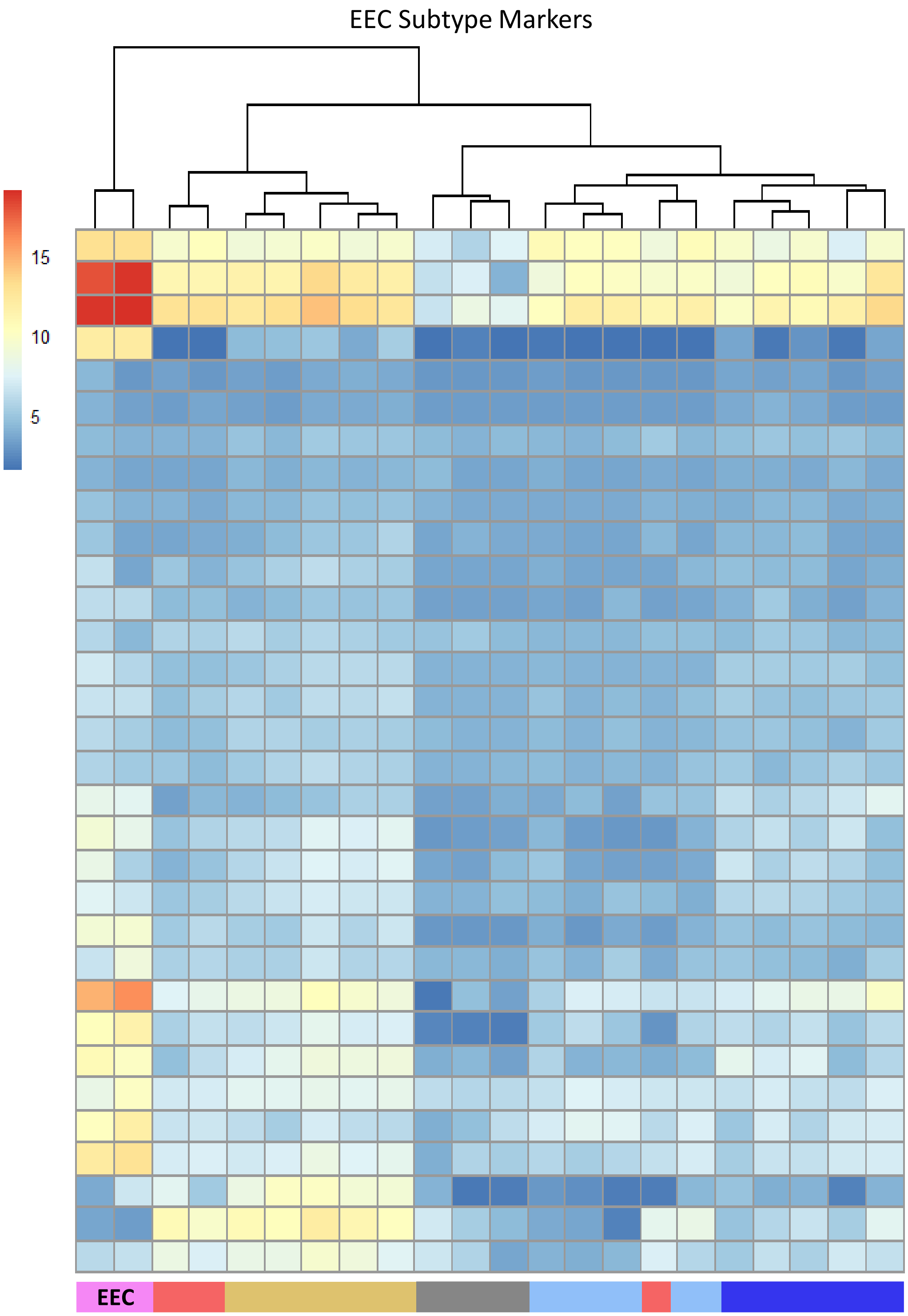

Gene	EEC Subtype
Sct	L, Ecm
Chga	Ecm
Chgb	Ecm
Iapp	
Agtr1a	L
Gal	
Ghrl	X
Ppy	L
Arx	X, I, L, K
Gip	K
Cck	L
Olfr558	Ecm
Insl5	L
Bdnf	
Isl1	N, L, K, D, I, X
Hhex	D
Scgn	
Sst	D
Pax6	L, K
Adgrd1	
Avpr1b	L
Olfr78	Ecm
Tac1	Ecm
Tph1	Ecm
Lmx1a	Progenitor
Piezo2	Ecm
Gch1	
Insm1	L
Neurod1	Progenitor
Nts	L
Gcg	L
Pyy	L

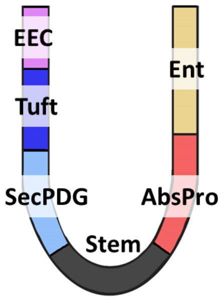

Supplementary Figure 6: Expression of known markers detects different subtypes of enteroendocrine cells (EEC). Unsupervised clustering of marker genes for EEC subtypes including L, X, I, K, N, D – cells, along with Ecm (enterochromaffin) and progenitors [1]. The sorted and sequenced EEC population presented here is predominantly Ecm, demarcated by the high expression of *Chga*, *Chgb*, and *Tph1*. Some L-cell markers, *Nts*, *Gcg*, and *Pyy*, are weakly expressed in Ent (and *Gcg* also in AbsPro). This suggests that rare L-cells might be pooled with Ent and additionally that Cd24 might be a distinguishing marker between different subtypes of EECs (with Ecm being Cd24 high).

SUPPLEMENTARY FIGURE 7

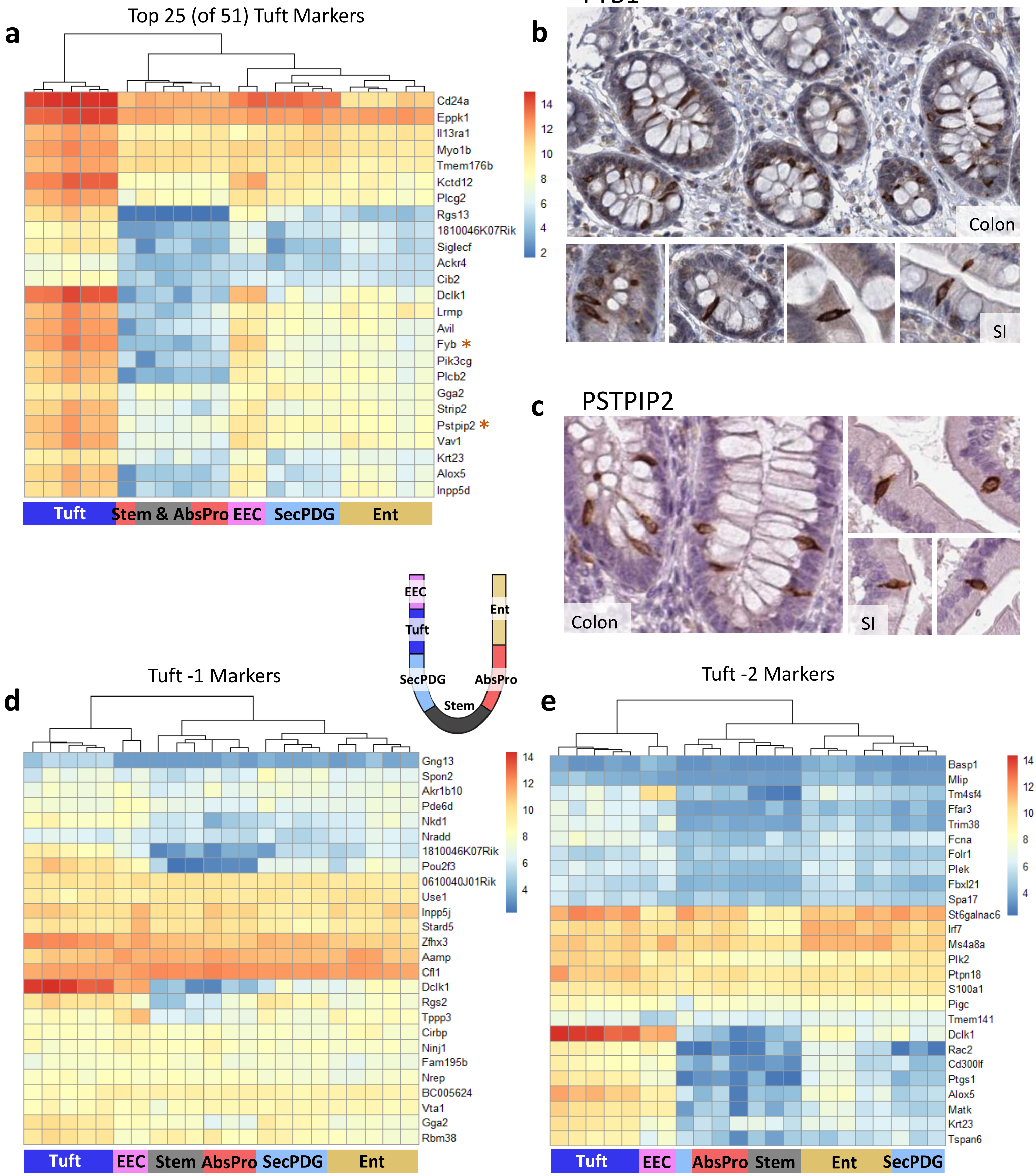

SUPPLEMENTARY FIGURE 7 continued

f

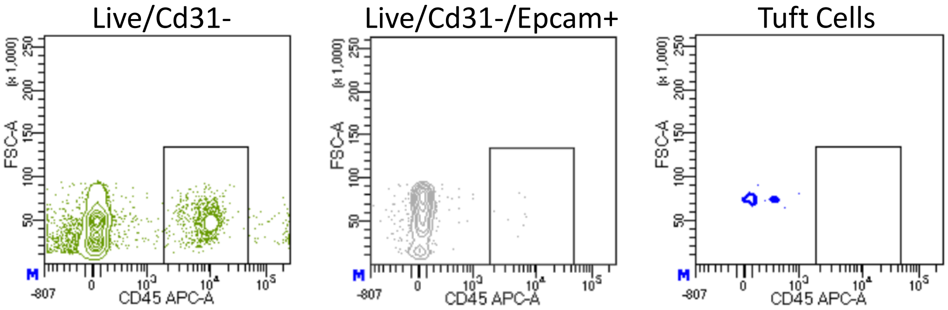

g

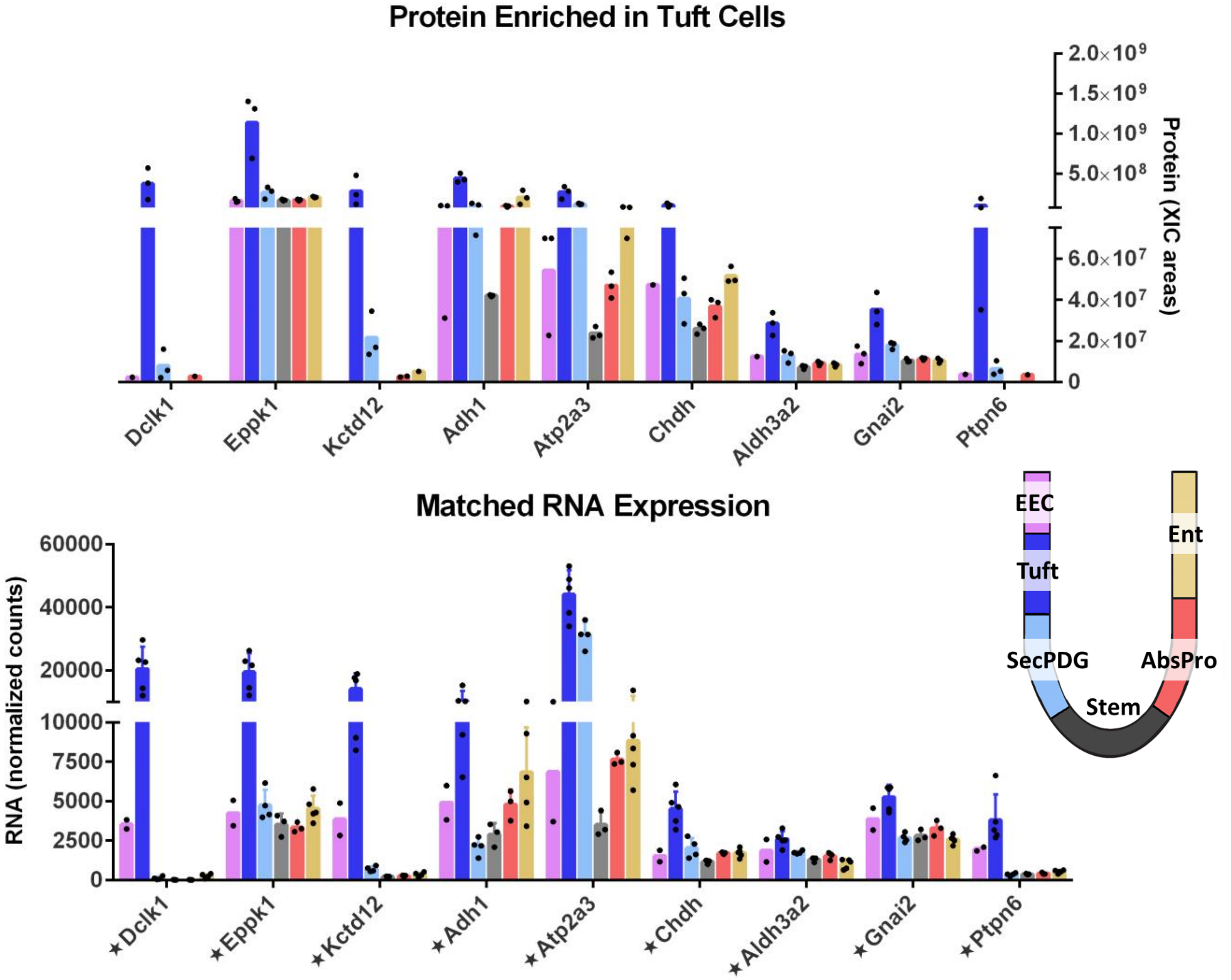

Supplementary Figure 7: Markers enriched in tuft cells. There were n=51 genes statistically enriched in mRNA expression in tuft cells compared to the five other isolated colon crypt cell types (padj < 0.01 + minimum mean 50 counts). **a** Unsupervised clustering of the top 25 genes confirms their differential expression in tuft cells versus non-tuft cells. Protein staining is shown for genes with orange asterisk. Human protein atlas images confirm specificity with unique staining of tuft cells for the following markers **b** FYB1 and **c** PSTPIP2 staining shows positivity in cells with cells matching tuft morphologies. Haber *et al* identified two populations of Dclk1+ tuft cells in the small intestine, with the Tuft-1 populations denoted as Cd45- and the Tuft-2 population denoted as Cd45+ [2]. Unsupervised clustering of **d** Tuft-1 and **e** Tuft-2 marker genes as determined by Haber *et al* shows that despite Cd45+ cells being dumped in our sorting procedure (Cd45 is a marker for immune cells), we observe high expression of Tuft-2 markers (*Rac2, Cd300lf, Ptgs1, Alox5, Matk, Krt23,* and *Tspan6* for example) in our sorted Tuft population [2]. **f** Additionally, when we removed Cd45 from the dump channel we did not observe any Cd45+ tuft cells. Live/Cd31- cells show a Cd45+ population that disappears once Epcam+ cells are sorted. We conclude that colonic tuft cells are not expressing Cd45+ at detectable levels under homeostatic conditions and therefore it is not a marker for this population. **g** Using the MS proteomics data we identified proteins enriched in tuft cells which included known marker genes such at Dclk1. Data is displayed in two graphs: protein expression and mRNA expression of the same genes. Star annotation by gene name symbolizes significant differential mRNA expression in at least one cell type compared to stem (padj < 0.01) and error bars are standard deviation. For proteomics there are n=3 independent biological replicates for each cell type; for mRNA differential expression analysis the following biological replicate numbers were used: stem=3, AbsPro=3, SecPDG=4, tuft=5, Ent=5, and EEC=2.

SUPPLEMENTARY FIGURE 8

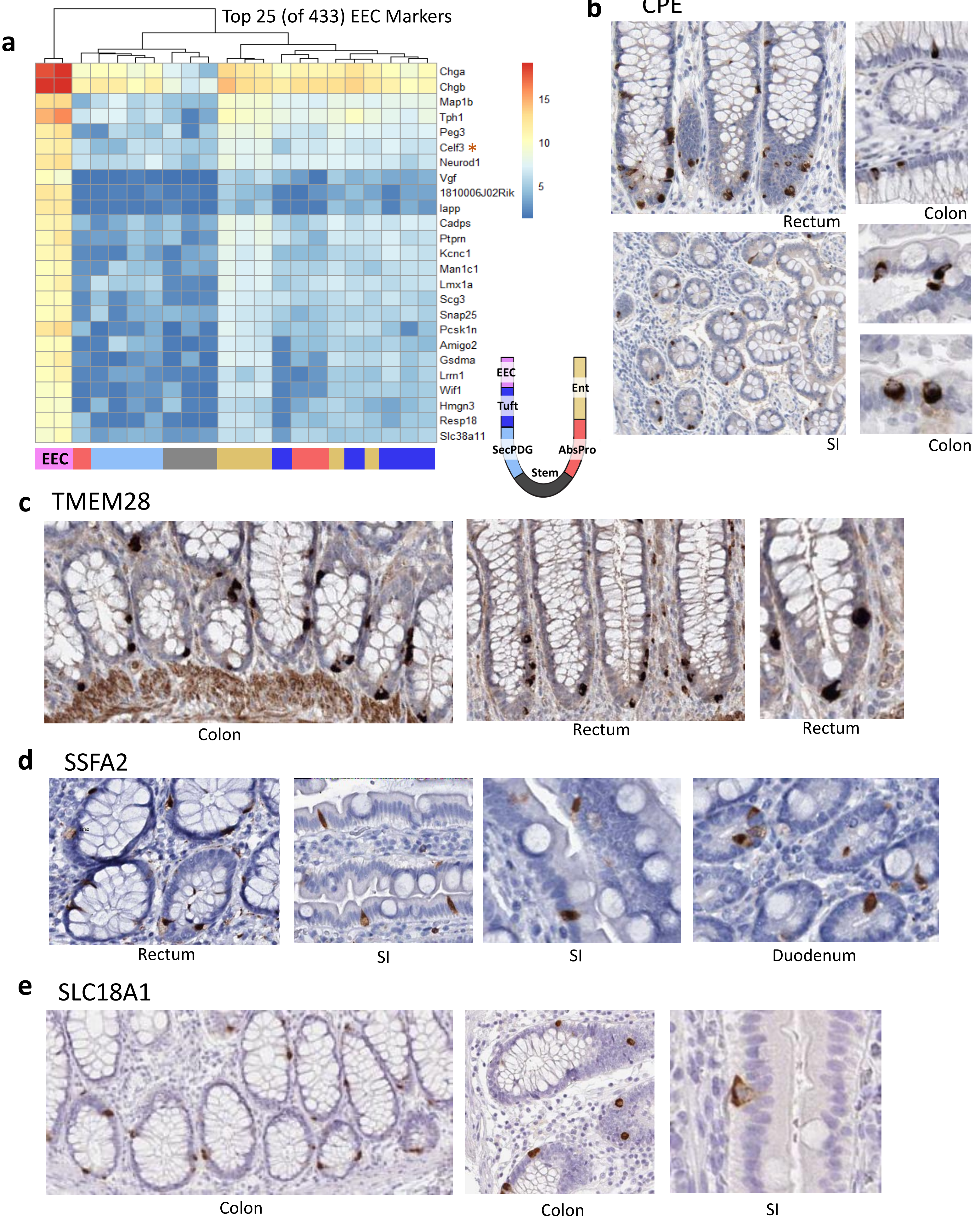

SUPPLEMENTARY FIGURE 8 continued

f CELF3

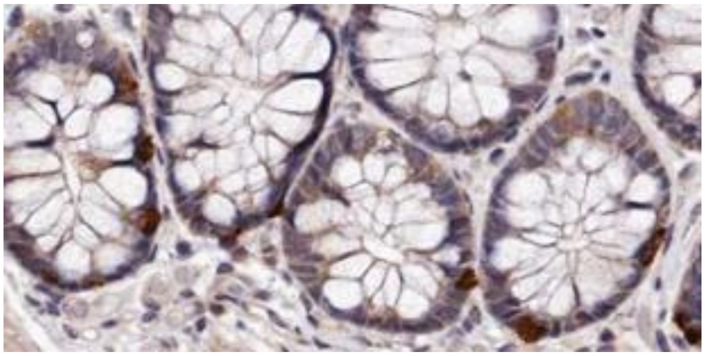

Colon

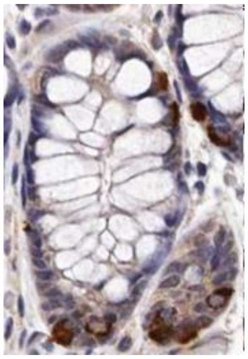

Colon

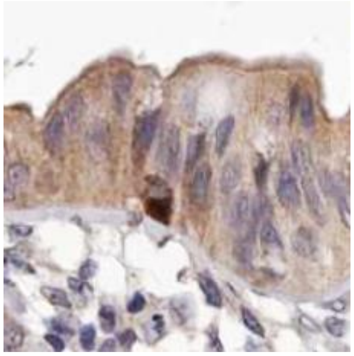

Colon

g

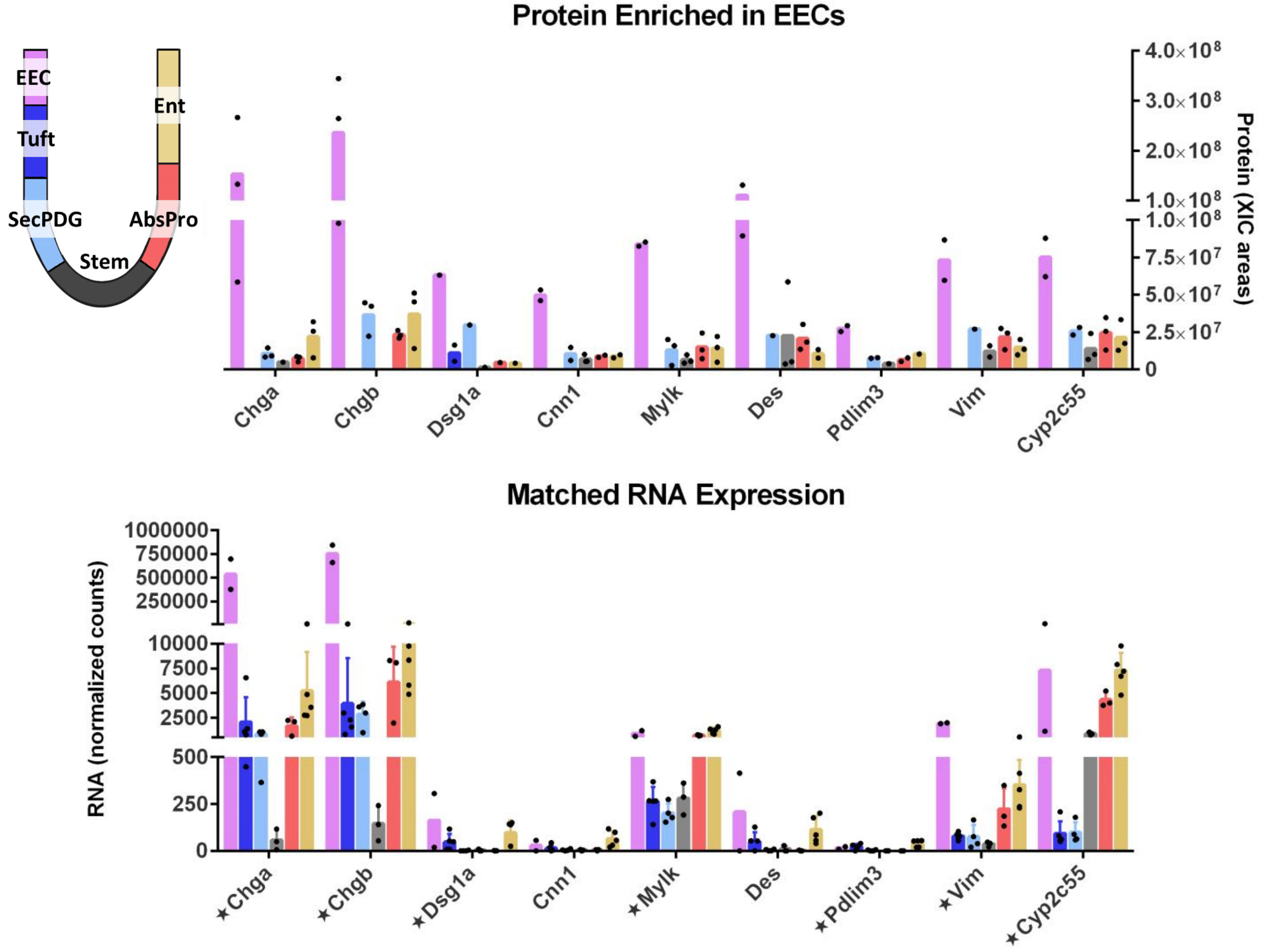

Supplementary Figure 8: Markers enriched in enteroendocrine cells. Our analysis identified n=433 genes as statistically enriched in EECs compared to the five other isolated colon crypt cell types (padj < 0.01 + minimum mean 50 counts). **a** Unsupervised clustering of the top 25 genes differentially expressed between EEC and non-EEC. Protein staining in human intestinal tissues is shown for genes with an orange asterisk. Human protein atlas staining showing unique EEC expression of the following markers **b** CPE, **c** TMEM28, **d** SSFA2, **e** SLC18A1, and **f** CELF3. **g** Using the MS proteomics data we identified proteins enriched in EECs which included known marker genes such as Chga and Chgb. Data is displayed in two graphs: protein expression and mRNA expression of the same genes. Star annotation by gene name symbolizes significant differential mRNA expression in at least one cell type compared to stem (padj < 0.01) and error bars are standard deviation. For proteomics there are n=3 independent biological replicates for each cell type; for mRNA differential expression analysis the following biological replicate numbers were used: stem=3, AbsPro=3, SecPDG=4, tuft=5, Ent=5, and EEC=2.

SUPPLEMENTARY FIGURE 9

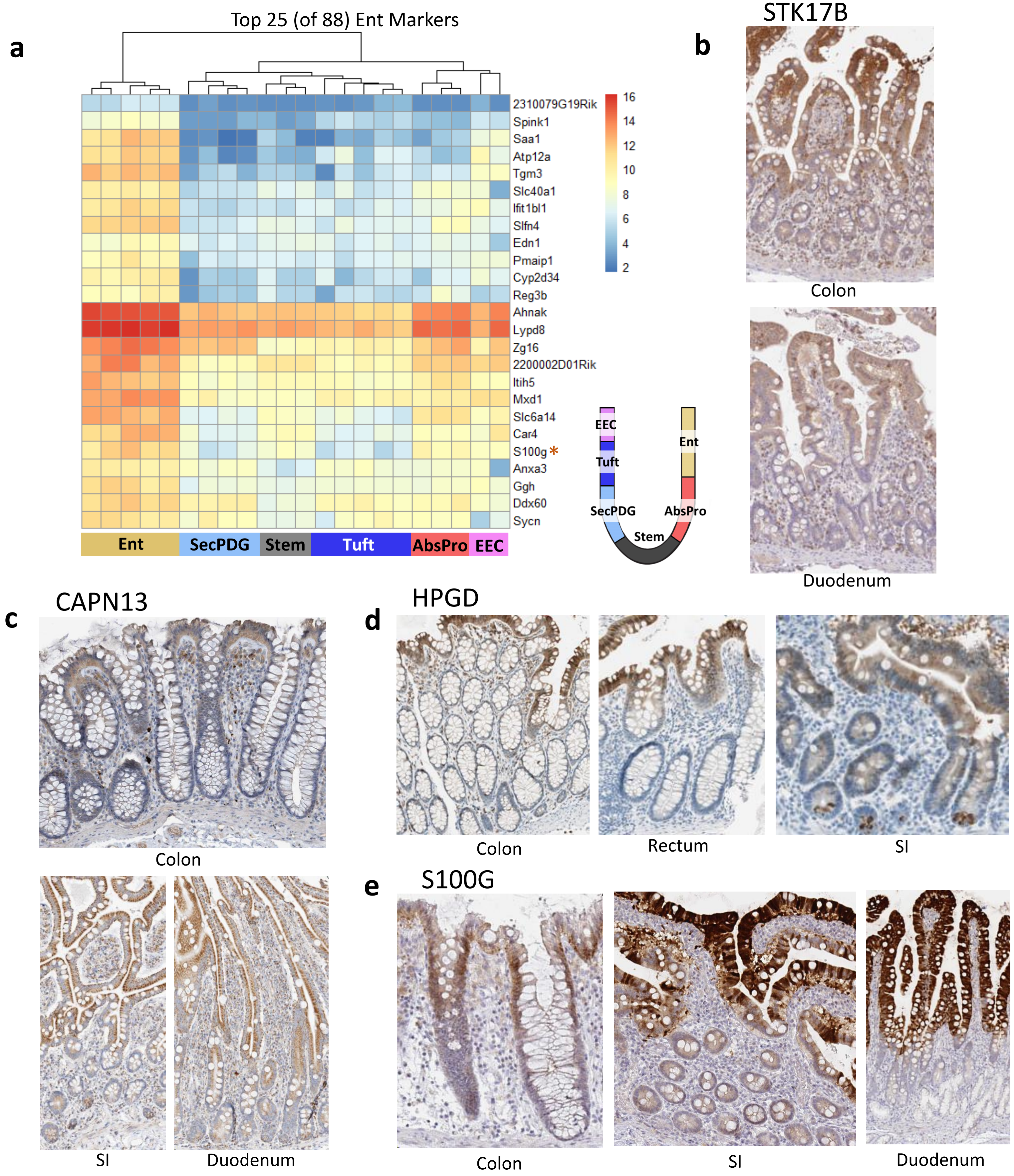

SUPPLEMENTARY FIGURE 9 continued

f RNASEL

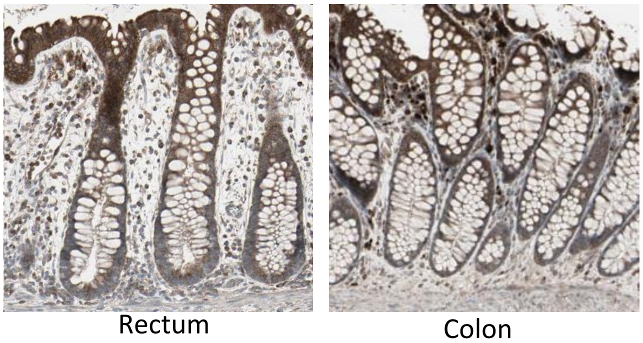

g

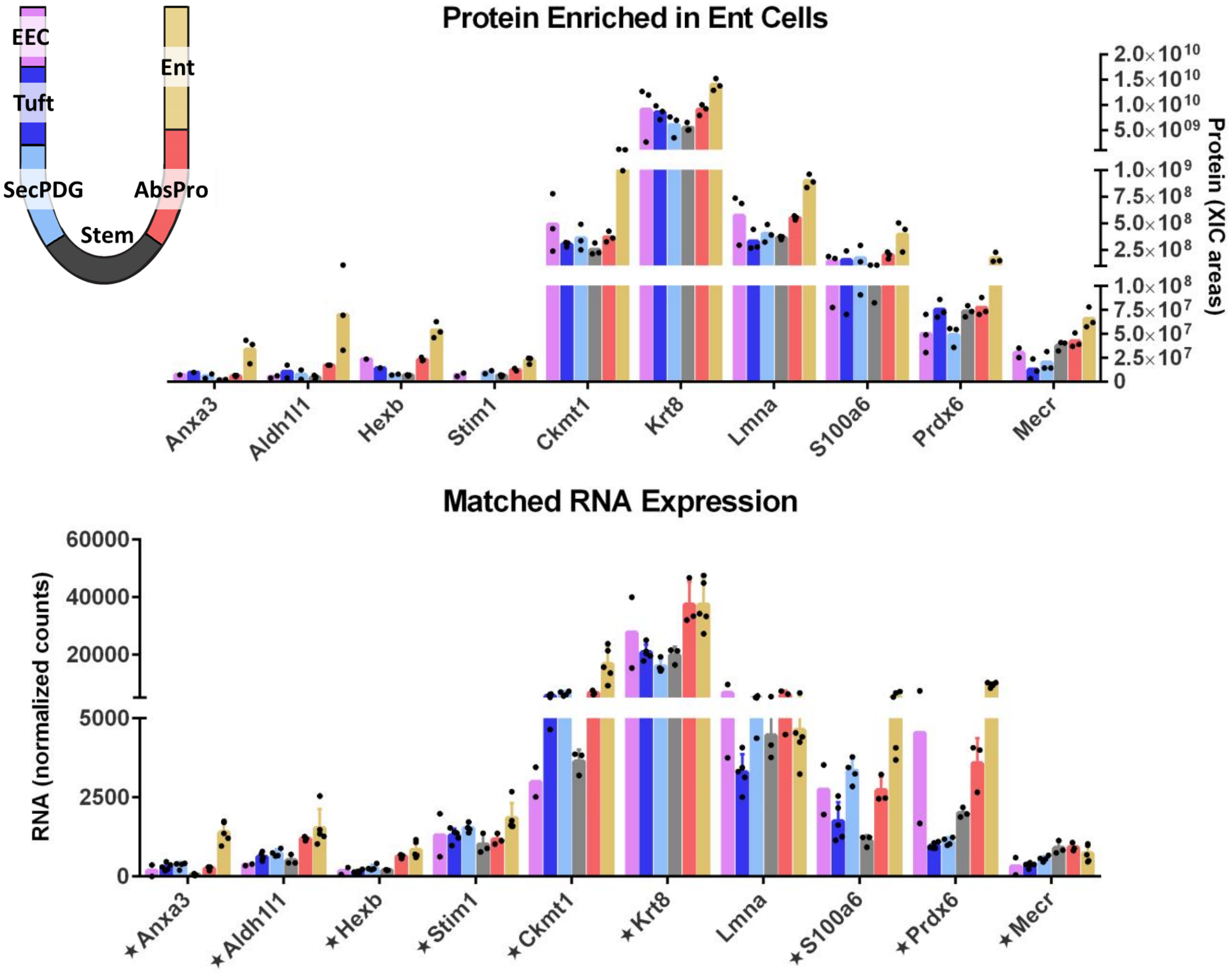

Supplementary Figure 9: Markers enriched in enterocytes. There were n=88 genes statistically enriched in the Ent population compared to the five other isolated colon crypt cell types (padj < 0.01 + minimum mean 50 counts). **a** Unsupervised clustering of the top 25 genes differentially expressed between Ent and non-Ent. Protein staining is shown for genes with orange asterisk. Human protein atlas images show enriched protein staining in Ent at the top of the crypt for the following markers **b** STK17B, **c** CAPN13, **d** HPGD, **e** S100G and **f** RNASEL. **g** Using the MS proteomics data we identified proteins enriched in the Ent population. Data is displayed in two graphs: protein expression and mRNA expression of the same genes. Star annotation by gene name symbolizes significant differential mRNA expression in at least one cell type compared to stem (padj < 0.01) and error bars are standard deviation. For proteomics there are n=3 independent biological replicates for each cell type; for mRNA differential expression analysis the following biological replicate numbers were used: stem=3, AbsPro=3, SecPDG=4, tuft=5, Ent=5, and EEC=2.

SUPPLEMENTARY FIGURE 10

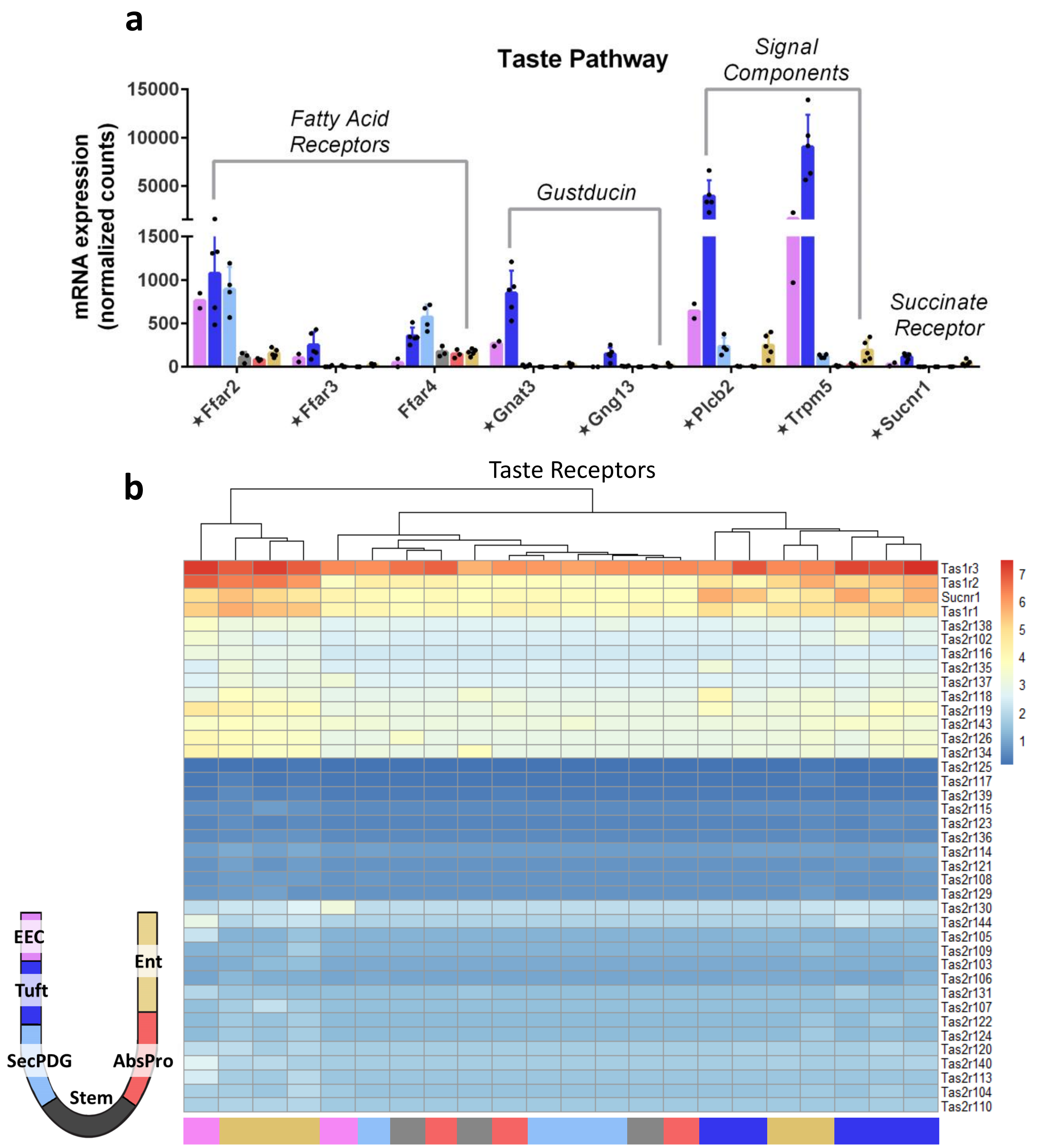

Supplementary Figure 10: Colonic tuft cells show a distinct potential for taste pathway activation via fatty acids. **a** mRNA expression of taste pathway signaling components elevated in tuft cells including three fatty acid receptors (*Ffar 2,3,4*), a primarily taste-specific G protein alpha-subunit (*Gnat3*; also known as Gustducin) and a related G protein subunit (*Gng13*) and cytoplasmic signaling components involved in taste signaling. Star annotation by gene name symbolizes significant differential mRNA expression in at least one cell type compared to stem (padj < 0.01) and error bars are standard deviation. For differential mRNA expression analysis, the following biological replicate numbers were used: stem=3, AbsPro=3, SecPDG=4, tuft=5, Ent=5, and EEC=2. **b** Taste receptor family expression with minimal expression of Tas2 receptors and non-tuft specific expression of Tas1 receptors. See *Supplementary Discussion* section for additional information.

SUPPLEMENTARY FIGURE 11

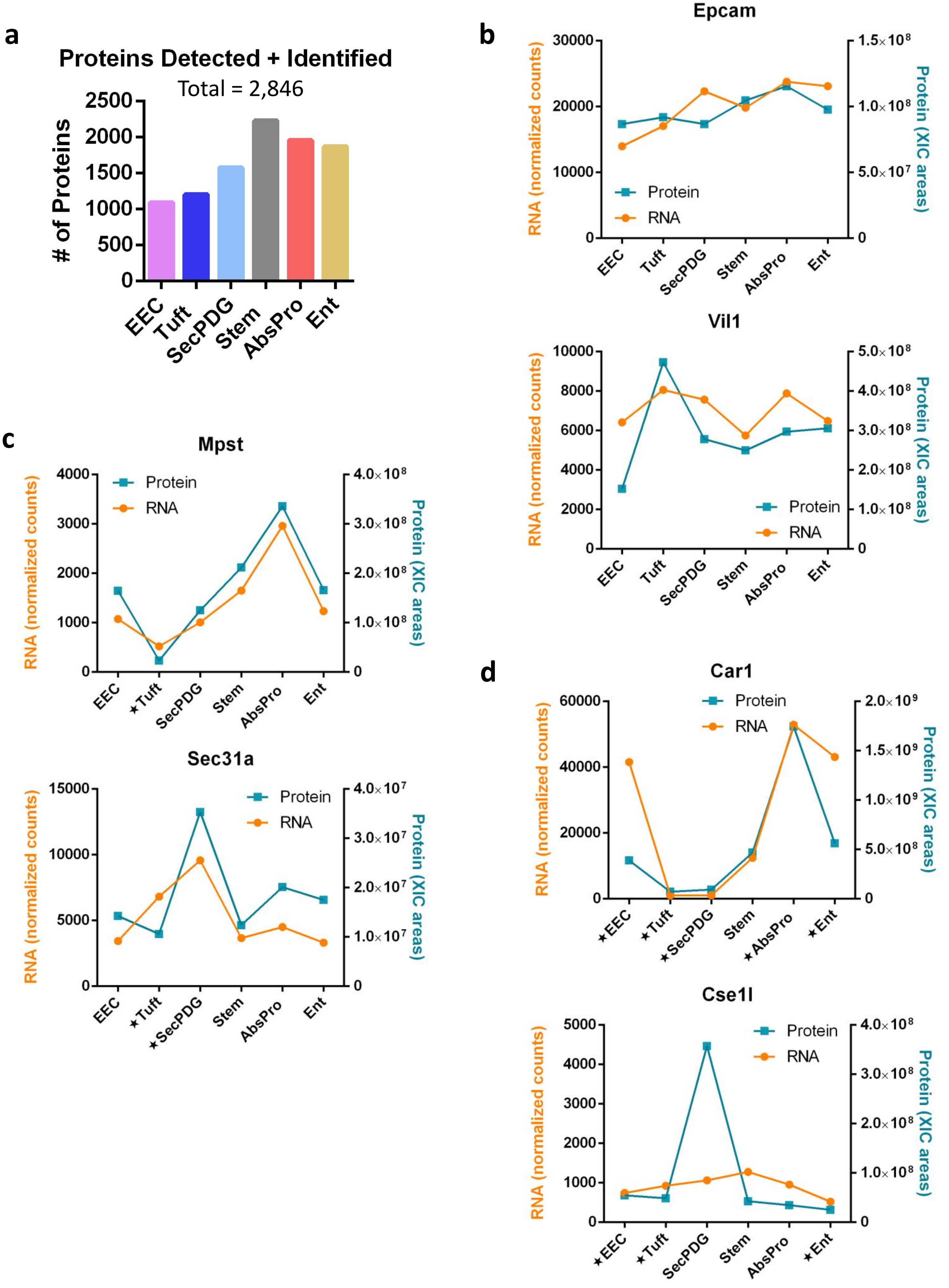

Supplementary Figure 11: Mass Spec analysis detected proteins expressed in the crypt cell types. **a** In total 2,846 proteins were detected and identified with more than 1,000 proteins in all cell types (including rare cell types such as tuft and EEC). **b** Protein from known genes expressed in the crypt, such as Epcam, were identified in all cell types and consistent with mRNA expression. Other genes such as Vil1, were also detected in all cell types at the protein level, but not as consistent with mRNA expression. **c** We identified genes that are markers of AbsPro (Mpst) and SecPDG (Sec31a) populations on both an mRNA and protein level. **d** We also observed some genes that were not consistent between mRNA and protein. for example, in AbsPro, Car1 protein is enriched whereas the mRNA is also enriched in Ent. Cse1l is only enriched in SecPDG on a protein level. Star annotation by gene name symbolizes significant differential mRNA expression in at least one cell type compared to stem (padj < 0.01). For differential mRNA expression analysis, the following independent biological replicate numbers were used: stem=3, AbsPro=3, SecPDG=4, tuft=5, Ent=5, and EEC=2.

SUPPLEMENTARY FIGURE 12

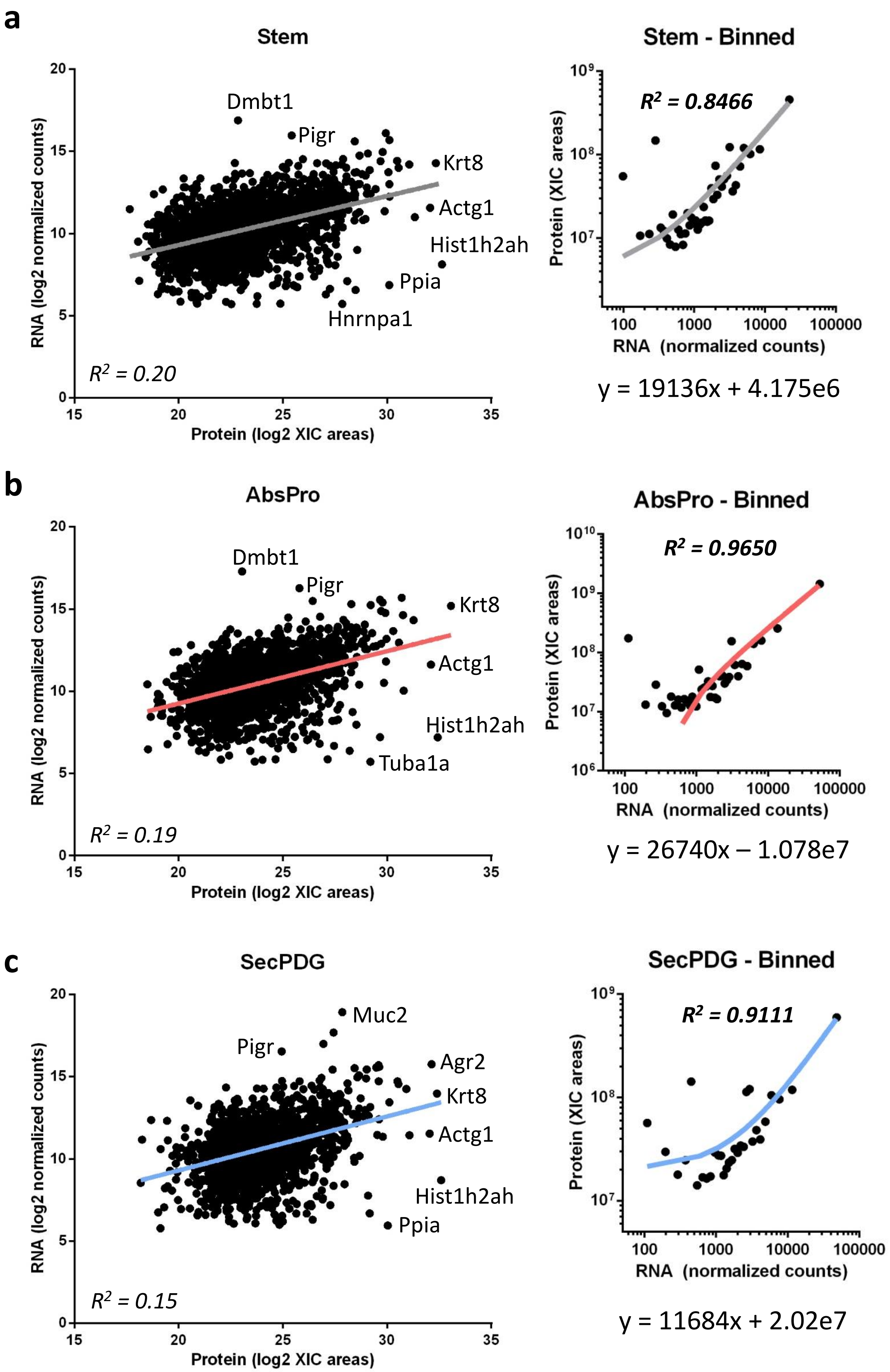

Top 10 Deviated Genes

a:
Hist1h2ah
Krt8
Actg1
Hist1h3b
Dmbt1
Atp5b
Hspa8
Pigr
Ppia
Atp5a1

b:
Krt8
Hist1h2ah
Actg1
Dmbt1
Atp5b
Pigr
Hist1h3b
Agr2
Atp5a1
Muc2

c:
Hist1h2ah
Muc2
Krt8
Actg1
Agr2
Clca1
Hist1h3b
Atp5b
Reg4
Atp5a1

SUPPLEMENTARY FIGURE 12 continued

d

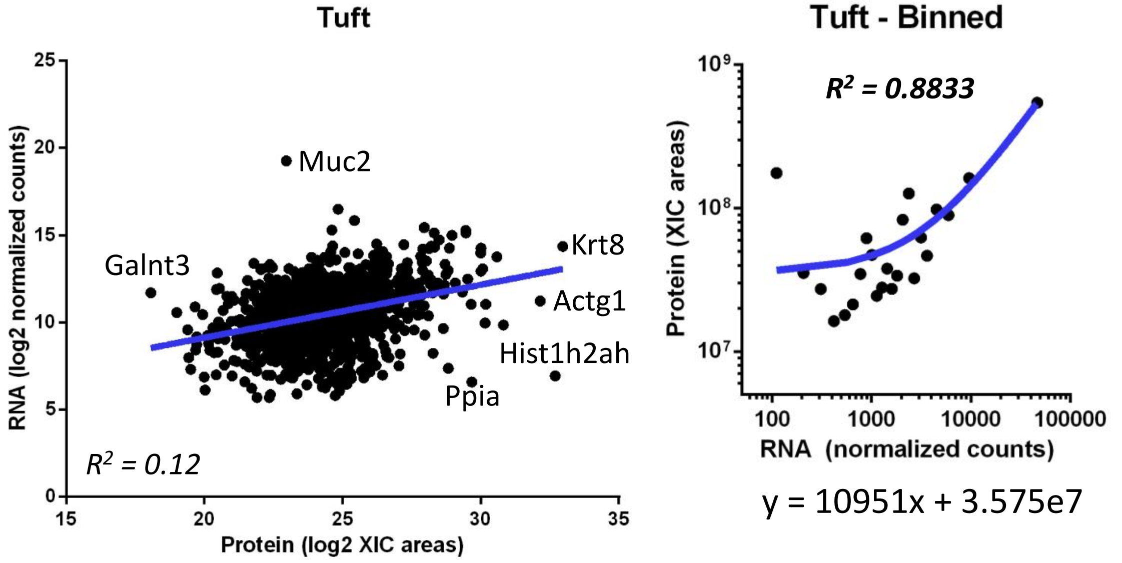

Top 10 Deviated Genes

Krt8
Hist1h2ah
Muc2
Actg1
Hist1h3b
Atp5b
Tuba1a
Hist2h2ac
Clca1
Hspa8

e

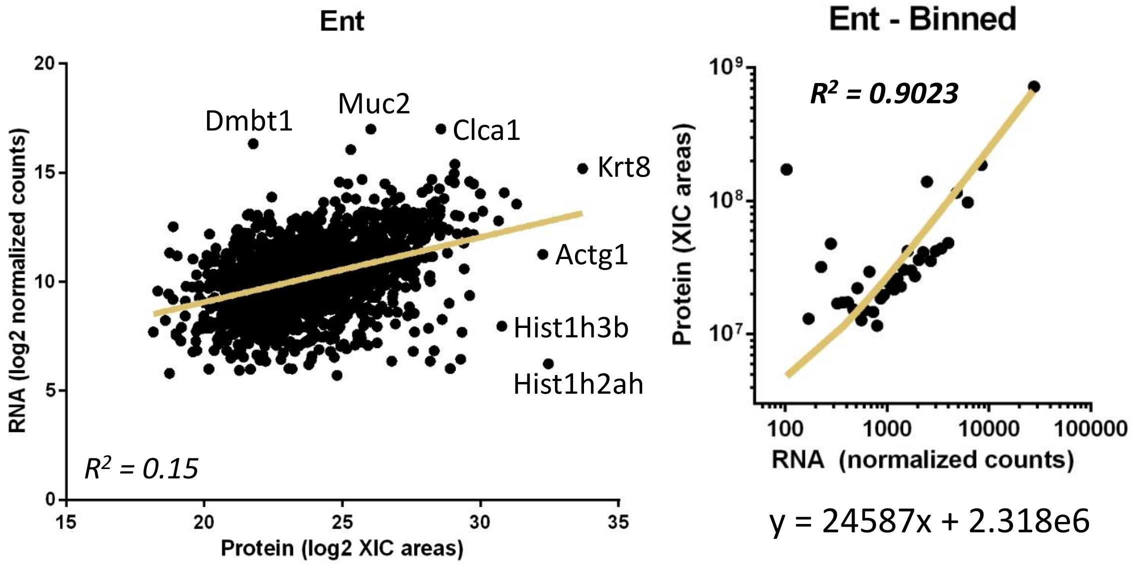

Krt8
Hist1h2ah
Actg1
Muc2
Clca1
Atp5b
Dmbt1
Hist1h3b
Pigr
Atp5a1

f

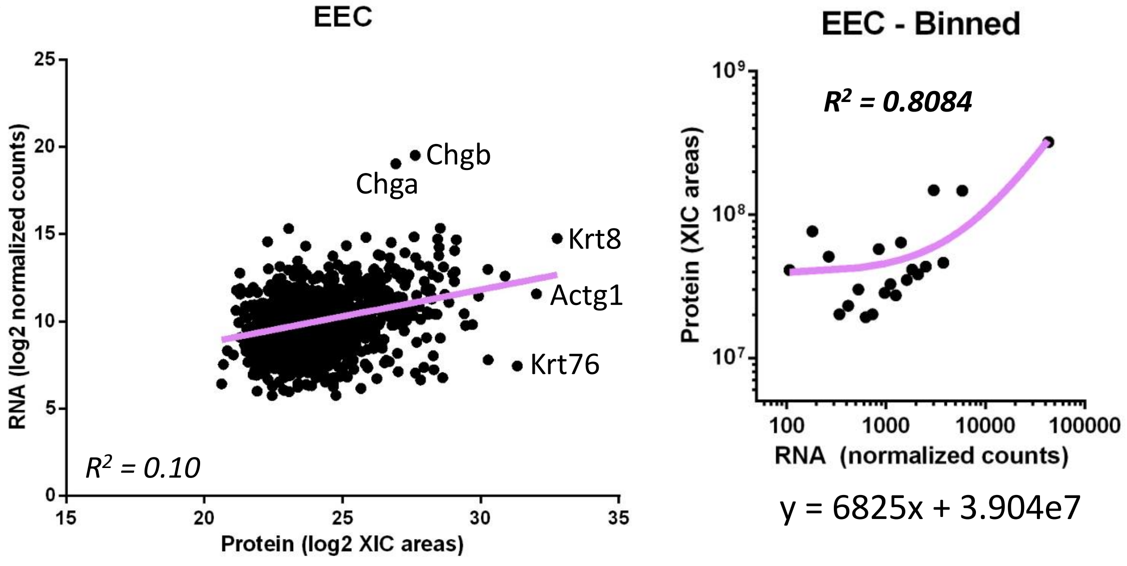

Krt8
Chgb
Actg1
Chga
Krt76
Atp5b
Hist1h3b
Atp5a1
Agr2
Hist2h2ac

Supplementary Figure 12: Comparison of mRNA to protein in each crypt cell type. Each gene that is detected on the protein level with MS and has mRNA expression >50 normalized counts is graphed for each cell type (scales are log2). A linear best fine line is drawn, however the R^2 values are poor. When the data is sorted based on mRNA expression and binned, with 50 genes in each bin, variation can be minimized and linear best fit lines fit the data well resulting in stronger R^2 values. Using the binned best fit line equation, we identified the top 10 deviating genes based on standard deviation of actual protein versus expected protein. The cell types include **a** Stem, **b** AbsPro, **c** SecPDG, **d** Tuft, **e** Ent, and **f** EEC. The strongest binned R^2 value was observed in AbsPro (R^2 = 0.965).

SUPPLEMENTARY FIGURE 13

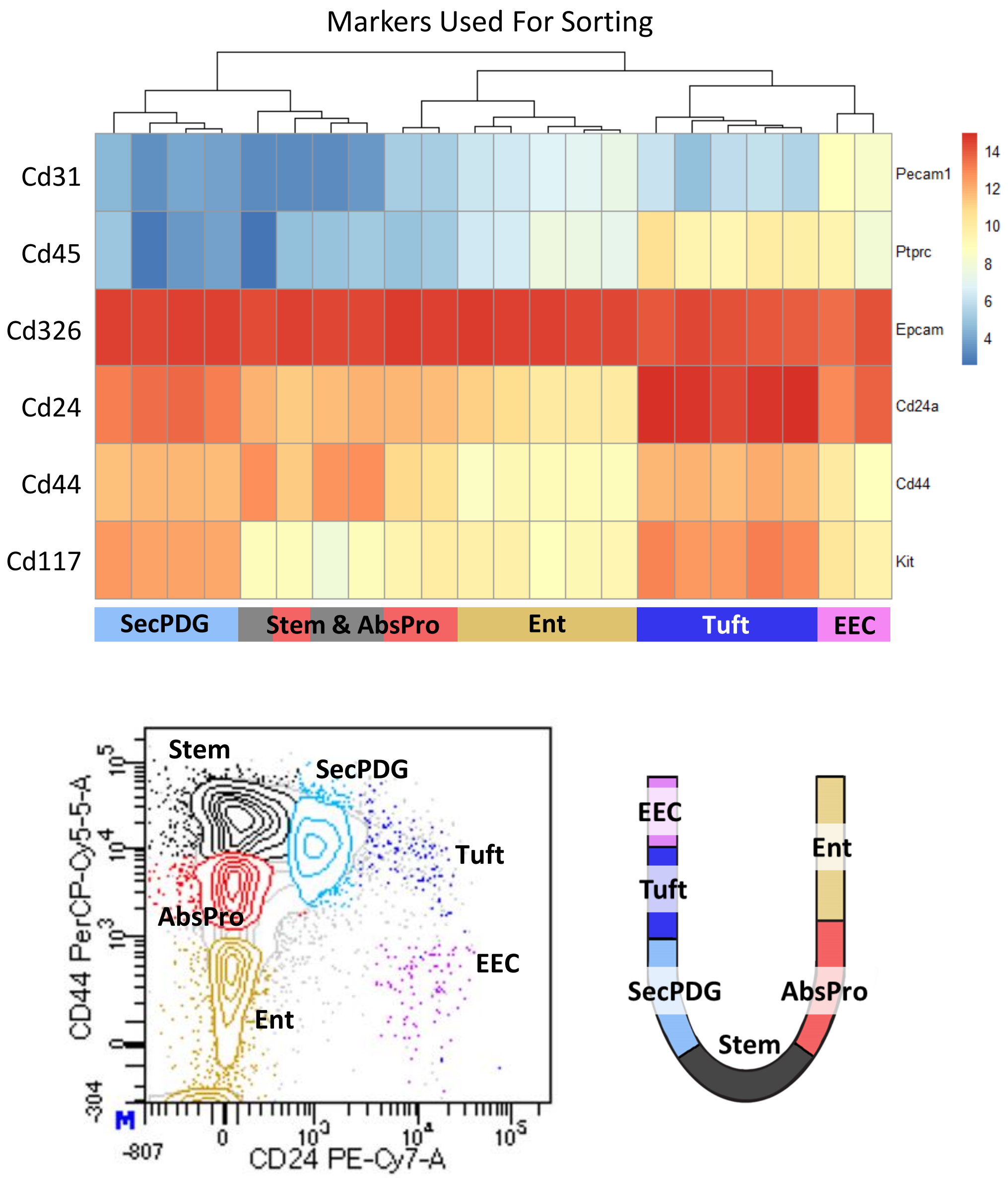

Supplementary Figure 13: Surface marker protein expression correlations with mRNA gene expression. Unsupervised clustering of mRNA gene expression of surface markers used for sorting. The dump channel includes Cd31 and Cd45 (to discard endothelial and immune system cells, respectively), whereas positive markers Cd326, Cd24, Cd44, and Cd117 are used to distinguish and purify their respective populations. Although no Cd45 protein was detected in tuft cells (Supplementary Figure 7f), a low, but clearly detectable level of mRNA (*Ptprc)* was detected and elevated in tuft cells compared to other isolated colon crypt cell types.

SUPPLEMENTARY FIGURE 14

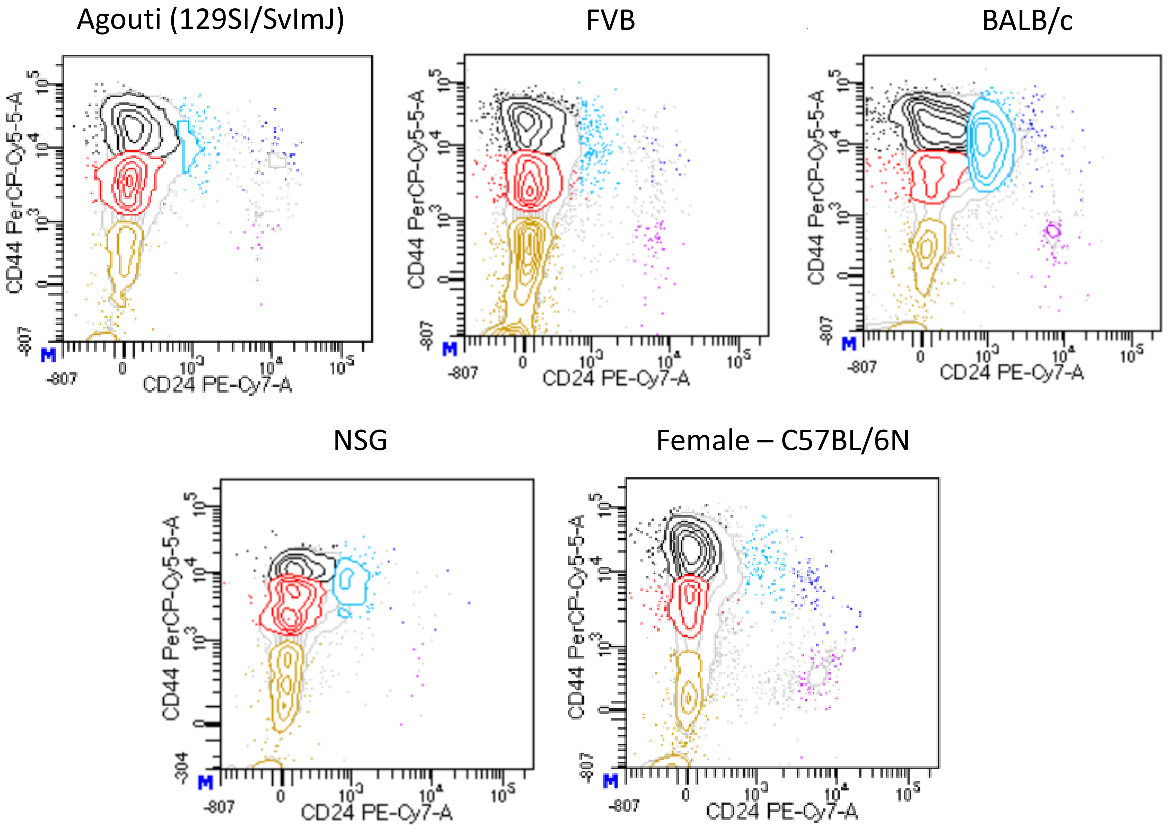

Supplementary Figure 14: Sorting procedure is universal and resolves cell types in the colon of other mouse strains and gender including Agoutti, FVB, Balbc, NSG, and females. Each FACS plot is a representative image from one mouse, n=3 independent sorts.

SUPPLEMENTARY FIGURE 15

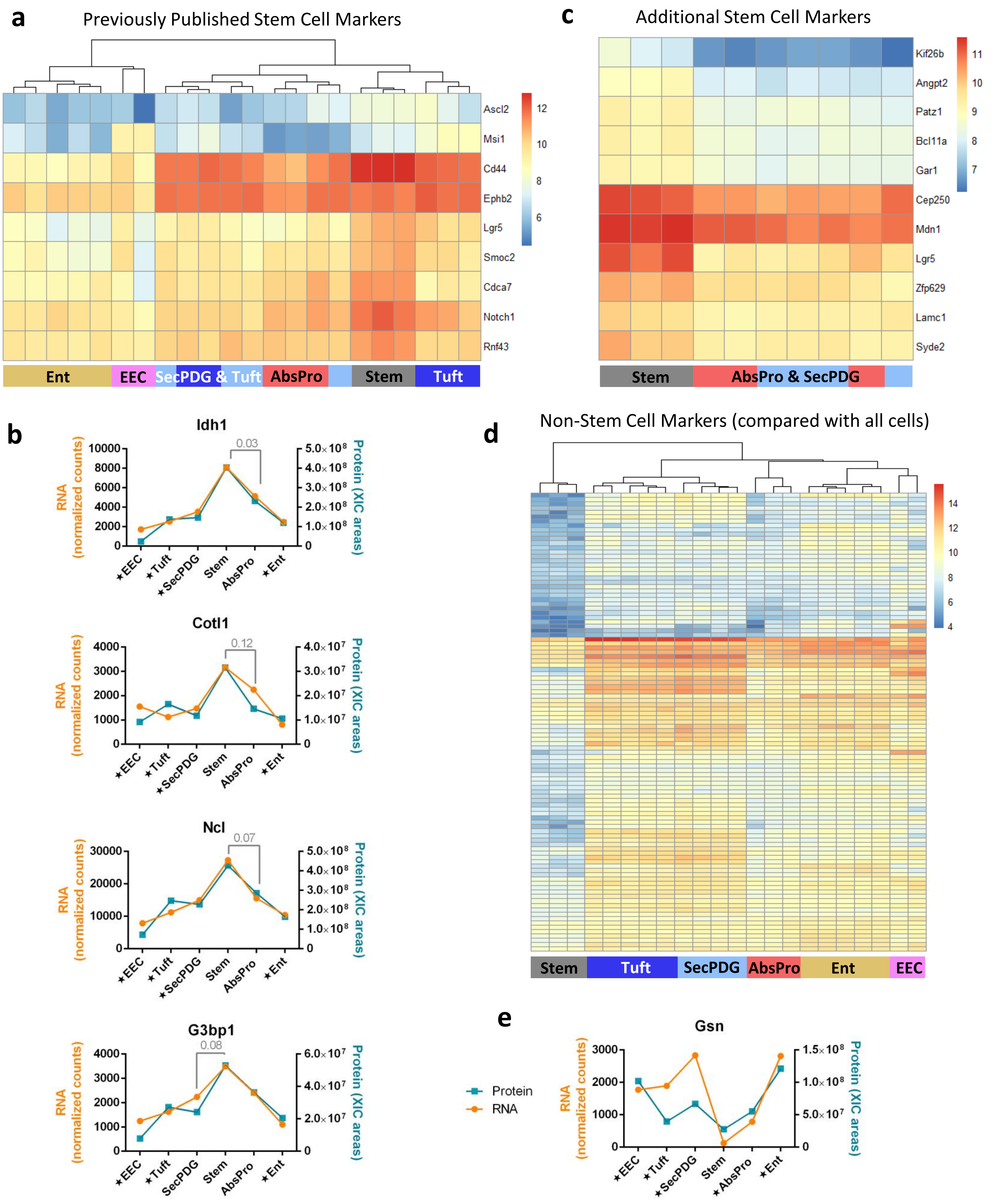

Supplementary Figure 15: Additional markers of intestinal stemness. **a** Unsupervised clustering of known/classically defined intestinal stem cell markers. **b** mRNA and protein expression of markers elevated in stem, but not passing the stringent mRNA padj < 0.01 cut-off between stem and progenitors. These marker genes continue to decrease in mRNA and protein expression and are lowest in the most differentiated cell types. **c** When the analysis of stem versus non-stem in Figure 2c is performed *only* between stem versus AbsPro and SecPDG, n=11 additional stem markers are identified for a total of n=27 gene expression differences that distinguish stem cells from progenitors. **d** Unsupervised clustering of n=107 genes that were significantly elevated in expression in all non-stem cells (SecPDG, AbsPro, Ent, tuft, EEC) compared to stem cells (see Supplementary Data #3 for gene list). The highly abundant *Chga* and *Chgb* mRNAs were not included in the heatmap. Their expression is several logs greater than all the genes shown here. **e** Gsn is one of the n=107 differentiation genes that is elevated compared to stem and graph displays mRNA and protein expression. Star annotation by cell type symbolizes significant differential mRNA expression compared to stem (padj < 0.01). For differential mRNA expression analysis, the following independent biological replicate numbers were used: stem=3, AbsPro=3, SecPDG=4, tuft=5, Ent=5, and EEC=2.

SUPPLEMENTARY FIGURE 16

a

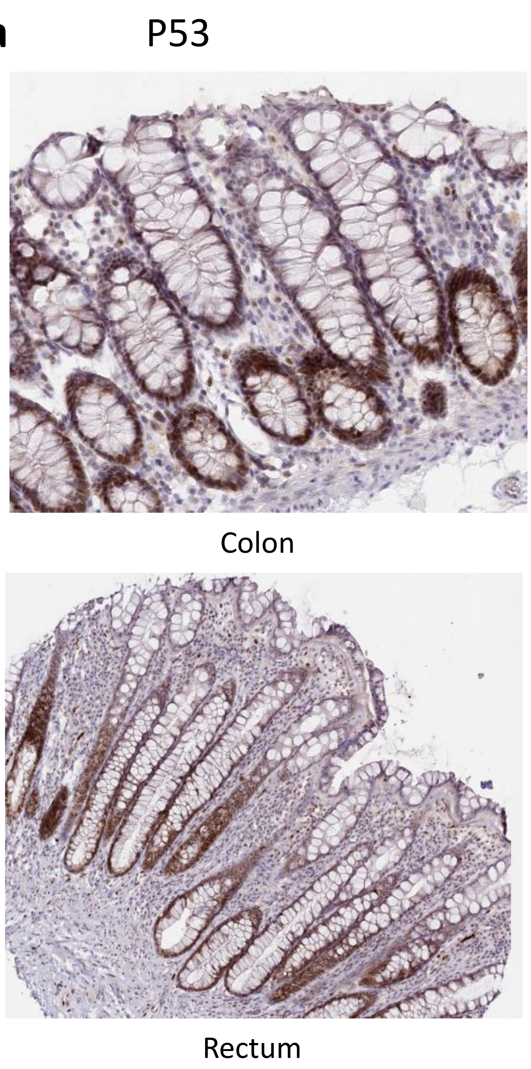

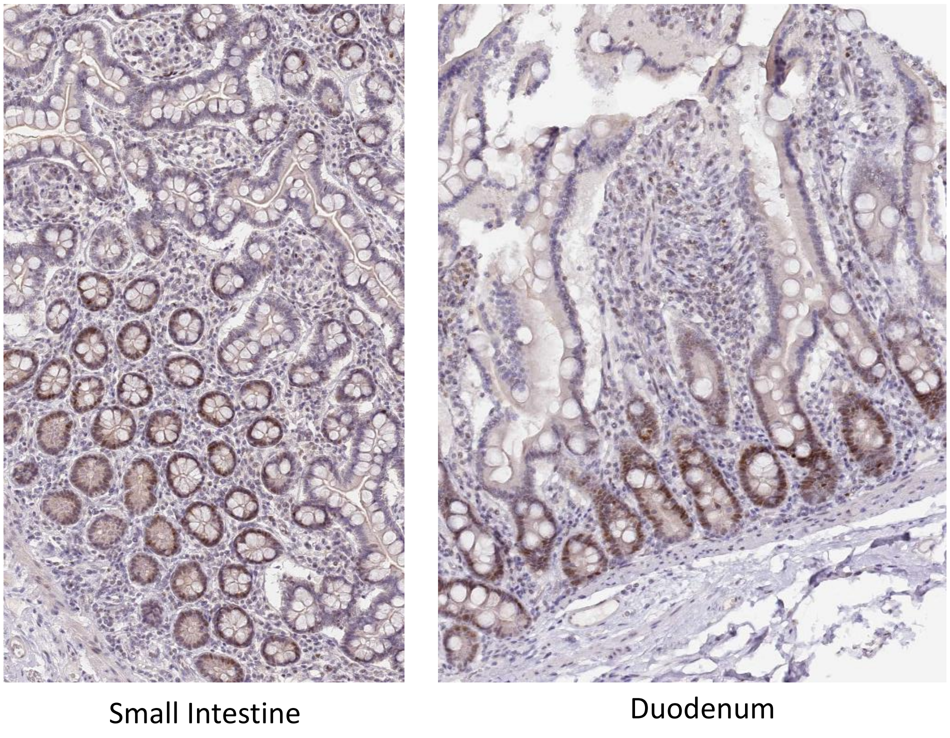

b

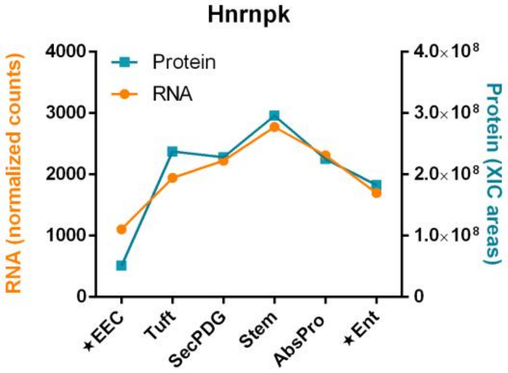

c

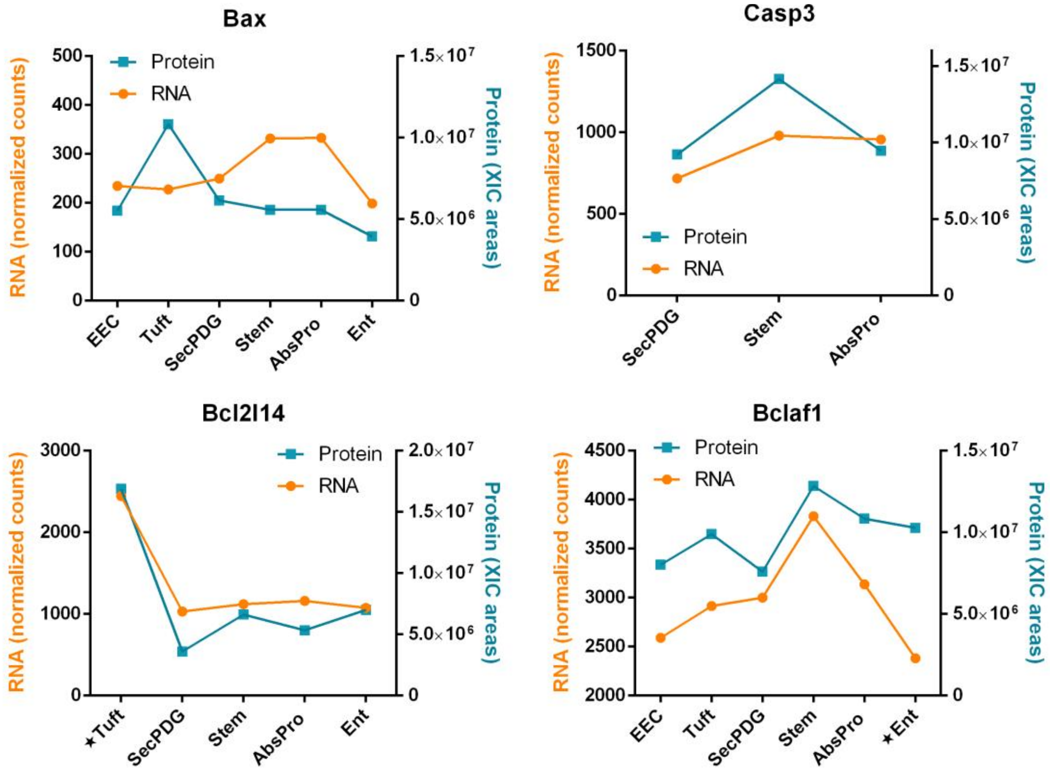

Supplementary Figure 16: Apoptosis related gene expression in the intestinal crypt. **a** Human protein atlas images showing TP53 protein levels are highest at the base of crypts in the colon, rectum, small intestine, and duodenum. **b** Protein and mRNA expression of Hnrnpk, a pre-mRNA binding protein. Hnrnpk acts as a transcriptional co-activator of p53 when SUMOylated [7]. Protein and mRNA expression for additional apoptosis related genes are shown in **c**. Bax promotes activation of Casp3 (protein only detected in stem and progenitors) which triggers apoptosis. Bcl2l14 also contributes to apoptosis and Bclaf1 is a death-promoting transcriptional repressor. Star annotation by cell type symbolizes significant differential mRNA expression compared to stem (padj < 0.01). For differential mRNA expression analysis, the following independent biological replicate numbers were used: stem=3, AbsPro=3, SecPDG=4, tuft=5, Ent=5, and EEC=2.

SUPPLEMENTARY FIGURE 17

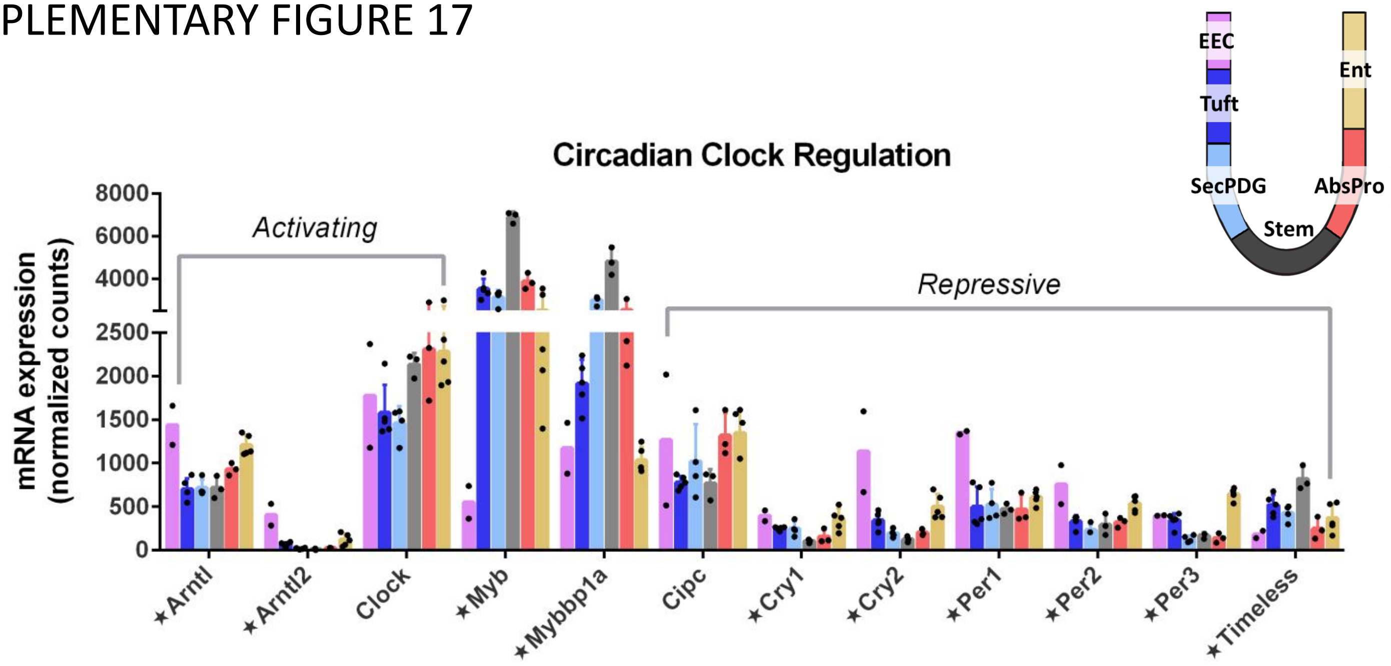

Supplementary Figure 17: Regulation of the circadian clock in the crypt. *Arntl* (Bmal1) and *Clock* are highly expressed in all cell types, including stem cells. In general, clock repressors are expressed at a lower level. Myb and Mybbp1a functions in the circadian clock are less well characterized, but expression is enriched in stem. Star annotation by gene name symbolizes significant differential mRNA expression in at least one cell type compared to stem (padj < 0.01) and error bars are standard deviation. For differential mRNA expression analysis, the following biological replicate numbers were used: stem=3, AbsPro=3, SecPDG=4, tuft=5, Ent=5, and EEC=2.

SUPPLEMENTARY FIGURE 18

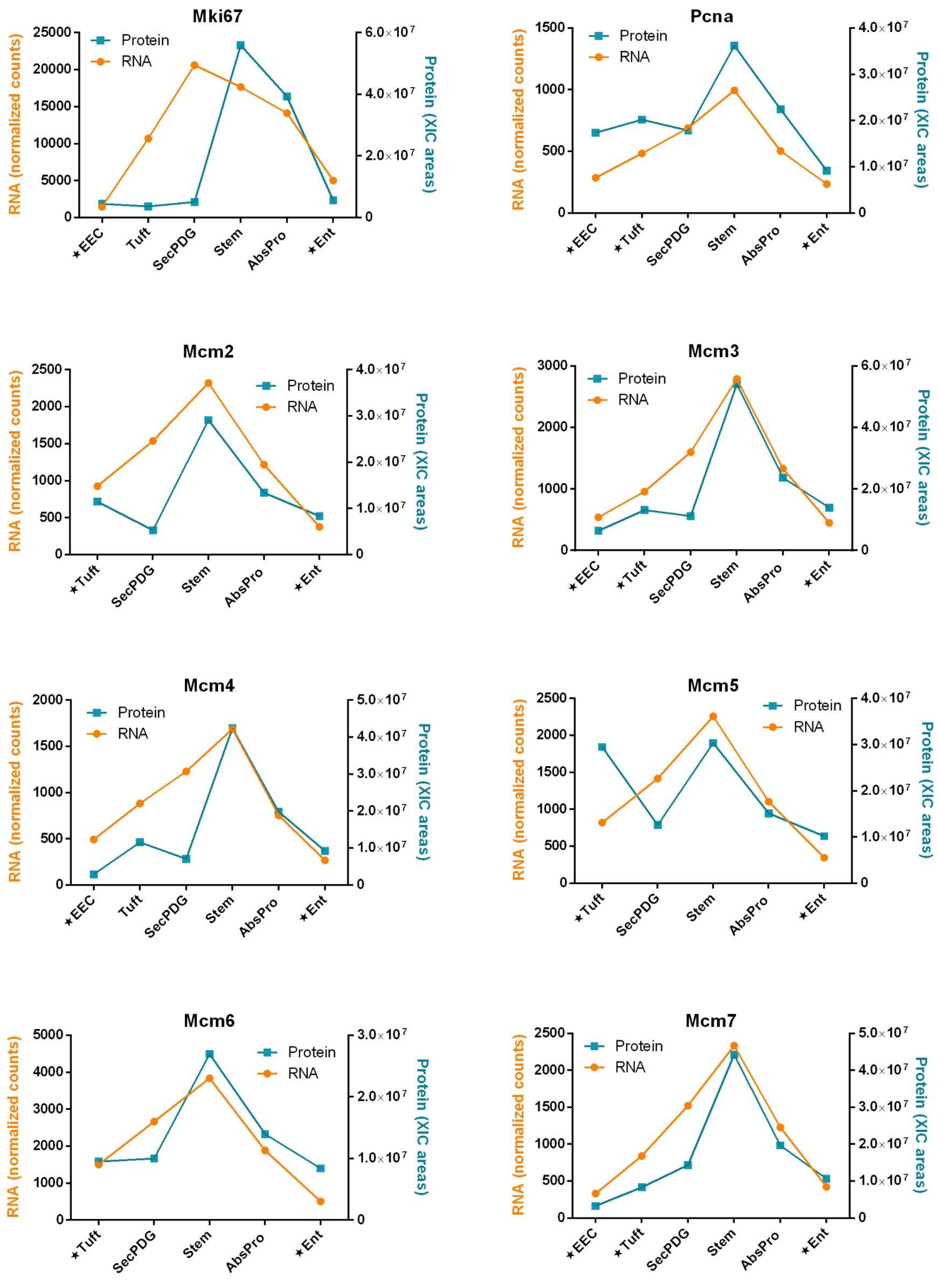

Supplementary Figure 18: Expression of proliferation and cell cycle genes. Expression of Mki67 (mitotic chromosome stability), Pcna (DNA replication control), and Mcm2-7 (DNA replication licensing factors) are highest in both stem mRNA and protein. Star annotation by cell type symbolizes significant differential mRNA expression compared to stem (padj < 0.01). For differential mRNA expression analysis, the following biological replicate numbers were used: stem=3, AbsPro=3, SecPDG=4, tuft=5, Ent=5, and EEC=2.

SUPPLEMENTARY FIGURE 19

a

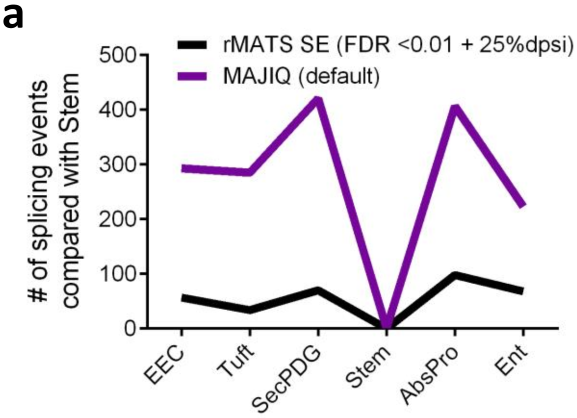

b

		Percent of each Splicing Event				
Cell Type (Compared with Stem)	Total Number of Splicing Events	SE	RI	MXE	A5SS	A3SS
AbsPro	1157	80.03	2.42	4.93	4.49	8.12
SecPDG	832	73.44	2.40	8.77	6.01	9.38
Tuft	542	73.99	1.85	11.25	6.09	6.83
Ent	791	74.08	3.03	9.23	5.56	8.09
EEC	337	64.09	6.53	9.50	6.82	13.06
	Average Percent	73.13	3.25	8.74	5.80	9.09

c

Comparison	Gene Expression (Padj <0.01)	mRNA Splicing - SE Events (FDR <0.05)	SAR = Splicing : Gene ratio x 100
Stem to AbsPro	301	926	308
AbsPro to Ent	5,201	489	9
Stem to Ent	7,138	586	8
Stem to SecPDG	1,669	611	37
SecPDG to Tuft	1,546	138	9
SecPDG to EEC	8,887	137	2
Stem to Tuft	3,615	401	11
Stem to EEC	8,417	216	3

d

Comparison	Gene Expression (Padj <0.01)	mRNA Polyadenylation Events (Padj <0.05)	PAR = Poly(A) : Gene ratio x 100
Stem to AbsPro	301	795	264
AbsPro to Ent	5,201	185	4
Stem to Ent	7,138	217	3
Stem to SecPDG	1,669	657	39
SecPDG to Tuft	1,546	302	20
SecPDG to EEC	8,887	415	5
Stem to Tuft	3,615	401	11
Stem to EEC	8,417	467	6

Supplementary Figure 19: Alternative mRNA processing changes in the intestinal crypt. **a** Alternative splicing events in non-stem cells compared to stem cells as determined by an additional splicing pipeline, MAJIQ, or stringent filtering with rMATS. **b** Table showing the total number of splicing events detected by rMATS in each cell type compared to stem cells. There are a total of five different types of splicing events (SE = Skipped Exon, RI = Retained Intron, MXE = Mutually Exclusive Exon, A5SS = Alt 5 Splice Site, A3SS = Alt 3 Splice Site). The table provides the averages of each type of splicing event shown in the Figure 3b pie chart. SE events are the most abundant type of splicing event. **c** Splicing Abundance Ration (SAR) and **d** Polyadenylation Abundance Ratio (PAR) are metrics that report the relative abundance of specific RNA processing changes relative to changes in mRNA levels (i.e. gene expression). Statistical cut-offs for gene expression (padj < 0.01 + minimum mean 50 counts) and splicing (FDR < 0.05) or polyadenylation (padj < 0.01) values were used to generate the SAR and PAR values in Figure 3c and 3f, respectively. For differential mRNA expression and processing analyses, the following biological replicate numbers were used: stem=3, AbsPro=3, SecPDG=4, tuft=5, Ent=5, and EEC=2.

SUPPLEMENTARY FIGURE 20

a Splicing

Reactome pathways	Fold Enrichment	FDR	Number of Genes
Phase 3 - rapid repolarisation (R-MMU-5576890)	25.65	2.79E-02	3
Synthesis of pyrophosphates in the cytosol (R-MMU-1855167)	20.52	3.52E-02	3
SUMOylation of RNA binding proteins (R-MMU-4570464)	10.36	1.65E-02	5
SUMOylation of SUMOylation proteins (R-MMU-4085377)	10.36	1.58E-02	5
SUMOylation of DNA replication proteins (R-MMU-4615885)	9.33	9.91E-03	6
SUMOylation of ubiquitinylation proteins (R-MMU-3232142)	9.00	2.39E-02	5
Transcriptional activity of SMAD2/SMAD3:SMAD4 heterotrimer (R-MMU-2173793)	8.34	3.05E-02	5
Inositol phosphate metabolism (R-MMU-1483249)	7.43	3.59E-02	5
Nuclear Envelope Breakdown (R-MMU-2980766)	7.12	4.17E-02	5
SUMOylation of DNA damage response and repair proteins (R-MMU-3108214)	6.47	1.44E-02	7
CLEC7A (Dectin-1) signaling (R-MMU-5607764)	6.22	1.01E-02	8
Biosynthesis of the N-glycan precursor (dolichol lipid-linked oligosaccharide, LLO) and transfer to a nascent protein (R-MMU-446193)	5.95	3.55E-02	6
SUMOylation (R-MMU-2990846)	5.89	2.56E-04	13
SUMO E3 ligases SUMOylate target proteins (R-MMU-3108232)	5.66	7.13E-04	12
Transport of Mature Transcript to Cytoplasm (R-MMU-72202)	5.47	4.42E-02	6
Transport of Mature mRNA derived from an Intron-Containing Transcript (R-MMU-159236)	5.47	4.31E-02	6
Mitotic Prophase (R-MMU-68875)	5.32	2.96E-02	7
C-type lectin receptors (CLRs) (R-MMU-5621481)	5.07	2.07E-02	8
Chromatin modifying enzymes (R-MMU-3247509)	4.11	1.34E-02	11
Chromatin organization (R-MMU-4839726)	4.11	1.26E-02	11
Separation of Sister Chromatids (R-MMU-2467813)	3.70	3.11E-02	10
Mitotic Anaphase (R-MMU-68882)	3.64	3.38E-02	10
Mitotic Metaphase and Anaphase (R-MMU-2555396)	3.62	3.30E-02	10
Asparagine N-linked glycosylation (R-MMU-446203)	3.34	1.24E-02	14
Processing of Capped Intron-Containing Pre-mRNA (R-MMU-72203)	3.26	3.68E-02	11
M Phase (R-MMU-68886)	3.21	1.00E-02	16
Cell Cycle Checkpoints (R-MMU-69620)	3.13	3.27E-02	12
Antigen processing: Ubiquitination & Proteasome degradation (R-MMU-983168)	3.04	2.20E-02	14
Class I MHC mediated antigen processing & presentation (R-MMU-983169)	2.96	1.51E-02	16
Cell Cycle (R-MMU-1640170)	2.78	4.14E-03	22
Cell Cycle, Mitotic (R-MMU-69278)	2.75	1.20E-02	19
Generic Transcription Pathway (R-MMU-212436)	2.52	3.06E-03	27
Gene expression (Transcription) (R-MMU-74160)	2.49	4.16E-04	35
RNA Polymerase II Transcription (R-MMU-73857)	2.41	3.05E-03	30
Post-translational protein modification (R-MMU-597592)	2.37	5.76E-05	46
Metabolism of proteins (R-MMU-392499)	2.19	5.02E-05	55
Metabolism (R-MMU-1430728)	2.07	1.67E-04	55

b APA

Reactome pathways	Fold Enrichment	FDR	Number of Genes
SUMO is conjugated to E1 (UBA2:SAE1) (R-MMU-3065676)	77.42	4.80E-02	2
Energy dependent regulation of mTOR by LKB1-AMPK (R-MMU-380972)	20.02	2.26E-03	5
mTOR signalling (R-MMU-165159)	18.34	7.80E-04	6
CREB phosphorylation through the activation of Ras (R-MMU-442742)	17.20	1.82E-02	4
Post NMDA receptor activation events (R-MMU-438064)	13.66	2.88E-02	4
Macroautophagy (R-MMU-1632852)	12.32	9.70E-04	7
Activation of NMDA receptor and postsynaptic events (R-MMU-442755)	12.22	3.61E-02	4
TP53 Regulates Metabolic Genes (R-MMU-5628897)	11.85	1.42E-02	5
Mitotic Spindle Checkpoint (R-MMU-69618)	7.26	1.20E-02	7
SUMOylation (R-MMU-2990846)	5.38	3.63E-02	7
Separation of Sister Chromatids (R-MMU-2467813)	5.02	3.03E-02	8
Mitotic Anaphase (R-MMU-68882)	4.94	3.14E-02	8
Mitotic Metaphase and Anaphase (R-MMU-2555396)	4.92	3.04E-02	8
Mitotic Prometaphase (R-MMU-68877)	4.84	2.99E-02	8
Cellular responses to external stimuli (R-MMU-8953897)	4.47	1.65E-03	16
Cell Cycle, Mitotic (R-MMU-69278)	4.18	8.96E-04	17
M Phase (R-MMU-68886)	4.09	9.84E-03	12
Cell Cycle (R-MMU-1640170)	3.86	8.51E-04	18
Metabolism of RNA (R-MMU-8953854)	3.02	3.59E-02	13
Metabolism of lipids (R-MMU-556833)	2.86	2.47E-02	16
RNA Polymerase II Transcription (R-MMU-73857)	2.46	3.44E-02	18
Gene expression (Transcription) (R-MMU-74160)	2.42	3.65E-02	20
Metabolism (R-MMU-1430728)	2.11	1.05E-02	33
Unclassified (UNCLASSIFIED)	0.72	1.81E-03	80

Supplementary Figure 20: Gene ontology analysis of alternatively processed mRNA in stem cells and progenitors. **a** Complete list of enriched gene ontologies for n=332 genes that are alternatively processed (spliced) in common between stem versus AbsPro and stem versus SecPDG. **b** Complete list of enriched gene ontologies for n=194 genes that are alternatively polyadenylated in common between stem versus AbsPro and stem versus SecPDG. Shortened list of ontology is presented in Figure 4c.

SUPPLEMENTARY FIGURE 21

a

	In AbsPro + SecPDG			
**Gene (n=13)**	**SE event**	**terminal exon?**	**APA loci**	**Note:**
Metap1	chr3:138466223-138466367	Yes*	chr3:138458960-138460500	*Terminal exon for shorter isoform, distinct from APA loci
Hnrnph1	chr11:50385140-50385190, chr11:50381458-50381597	No	chr11:50385777-50386528	Second to last exon
Mknk2	chr10:80671927-80672015	No	chr10:80665318-80667332	
Muc4	chr16:32763910-32763999	No	chr16:32781826-32782390	
Ngly1	chr14:16260563-16260800	No	chr14:16310859-16311537	
Ranbp2	chr10:58453311-58453423	No	chr10:58493663-58494154	
Rexo4	chr2:26960223-26960417	No	chr2:26953564-26954596	
Rnps1	chr17:24418037-24418218	No	chr17:24425086-24425897	
Slc25a17	chr15:81327216-81327362	No	chr15:81318921-81319776	
Tlk1	chr2:70786876-70786995	No	chr2:70712539-70714165	
4833439L19Rik	chr13:54559197-54559324	No	chr13:54551290-54552817	
Akap9	chr5:4028373-4028536, chr5:4003560-4003782	No	chr5:4079659-4080204	
Cc2d1a	chr8:84146313-84146449	No	chr8:84132828-84133257	

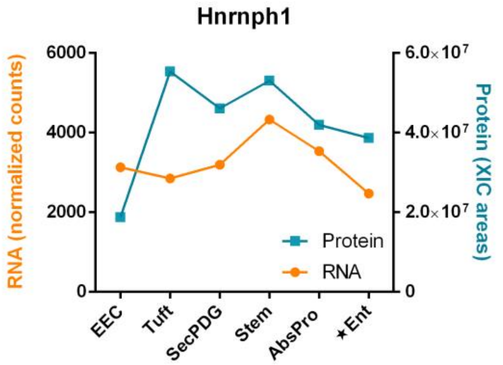

b

**Polyadenylation Events**	**Distal PolyA Usage of Replicates**	**Average Distal PolyA Usage**	**Loci Coordinates**	**Padj (Compared with Stem)**	**Gene Expression Change (Compared with Stem)**
Sae1	Stem: 0.79,0.51,0.40	0.57	chr7:16327051-16327929		
	AbsPro: 0.21,0.18,0.80	0.40		7.66E-06	ns
	SecPDG: 0.00,0.52,0.41,0.52	0.36		1.63E-03	Padj = 0.011
Uba2	Stem: 0.42,0.75,0.46	0.53	chr7:34140697-34141466		
	AbsPro: 0.23,0.10,0.54	0.29		4.51E-08	ns
	SecPDG: 0.09,0.52,0.23,0.48	0.33		4.32E-05	ns
**Splicing Events**	**Inclusion Rates of Replicates**	**Average Inclusion Rate**	**Exon Coordinates**	**FDR (Compared to Stem)**	**Gene Expression Change (Compared with Stem)**
Sae1	Stem: 1.00, 1.00, 1.00	1.00	chr7: 16327830-16327929		
	AbsPro: 0.273, 1.00, 1.00	0.76		1.80E-02	ns
	SecPDG: 1.00, 1.00, 0.867	0.96		ns	Padj = 0.011

c

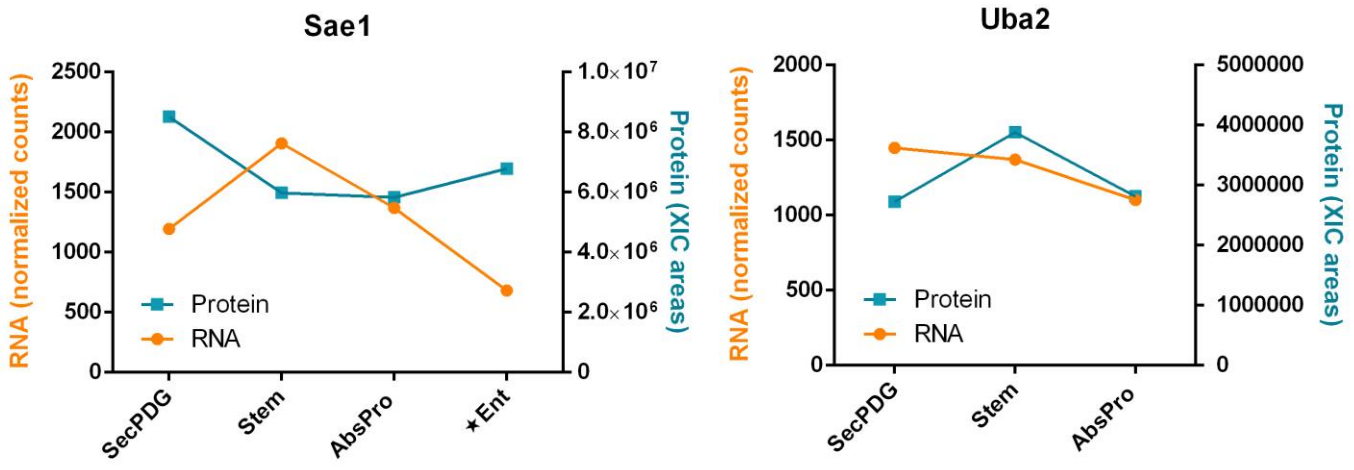

Supplementary Figure 21: Common mRNA processing events. **a** Table shows the n=13 genes (in Figure 4b Venn diagram) that are both alternatively spliced and polyadenylated in stem versus AbsPro and stem versus SecPDG. Coordinates for the events are given and show there is not an overlap in the processing event location in each gene confirming the splicing and polyadenylation events are distinct. Hnrnph1 was detected by MS, and the protein and mRNA levels are shown in the graph (Right). **b** Ontology analysis (Figure 4c) shows that SUMOylation processes impacted by both splicing and polyadenylation. The polyadenylation and splicing events of *Sae1* and *Uba2* are shown in the table. **c** mRNA and protein expression (when detected) for Sae1 and Uba2 are graphed. Star annotation by cell type symbolizes significant differential mRNA expression compared to stem (padj < 0.01). For differential mRNA expression and processing analyses, the following biological replicate numbers were used: stem=3, AbsPro=3, SecPDG=4, tuft=5, Ent=5, and EEC=2.

SUPPLEMENTARY FIGURE 22

a

Splicing Events	Inclusion Rates of Replicates	Average Inclusion Rate	Exon Coordinates	FDR (Compared to Stem)	Gene Expression Change (Compared with Stem)
Ctnnd1	Stem: 0.954,0.786,0.837	0.86	chr2: 84605183-84605246		
	AbsPro: 0.531,0.179,0.49	0.40		2.98E-08	ns
	SecPDG: 0.573,0.696,0.181,0.598	0.51		2.91E-05	ns
Spen	Stem: 0.145,0.976,0.57	0.56	chr4: 141485558-141485672		
	AbsPro: 1.0,1.0,1.0	1.00		4.32E-06	ns
	SecPDG: 1.0,0.916,0.952,0.94	0.95		7.45E-04	ns
Cbfa2t2	Stem: 1.0,1.0,0.9	0.97	chr2: 154500399-154500543		
	AbsPro: 0.449,0.0,0.57	0.34		8.56E-08	ns
	SecPDG: 0.874, 0.55	0.71		ns	ns
Eif4a2	Stem: 0.467,0.388,0.362	0.41	chr16: 23112350-23112457		
	AbsPro: 0.578,0.337,0.462	0.46		ns	ns
	SecPDG: 0.0,0.13,0.072,0.137	0.08		0.00E+00	ns

b

Polyadenylation Events	Distal PolyA Usage of Replicates	Average Distal PolyA Usage	Loci Coordinates	Padj (Compared with Stem)	Gene Expression Change (Compared with Stem)
Top2a	Stem: 0.1,0.12,0.36	0.19	chr11:98992947-98993413		
	AbsPro: 0.54,0.58,0.36	0.49		1.07E-05	ns. Padj=0.06, log2fc = 1 (elevated in Stem)
	SecPDG: 0.18,0.5,0.8,0.35	0.46		1.88E-05	ns
Wdhd1	Stem: 0.19,0.21,0.11	0.17	chr14:47240944-47241812		
	AbsPro: 0.74,0.54,0.22	0.50		1.08E-03	ns
	SecPDG:1,0.16,0.38	0.51		9.70E-07	ns
Cby1	Stem: 0.16,0.55,0.42	0.38	chr15:79666988-79667660		
	AbsPro: 0.81,0.79,0.65	0.75		6.31E-09	ns
	SecPDG:1,0.5,0.59,0.48	0.64		4.95E-03	ns
Rbm3	Stem: 0.21,0.63,0.63	0.49	chrX:8142356-8142955		
	AbsPro: 0.7,0.93,0.78	0.80		9.83E-16	ns
	SecPDG: 0.31,0.67,0.92,0.59	0.62		ns	ns
Ihh	Stem: 0.37,0.41,0.45	0.41	chr1:74945315-74946747		
	AbsPro:0.66,0.35,0.47	0.49		ns	ns
	SecPDG: 0.45,0.62,0.8,0.7	0.67		9.66E-06	ns

Supplementary Figure 22: Expression and processing data for examples of alternatively processed genes. **a** Two splicing events that occur in both stem versus AbsPro and stem versus SecPDG (*Ctnnd1* and *Spen*), and two processing events that are unique to either stem versus Abspro (*Cbfa2t2*), or stem versus SecPDG (*Eif4a2*). Table lists inclusion rates for the skipped exon, the average rates of the inclusion, coordinates of skipped exon along with the splicing event significance. No significant changes in mRNA levels occurred for any of these genes when compared to the stem cell population. **b** Three alternative polyadenylation events that occur in both stem versus AbsPro and stem versus SecPDG (*Top2a, Wdhd1,* and *Cby1*) are listed. Two events that are unique to stem versus AbsPro (*Rbm3*) or are unique to stem versus SecPDG (*Ihh*) are also listed. Table includes rates of distal polyA usage in replicates, average distal polyA use, chromosome location of the polyA event within the gene locus, and polyA event significance. No significant gene expression (mRNA level) change occurred in any of these genes compared with stem, although *Top2a* expression was increased in stem. For differential mRNA expression and processing analyses, the following biological replicate numbers were used: stem=3, AbsPro=3, SecPDG=4, tuft=5, Ent=5, and EEC=2.

SUPPLEMENTARY FIGURE 23

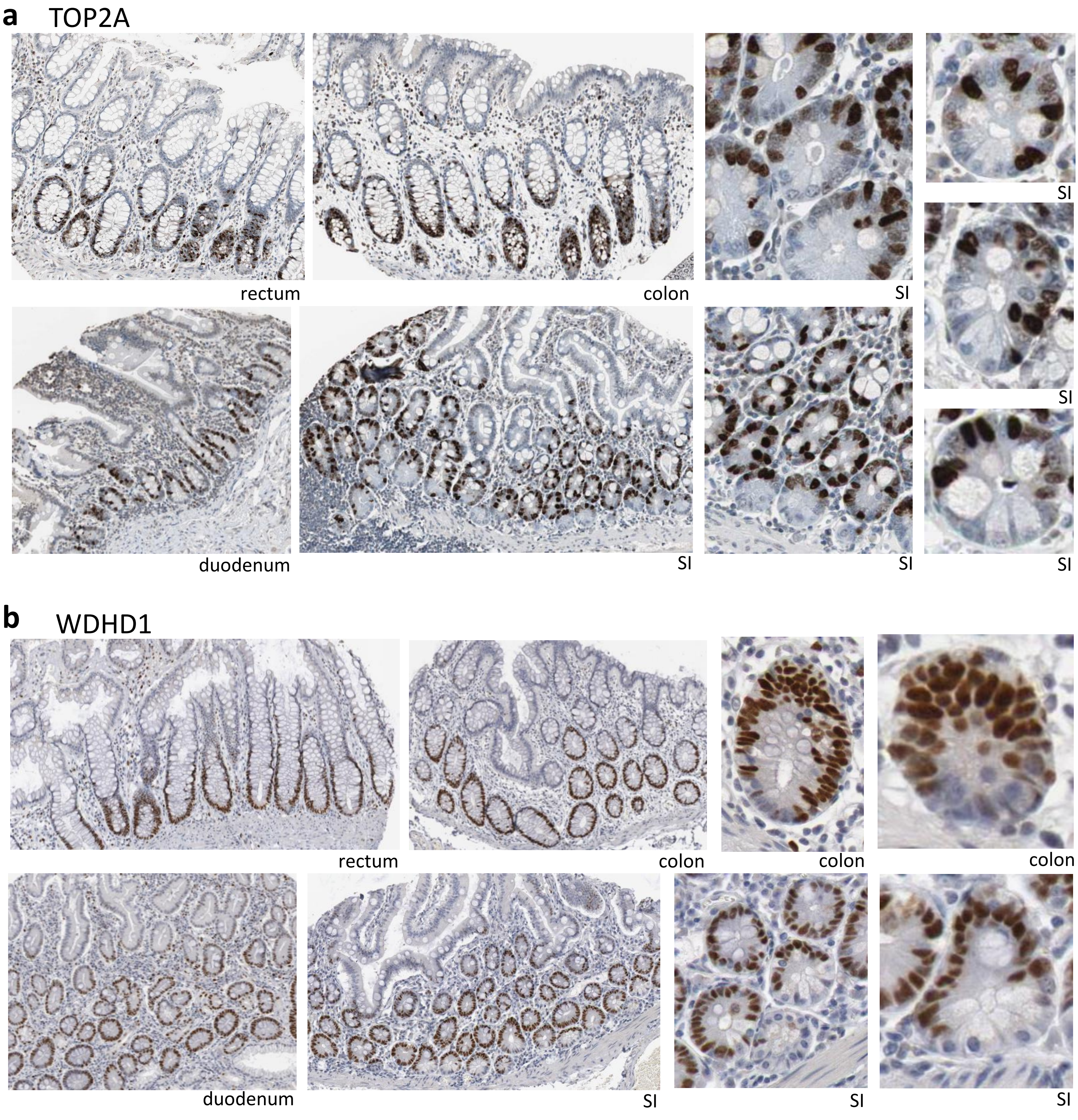

Supplementary Figure 23: Intestinal staining of alternatively polyadenylated genes in human intestinal tissues. Both **a** *Top2a* and **b** *Wdhd1* were defined as alternatively polyadenylated in stem versus AbsPro and stem versus SecPDG in mouse colon. Even though *Wdhd1* and *Top2a* (padj = 0.06) had modest increases in mRNA levels in stem, minimal staining was observed at the base of the stem cell niche. Both TOP2A and WDHD1 staining appear to be limited to the transit amplifying zone with no evidence of expression in differentiated cells towards the top of the crypt.

SUPPLEMENTARY FIGURE 24

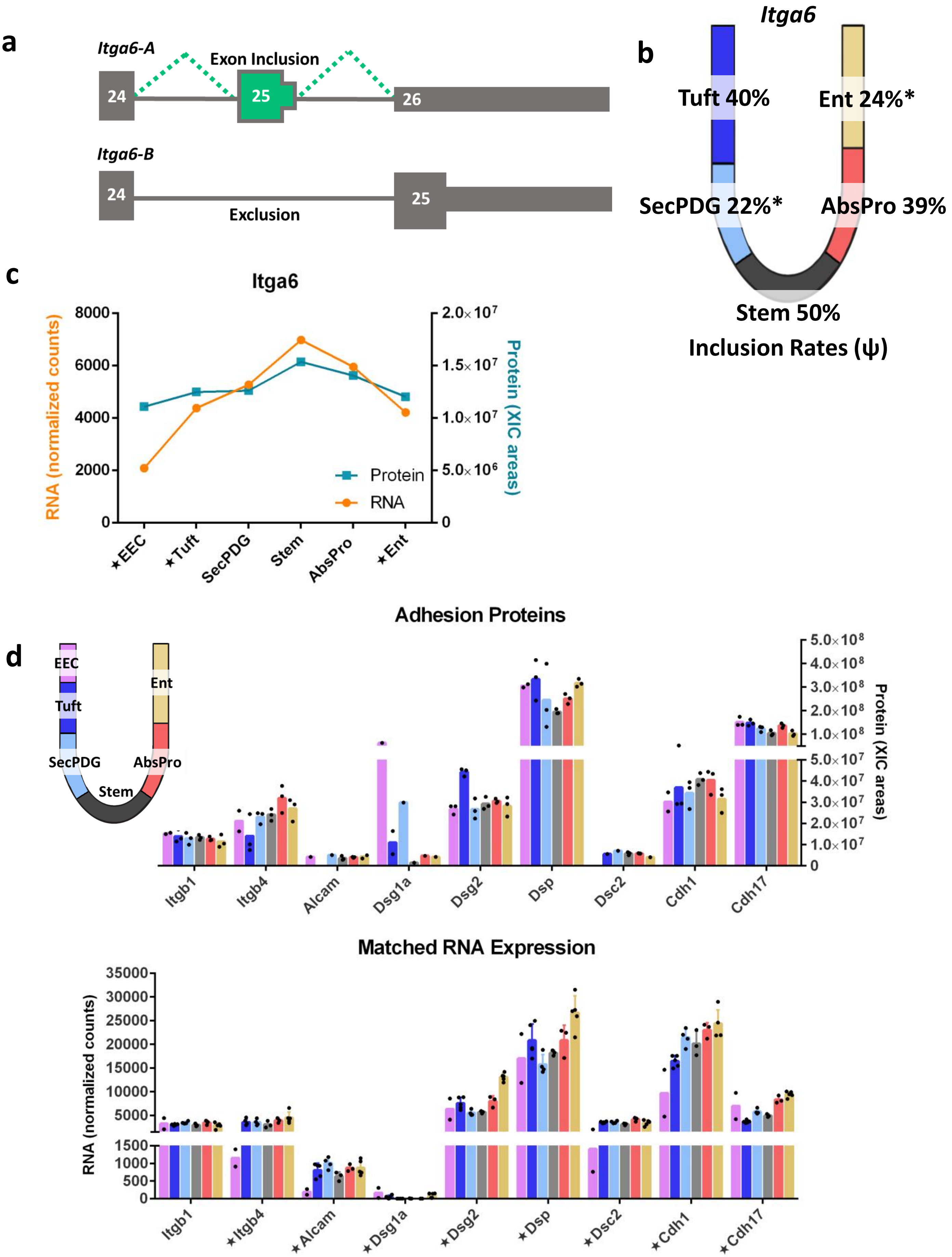

Supplementary Figure 24: Expression and splicing of adhesion genes. **a** Alternative splicing of exon 25 in *Itga6* produces two known, distinct isoforms. **b** Exon inclusion is at its highest level in stem cells compared to progenitors and differentiated cells. Inclusion rates were lowest in SecPDG and Ent (* marks significant decrease compared to stem; FDR < 0.05). **c** Itga6 protein was detected via MS in all cell types and compared to its cognate mRNA expression. Star annotation by cell type symbolizes significant differential mRNA expression compared to stem (padj < 0.01). **d** Additional adhesion proteins were detected and matched with mRNA expression. With the exception of Dsg1a and Dsg2, the expression level of each gene is similar across all cell types. There are however, striking differences in the ratio of mRNA:protein. For example: *Dsc2* mRNA is abundant, but there is very little protein; *Cdh1* mRNA is much higher than *Cdh17*, but there is more Cdh17 protein than Cdh1. Star annotation by gene name symbolizes significant differential mRNA expression in at least one cell type compared to stem (padj < 0.01) and error bars are standard deviation. For proteomics there are n=3 independent biological replicates for each cell type; for mRNA differential expression analysis the following biological replicate numbers were used: stem=3, AbsPro=3, SecPDG=4, tuft=5, Ent=5, and EEC=2.

SUPPLEMENTARY FIGURE 25

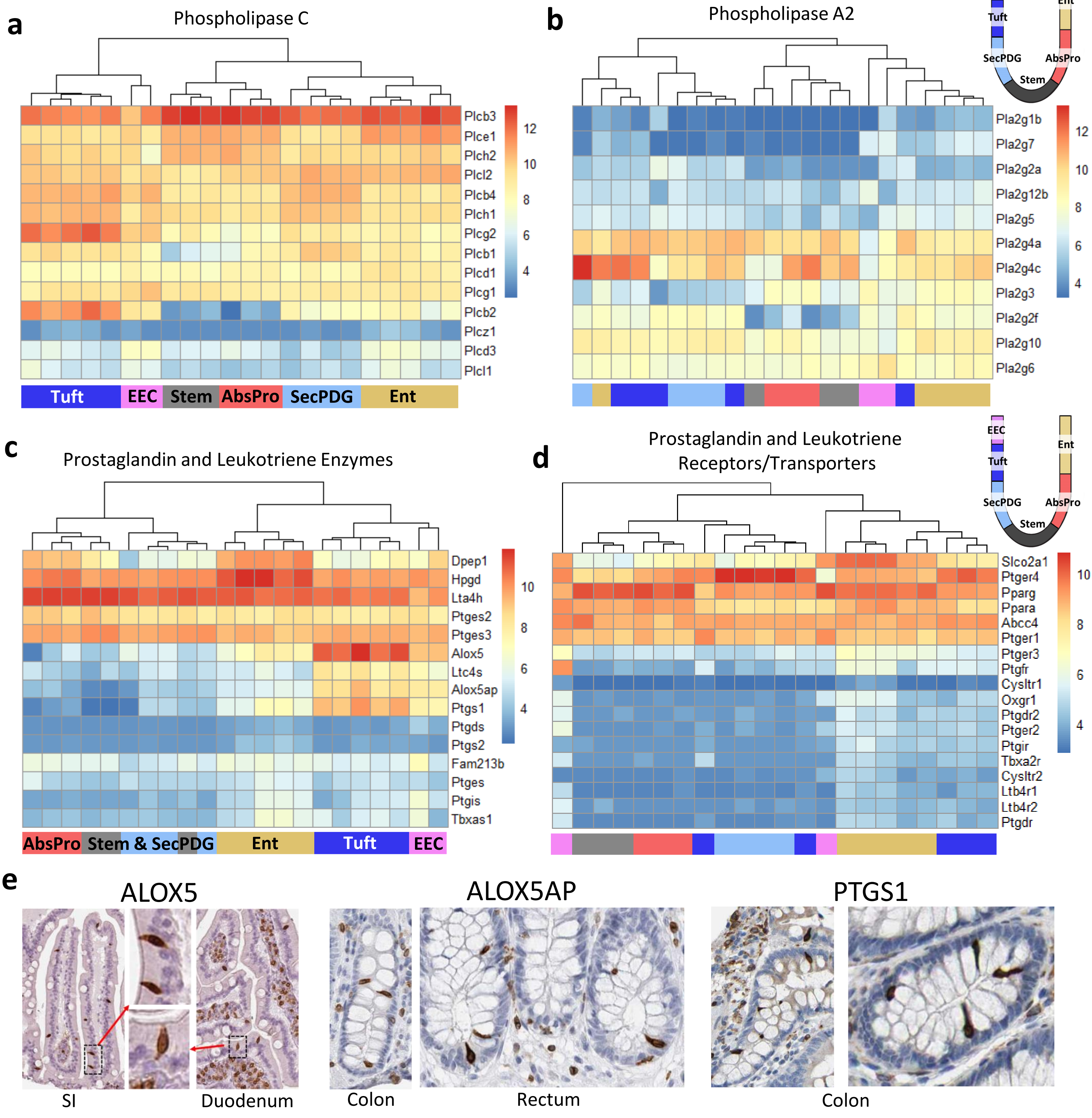

Supplementary Figure 25: Prostaglandin and Leukotriene production and signaling in the intestinal crypt. Diacylglycerol or phospholipids are converted to arachidonic acid by **a** Phospholipase C or **b** Phospholipase A2 enzymes. **c** Arachidonic acid is then provided to the leukotriene synthesis pathway (*Alox5, Alox5ap, Ltc4s*) or the prostaglandin synthesis pathway (*Ptgs1, Ptgs2*). Additional enzymes create different forms of prostaglandins and leukotrienes. **d** Prostaglandins and leukotrienes are often used to signal to non-epithelial cells, but there are some receptors and transporter expression in the crypt. **e** tuft cells are unique in the crypt epithelium in their ability to catalyze the initial conversion of arachidonic acid, which is confirmed by human protein atlas staining. See *Supplementary Discussion* section for additional information.

SUPPLEMENTARY FIGURE 26

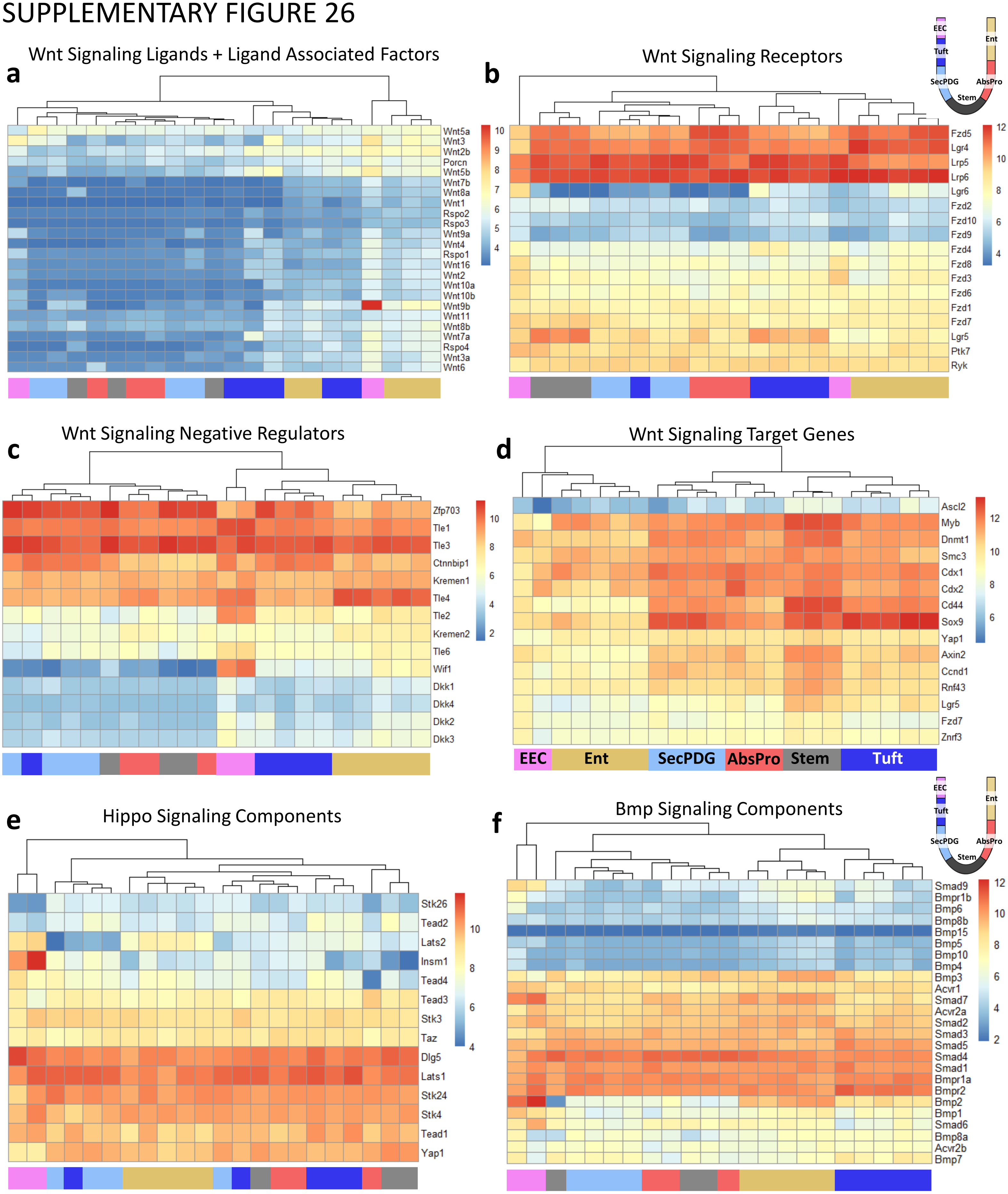

Supplementary Figure 26: Unsupervised clustering of crypt signaling pathway components. Wnt signaling **a** ligands and ligand associated factors, **b** receptors, **c** signaling inhibitors, and **d** Wnt signaling target genes. **e** Hippo signaling components. **f** Bmp signaling components.

SUPPLEMENTARY FIGURE 27

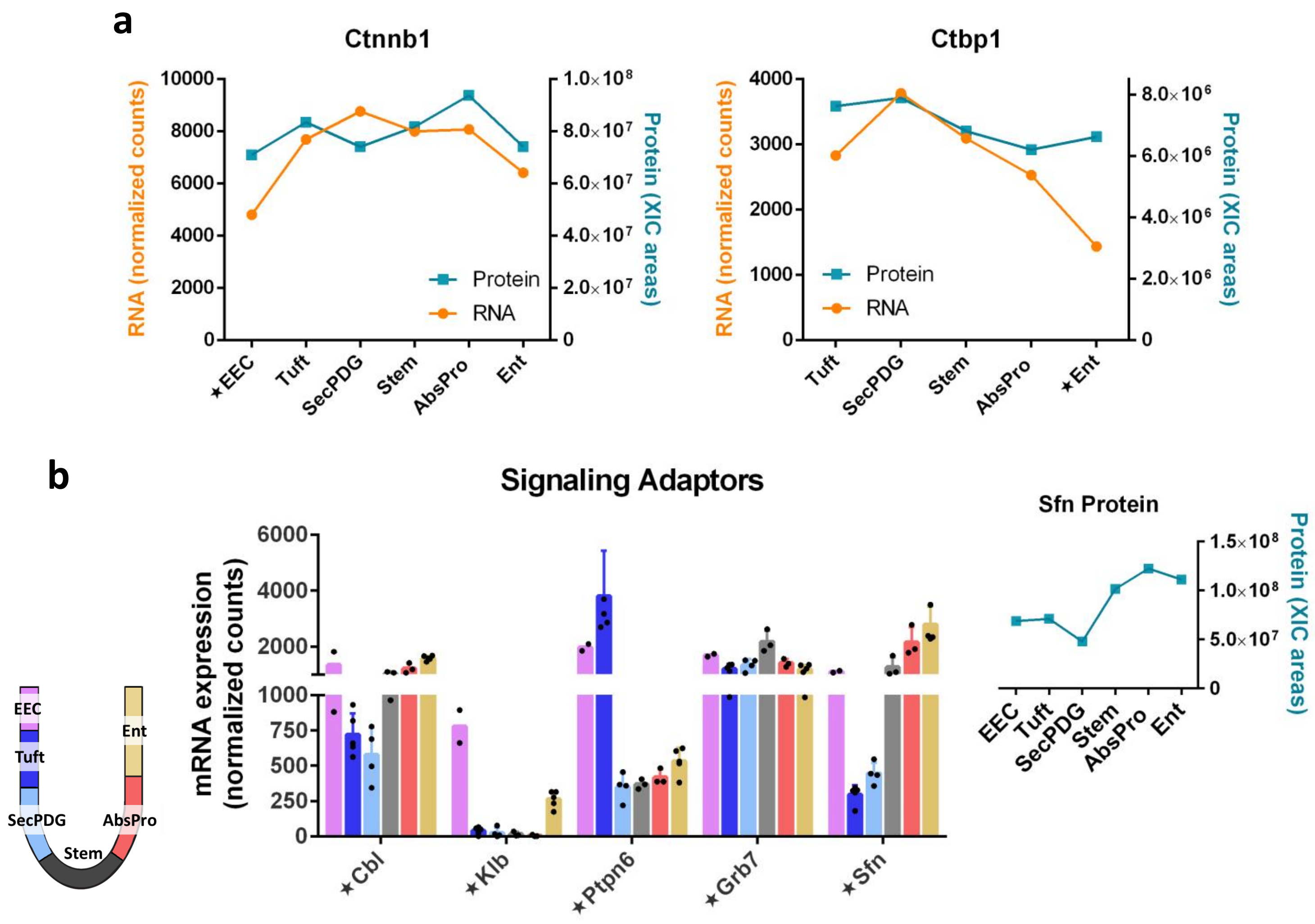

Supplementary Figure 27: Additional influences on signaling. **a** Although Wnt signaling is known to be elevated in the stem cell niche, the important mediator Ctnnb1 (β-catenin) was observed to be expressed in all cell types. Expression of co-repressor Ctbp1, which can also influence Wnt signaling, was also detected in all cell types. Star annotation by cell type symbolizes significant differential mRNA expression compared to stem (padj < 0.01). **b** Signaling adaptors can influence specific signaling pathways and we observed several that had unique expression. Cbl can influence Kit, Fgf, and Egf receptors, and Ptpn6 can also influence Kit and Egfr. We detected Sfn protein to be decreased in SecPDG (graph Right), consistent with mRNA expression. Star annotation by gene name symbolizes significant differential mRNA expression in at least one cell type compared to stem (padj < 0.01) and error bars are standard deviation. For mRNA differential expression analysis the following biological replicate numbers were used: stem=3, AbsPro=3, SecPDG=4, tuft=5, Ent=5, and EEC=2.

SUPPLEMENTARY FIGURE 28

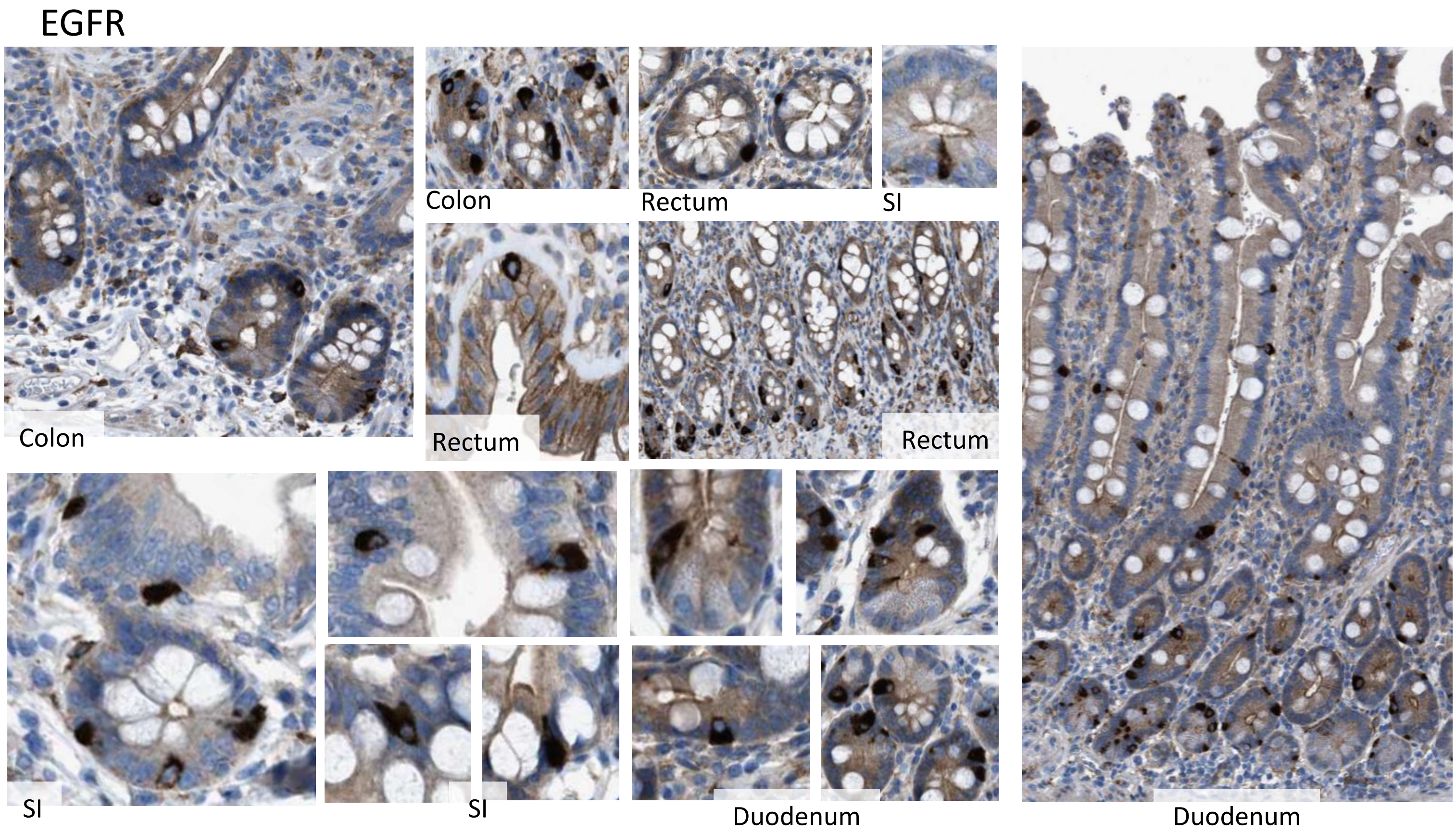

Supplementary Figure 28: Human intestinal expression of the EGF receptor (EGFR; human protein atlas). EGFR expression appears to mark tuft and EEC-shaped cells in the rectum, colon, small intestine, and duodenum.

SUPPLEMENTARY FIGURE 29

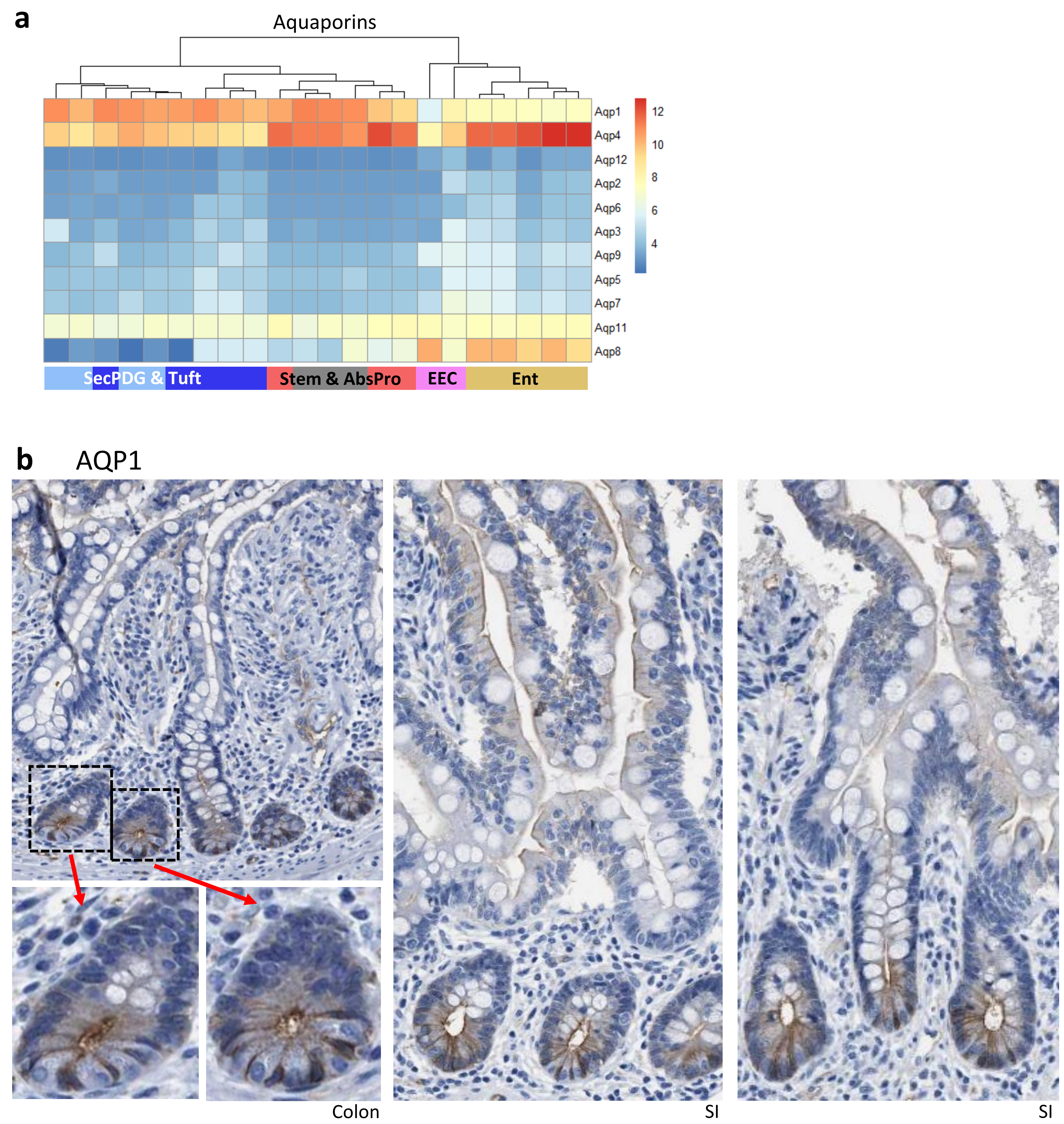

Supplementary Figure 29: Colonic expression of Aquaporins. **a** Heat map of unsupervised clustering showing expression of all aquaporin water channels. **b**. Note that AQP1 expression appears to be highly specific to stem cells at the crypt base in colon (including enlargement of crypt base), and small intestine.

SUPPLEMENTARY DISCUSSION

Discussion for Supplementary Figure 10:

Tuft cells in the small intestine function as chemo-sensory cells for “tasting” pathogens and coordinating a protective immune response. Several studies recently showed that small intestinal tuft cells have the ability to detect pathogens via the small molecule succinate due to their high expression of the succinate receptor (*Sucnr1*) and taste sensory signaling components gustducin (*Gnat3*) and *Trmp5* [3–6]. Whether tuft cells in the colon exhibit a similar chemo-sensory “taste” profile was not known. We detected high expression of taste signaling components for signal transduction in tuft cells, (*Gnat3*, *Gng13*, high expression of *Trmp5* and taste sensing signal facilitator *Plcb2*; Supplementary Figure 10a), however, there is little to no expression of Tas2 taste family receptors or the succinate receptor *Sucnr1,* (Supplementary Figure 10a,b) [3]. Tas1 taste receptors are expressed in tuft cells, but they are also expressed by all cell types and therefore not tuft cell-specific. Instead, we discovered tuft cell-specific expression of the free fatty acid receptors, *Ffar2*, *Ffar3,* and *Ffar4*, receptors that utilize the same downstream sensory signaling components as for succinate. These data indicate that colon tuft cells have a unique ability for metabolic sensing of fats, particularly short chain fatty acids.

Discussion for Supplementary Figure 25:

Arachidonic acid is the molecular precursor for both prostaglandins and leukotrienes, each of which are potent inflammatory mediators for immune responses. Interestingly, all cell populations express high levels of *Plcb3*, an enzyme capable of producing arachidonic acid (Figure 6d), but only tuft cells express the machinery needed to convert arachidonic acid to leukotriene C4 (*Alox5*, *Alox5ap* (Flap), and *Ltc4s*; Supplementary Figure 18c). Further conversion of the leukotriene C4 precursor to D4 and E4 is possible in AbsPro and Ent which express high levels of *Dpep1*. This suggests that multiple cell populations cooperate with tuft cells to synthesize the full array of leukotriene mediators of inflammation. Unlike the prostaglandin pathway, the receptors for leukotrienes (Cysltr1 and Cysltr2) are minimally expressed in the epithelia underscoring how this signal is likely meant for cell populations in the stroma [8].

SUPPLEMENTARY FIGURE REFERENCES

1. Billing, L. J. *et al.* Single cell transcriptomic profiling of large intestinal enteroendocrine cells in mice – Identification of selective stimuli for insulin-like peptide-5 and glucagon-like peptide-1 co-expressing cells. *Mol. Metab.* **29**, 158–169 (2019).
2. Haber, A. L. *et al.* A single-cell survey of the small intestinal epithelium. *Nature* **551**, 333–339 (2017).
3. Nadjsombati, M. S. *et al.* Detection of Succinate by Intestinal Tuft Cells Triggers a Type 2 Innate Immune Circuit. *Immunity* **49**, 33–41 (2018).
4. Gerbe, F., Legraverend, C. & Jay, P. The intestinal epithelium tuft cells: specification and function. *Cell. Mol. Life Sci.* **69**, 2907–2917 (2012).
5. Howitt, M. R. *et al.* Tuft cells, taste-chemosensory cells, orchestrate parasite type 2 immunity in the gut. *Science* **351**, 1329–33 (2016).
6. Schneider, C. *et al.* A Metabolite-Triggered Tuft Cell-ILC2 Circuit Drives Small Intestinal Remodeling. *Cell* **174**, 271-284.E14 (2018).
7. Lee, S. W. *et al.* SUMOylation of hnRNP-K is required for p53-mediated cell-cycle arrest in response to DNA damage. *EMBO J.* **31**, 4441–4452 (2012).
8. Savari, S., Vinnakota, K., Zhang, Y. & Sjölander, A. Cysteinyl leukotrienes and their receptors: bridging inflammation and colorectal cancer. *World J. Gastroenterol.* **20**, 968–77 (2014).
