# Supplementary material for: Transcriptomic and proteomic signatures of stemness and differentiation in the colon crypt
Source: Commun Biol. 2020 Aug 19;3:453. doi: 10.1038/s42003-020-01181-z (PMC7438495; doi:10.1038/s42003-020-01181-z)
Supplement: Supplementary file 6 — Description of Additional Supplementary Files [file 42003_2020_1181_MOESM6_ESM.pdf]

Description of Additional Supplementary Files

File Name: **Supplementary Data 1**
Description: Global mRNA Gene Expression

File Name: **Supplementary Data 2**
Description: Global Protein Expression

File Name: **Supplementary Data 3**
Description: Marker Gene Lists (mRNA)

File Name: **Supplementary Data 4**
Description: List of Alternatively APA+Spliced Genes
