# Supplementary material for: Transcriptomic and proteomic signatures of stemness and differentiation in the colon crypt
Source: Commun Biol. 2020 Aug 19;3:453. doi: 10.1038/s42003-020-01181-z (PMC7438495; doi:10.1038/s42003-020-01181-z)
Supplement: Supplementary file 8 — Peer Review File [file 42003_2020_1181_MOESM8_ESM.pdf]

Editorial Note: *This manuscript has been previously reviewed at another Nature Research journal. This document only contains reviewer comments and rebuttal letters for versions considered at Communications Biology*

REVIEWERS' COMMENTS:

Reviewer #1 (Remarks to the Author):

By combining transcriptional and global proteomic profiling the authors have substantially improved their manuscript. I feel that it is now suitable for publication in Communications Biology.

Minor comment:

There is mislabelling in Supplement 9 figure C. colon should be small intestine.

Reviewer #2 (Remarks to the Author):

The authors have addressed my concerns regarding clarity and have added additional data to increase the impact of this work.
